# Stability Mechanisms of a Thermophilic Laccase Probed by Molecular Dynamics

**DOI:** 10.1371/journal.pone.0061985

**Published:** 2013-04-29

**Authors:** Niels J. Christensen, Kasper P. Kepp

**Affiliations:** Department of Chemistry, Technical University of Denmark, Kongens Lyngby, Denmark; University of Akron, United States of America

## Abstract

Laccases are highly stable, industrially important enzymes capable of oxidizing a large range of substrates. Causes for their stability are, as for other proteins, poorly understood. In this work, multiple-seed molecular dynamics (MD) was applied to a *Trametes versicolor* laccase in response to variable ionic strengths, temperatures, and glycosylation status. Near-physiological conditions provided excellent agreement with the crystal structure (average RMSD ∼0.92 Å) and residual agreement with experimental B-factors. The persistence of backbone hydrogen bonds was identified as a key descriptor of structural response to environment, whereas solvent-accessibility, radius of gyration, and fluctuations were only locally relevant. Backbone hydrogen bonds decreased systematically with temperature in all simulations (∼9 per 50 K), probing structural changes associated with enthalpy-entropy compensation. Approaching *T*_opt_ (∼350 K) from 300 K, this change correlated with a beginning “unzipping” of critical β-sheets. 0 M ionic strength triggered partial denucleation of the C-terminal (known experimentally to be sensitive) at 400 K, suggesting a general salt stabilization effect. In contrast, F^−^ (but not Cl^−^) specifically impaired secondary structure by formation of strong hydrogen bonds with backbone NH, providing a mechanism for experimentally observed small anion destabilization, potentially remedied by site-directed mutagenesis at critical intrusion sites. N-glycosylation was found to support structural integrity by increasing persistent backbone hydrogen bonds by ∼4 across simulations, mainly via prevention of F^−^ intrusion. Hydrogen-bond loss in distinct loop regions and ends of critical β-sheets suggest potential strategies for laboratory optimization of these industrially important enzymes.

## Introduction

Laccases are multicopper oxidases (MCO) [1], [2], found in fungi, bacteria, plants, and insects, with exquisite temperature- and pH-tolerance and capable of oxidizing a wide array of organic and inorganic substrates [3], [4]. As such, and due to their clean reactions having only water as byproduct, they are very attractive as industrial biocatalysts [5], [6]. They have also attracted considerable interest due to their special structure-function correlations, notably the tuning of the high redox potentials and the oxygen activation mechanism that reduces dioxygen to produce water [1], [7].

Laccases usually consist of three domains (denoted D1, D2, and D3), each having a Greek-key β-barrel structure, the cupredoxin fold [8]. D1 contains a blue-copper T1 site, where electrons are abstracted from substrates, and D1 and D3 each contribute four histidines to the formation of the tri-nuclear copper site comprised of a T2 and a T3 copper site. One electron is sequentially abstracted from each of four substrate molecules at the T1 site and then transported to the T2 and T3 sites where dioxygen is reduced to two water molecules [1], [7].

Some of the most promising and well-characterized laccases are from the fungus *Trametes versicolor* [9] that degrade polycyclic aromatic hydrocarbons and a variety of phenolic compounds including polychlorophenols [9]. Based on phylogenetic analysis, a division of *Trametes versicolor* laccases (TvL) into four classes denoted α, β, γ, and δ has been suggested [9]. Biochemical characterization has subsequently shown that α- and β-laccases possess higher thermostability but lower activity relative to the γ and δ laccases [5]. These observations could point to an important case of stability-function trade-off in protein evolution [10], [11] suitable for an organism handling various substrates at variable extracellular conditions. The technological potential of laccases has inspired work towards understanding and improving their (unfortunately often anti-correlated) activity and thermostability [6], [12], [13].

Protein thermostability is of both fundamental and industrial importance [10]: Thermostable enzymes allow high process temperatures with associated higher reaction rates and less risk of microbial contamination [14], [15], [16]. The molecular determinants of protein thermostability are not well-understood and cannot be generalized across protein families [17], in particular given the complex effects of the chemical environment, e.g. temperature (T), pH, solvent polarity and composition, and ionic strength (IS), and post-translational modifications [18] **,** notably glycosylations widely occurring in eukaryote proteins [19], on protein structure, dynamics, and stability. Still, it is generally accepted that some stability drivers are relatively common, i.e. compact packing [20], secondary-structure formation, the associated presence of turns, optimization of buried side chains, e.g. disulfide bridges, salt bridges and buried polar interactions, [21], [22] burying of hydrophobic residues [10], [23], and in some cases solvent-exposed residues e.g. for anchoring loose ends such as N- and C-termini and loops [10], [24], [25]. In the context of thermostability, enthalpy-entropy compensation is orders-of-magnitude larger than the resulting free energy of folding and is as important for understanding thermostability as the free energy of folding itself [26], [27], [28], [29].

The state-of-the-art computational method for obtaining structural and dynamical information about proteins relevant for understanding these issues is molecular dynamics [30], [31], [32] (MD). MD, despite its three fundamental issues of sampling efficiency, simulation-system physics (e.g. the realism of system sizes, solvent compositions, boundary conditions, and long-range electrostatics), and force-field quality, may contribute to our understanding of protein stability: MD has been used to guide cystine introduction to enhance the thermostability of haloalkane dehalogenase [33] and to analyze the relative stability and activity of mesophilic and thermophilic subtilisin homologs [34], the thermostability of nitrile hydratases [35], and the effect of explicit counter ions in protein dynamics [36] and structural stabilization [37]. MD has also been used to rationally incorporate stability-enhancing ion-pairs into adenylate kinases [38] and to highlight flexible sites suitable for stabilization by glycine-to-proline mutations in methyl parathion hydrolase [39]. A limited number of MD studies of laccases have been performed, using single environmental parameters to rationalize structural drivers of stability [40], [41].

The poorly understood relationship between glycosylation and stability has also been explored with MD [42], [43], [44]: Notably, *N*-glycosylation has been found to increase the α-helical content relative to non-glycosylated human prion protein [44]. Comparative MD simulations [43] of glycosylated and non-glycosylated forms of the lectin EcorL from *Erythrina corallodendron* showed more consistent intra-backbone hydrogen bonds and smaller nonpolar solvent accessible surface for the glycosylated protein, supporting the experimental observations [45]. A recent MD study [46] found that fucosylation of the serine protease inhibitor *Pars intercerebralis Major Peptide-C* (PMP-C) reduced thermal flexibility of protease sites, but did not give clues to the protein-structural effects on stability.

This paper reports an investigation of the molecular drivers of the thermostability of the widely explored α-isoform of TvL [47], denoted here TvLα, using a multiple-seed approach designed to reduce systematic MD errors and monitor structural response under conditions resembling more an experimental assay optimization, notably T, IS, and glycosylation status, instead of the usual fixed conditions of standard MD simulations. We demonstrate that such a protocol can provide robust molecular insight into the state-specific changes occurring in proteins as a response to their environment, and in this way we observe several potentially important correlations between secondary structure integrity and both T, IS, and glycosylation.

We identify several determinants of thermostability in the protein, including i) a small anion (F^−^) destabilization effect; ii) a glycosylation effect on secondary-structure persistence, and iii) a quantification and residual localization of secondary-structure interactions sacrificed as a result of the entropy-enthalpy compensation at high T, with ca. nine persistent backbone-backbone hydrogen bonds lost per 50-K increase. The simulations identify several approaches to optimize stability in fungal laccases: Preservation of the C-terminal loop and sensitive ends of β-sheets, modification of several other distinct loop regions, preservation of several identified critical glycosylation sites, and prevention of small anion (F^−^) intrusion while preserving the general salt stabilizing effect by using moderate-to-high ionic strength of medium-sized (e.g. NaCl) ions. While these effects are significant from the statistics across all 30 MD seeds, they act both locally (fully analyzed in the Supporting Information, File S1) and globally to preserve secondary-structure interactions via the combination of relatively small, cumulative effects.

## Methods

### Model System Preparation

The crystal structure of the laccase (PDB ID: 1GYC [47]) was retrieved from the protein data bank [48] and prepared for MD simulations using the protein preparation wizard of Maestro [49], [50]. Small organic molecules and all crystal waters more than 5 Å from hetero atoms were deleted, and hydrogen atoms added. There are many post-translational modifications and several laccase redox states (oxidized, native, reduced, peroxy-intermediate [7]) that differ substantially in Cu sites and would make any particular choice of MD “state” and associated force field parameters somewhat arbitrary. The present simulations should probe the structural and dynamic properties of the protein (not the redox potential, electron transfer rate, or substrate affinity). Thus, instead of one simulation with specific state-parameters as typically done, we have performed several 10+20 ns (NPT+NVT) simulations to validate the convergence and robustness of the conformational space investigated and in addition 30 shorter (10+3 ns) simulations or “seeds” with changed external conditions as a different “response” approach to the problem, compared to a realistic reference state of the laccase. This enables error cancellation from relative comparisons while also improving statistics. All bond-distances and angles involving copper were thus fixed as in the crystal structure, and a charge of +1 was assigned to copper with a water explicitly bound in T2/T3, reflecting best the reality of the multitude of combinations of ligand-, redox-, and protonation-states for the T2/T3 and T1 sites.

The real glycosylation state of TvLα is likely to be more complex than that observed in the crystal structure. However, any attempt infer the effect of more extensive glycosylation is arbitrary due to the lack of structural information, and the range of possible glycans and their conformational space prevents such an investigation. We therefore employed a *ceteris paribus* approach to probe the impact of the only the structurally confirmed part of the glycosylation, focusing on the direct linkage: Thus, two specific variants of TvLα were prepared and simulated, containing zero (“noNAG”) or all (“NAG”) N-acetyl glucosamine moieties at the N-glycosylation sites as observed in the X-ray structure. The identified effect of glycosylation on hydrogen bond persistence was also validated at longer simulation runs, as shown in Table S9 in File S1.

### Molecular Dynamics Simulations

The system details are listed in Table 1. Each simulation carries a name such as [NAG_1P2M_KF_400 K], where NAG or noNAG refers to the protein with or without glycosylation, 0P0M, 0P3M, and 1P2M denote the ionic strengths of either 0 M, 0.3 M, or 1.2 M, and KF or NACL refers to the salt used in the simulation. The final part denotes the temperature.

**Table 1:** System details for molecular dynamics simulations performed in this study.*

Simulation Name	# TIP3P	Neutrali-zing ions	Additional ions	Ion concen-tration (M)	# Atoms	Post-NPT Box Length (Å)
NAG_0P0M	26529	4 Na^+^	–	0.00†, 0.00‡	87309	95.73 Å
noNAG_0P0M	26621	4 Na^+^	–	0.00†, 0.00‡	87342	95.61 Å
NAG_0P3M_NACL	26584	4 Na^+^	153 Na^+^, 153 Cl^−^	0.29†, 0.29‡	87780	95.90 Å
noNAG_0P3M_NACL	26673	4 Na^+^	153 Na^+^, 153 Cl^−^	0.29†, 0.29‡	87804	95.94 Å
NAG_0P3M_KF	26584	4 K^+^	153 K^+^, 153 F^−^	0.29†, 0.29‡	87780	95.70 Å
noNAG_0P3M_KF	26673	4 K^+^	153 K^+^, 153 F^−^	0.29†, 0.29‡	87804	95.81 Å
NAG_1P2M_NACL	26181	4 Na^+^	721 Na^+^, 721 Cl^−^	1.34†, 1.40‡	87707	96.26 Å
noNAG_1P2M_NACL	26236	4 Na^+^	723 Na^+^, 723 Cl^−^	1.35†, 1.40‡	87633	96.15 Å
NAG_1P2M_KF	26181	4 K^+^	721 K^+^, 721 F^−^	1.37†, 1.40‡	87707	95.53 Å
noNAG_1P2M_KF	26236	4 K^+^	723 K^+^, 723 F^−^	1.37†, 1.40‡	87633	95.61 Å

* All ten simulations were in addition performed independently at T = 300, 350, and 400 K, giving a total of 30 simulations of 10 ns NPT ensemble +3 ns NVT ensemble.

† Background ionic concentration based on total cell volume.

‡ Background ionic concentration based on water volume.

Systems were simulated with zero, intermediate NaCl or KF, or strong NaCl or KF ionic strengths performed at each of three temperatures (300, 350, and 400 K) and as either glycosylated or non-glycosylated protein, including all combinations possible (30 systems altogether). 350 K is approximately the T_opt_ of the laccase for oxidation of ABTS [5], i.e. we probed the protein at T_opt_, T_opt_ −50 K, and T_opt_ +50 K. All minimizations and MD simulations were carried out with Desmond 3.0 [51] using cubic simulation boxes of TIP3P [52] molecules with a minimum layer of 13 Å of water on each side of the protein. Protein charge was neutralized by replacing randomly selected waters with 4 Na^+^ counter-ions (corresponding to a ∼10 mM Na^+^ concentration). For higher ionic strengths, ion pairs were added as an ionic background to the simulation box, either 153, 721, or 723 of each anion and cation, corresponding to concentrations (ionic strengths) of 0, 0.29, and 1.40 M based on water volume, or 0, 0.29, and 1.35 M based on total simulation cell volume. For the KF simulations, the ionic parameters (K^+^, F^−^) replaced the (Na^+^, Cl^−^) parameters in Desmond structure/parameter (.cms) files for NaCl simulations, to ensure that simulations in NaCl and KF had identical starting geometries. The OPLS-2005 force-field [53] was used with associated carbohydrate parameters [54] for NAG and the free-energy consistent alkali- and halide-parameters [55] for K^+^, Na^+^, Cl^−^, and F^−^.

Each system in Table 1 was minimized by steepest-decent to a gradient of 1 kcal mol^−1^ Å^−1^ followed by the default pre-simulation protocol consisting of (1) minimization with restraints on solute, (2) unrestrained minimization, (3) Berendsen [56] NVT-simulation at T = 10 K with small time steps and restraints on heavy solute atoms, (4) Berendsen NPT-simulation at T = 10 K with restraints on solute heavy atoms, (5) Berendsen NPT-simulation with restraints on heavy solute atoms, and (6) unrestrained Berendsen NPT-simulation.

Following the relaxation protocol, a 10-nanosecond (ns) NPT-simulation was carried out for each system at 300 K (the RMSD curves can be found in File S1, Figures S1 and S2). Subsequently, the structure at the last time step of each NPT simulation was used as the starting structure for additional NVT-simulations at different temperatures (300 K, 350 K, and 400 K). The temperature in the NPT- and NVT-simulations was regulated with the Nose-Hoover chain thermostat [57] with a relaxation time of 1.0 ps, and the pressure in the NPT-simulations was regulated with the Martyna-Tobias-Klein [58] barostat with isotropic coupling and a relaxation time of 2.0 ps. The RESPA [59] integrator was employed with bonded-, near-, and far-time steps of 2.0 fs, 2.0 fs, and 6.0 fs, respectively. A 9-Å cutoff was used for non-bonded interactions together with the smooth-particle-mesh Ewald method [60] with a tolerance of 10^−9^ was used for long-range Coulomb interactions. A notable advantage of using realistic ionic strengths, beyond the realism itself, is, together with the NVT-pre-simulation, the short simulation times to obtain equilibrium hydrogen bond counts (with short time scales) central to this work (in contrast, many standard simulations with inefficient pre-equilibration or only neutralizing ionic strength fail to equilibrate even over microsecond runs).

MD trajectories were saved to disk at 20 ps intervals, and several properties were calculated from the last 30×3-ns NVT trajectories using VMD [61] and in-house Tcl scripts. Time series were produced for radius-of-gyration (R_gyr_), backbone hydrogen bonds, solvent accessible surface area (SASA), and the root-mean-square backbone atomic positional deviation (RMSD) with respect to the crystal structure [47]. For SASA, time series were calculated using a 1.4-Å spherical probe and evaluated only for the protein part of the macromolecule. Time series for secondary structure were calculated using the VMD implementation of STRIDE [62], which assigns secondary structure based on hydrogen-bond energy and statistically derived backbone torsional angle information. The averages and standard deviations were evaluated for all time series, and the averages were tested for correlation with the environmental perturbations employed. The statistical persistence of each hydrogen bond in the simulations was calculated with the VMD HBonds Plugin, Version 1.2. Radial distributions for amide hydrogen and halide ions were used to probe structural interactions relating to salt effects and are shown in File S1, Figures S34−S39.

The average laccase structure was calculated for the last two ns of each NVT simulation after superimposition of the backbone atoms of all frames to the initial frame. Root-mean-square positional fluctuations (RMSF) were calculated for backbone atoms. To allow comparison with the crystal structure, RMSF values were converted to B-factors [63] using Equation 1:
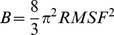
(1)


Only the B-factor averaged over non-hydrogen backbone atoms was considered for each residue. The B-factor plots of the simulations can be found in File S1, Figures S40−S49.

### Monitoring Specific Interactions

Backbone hydrogen bonds were counted using two different approaches. The first approach counted the average number of backbone hydrogen bonds in each NVT simulation. This was done by summing the number of Donor-Hydrogen-Acceptor (D-H-A) interactions in each MD frame and dividing the sum by the total number of MD frames considered. A D-H-A interaction counted as a hydrogen bond provided that the distance D-A <3.5 Å and the angle between the vectors D-H and D-A <30°. These geometric criteria are similar to those employed in earlier studies of hydrogen bonds in proteins using the OPLS force field [64]. In addition, to validate the robustness of the analysis, the number of *persistent* backbone hydrogen bonds was also counted for each simulation using the same detection criteria as before, but now requiring that these geometric criteria were fulfilled for >50% of the simulation time.

Side-chain salt bridges were detected with the VMD salt-bridges plugin, Version 1.1. Initially, a salt bridge was counted if the distance between any carboxylate oxygen of Asp/Glu or any nitrogen of Lys/Arg was <3.2 Å in at least one MD frame. The resulting pre-screening was subsequently filtered using the more restrictive persistence criteria of counting only if the distance between the center of mass of the oxygens in the carboxylates and the center of mass of the nitrogens in the positively charged side-chain was <3.5 Å for more than 50% of the simulation time (see File S1, Tables S2−S5). Using these criteria was found to be a robust way of monitoring significant changes in hydrogen-bonding and salt bridges during simulations under the various studied system conditions, to be discussed in detail below.

### Properties of the Studied Systems

Average, standard-deviation, minimum- and maximum values of the SASA (Å^2^), R_gyr_ **(**Å), backbone hydrogen bond count, and RMSD relative to the crystal structure (Å) of each system are shown in Table S1 of File S1, and the time series for these properties are given in the corresponding Figures S3−S33 in File S1. Variations in SASA, R_gyr_, and RMSD were generally well-behaved but small and did not correlate consistently with the strength of any of the perturbations inspected, except the RMSD in the salt-free glycosylated protein at 400 K (NAG_0P0M_400 K) to be discussed further below.

**Figure 1:**
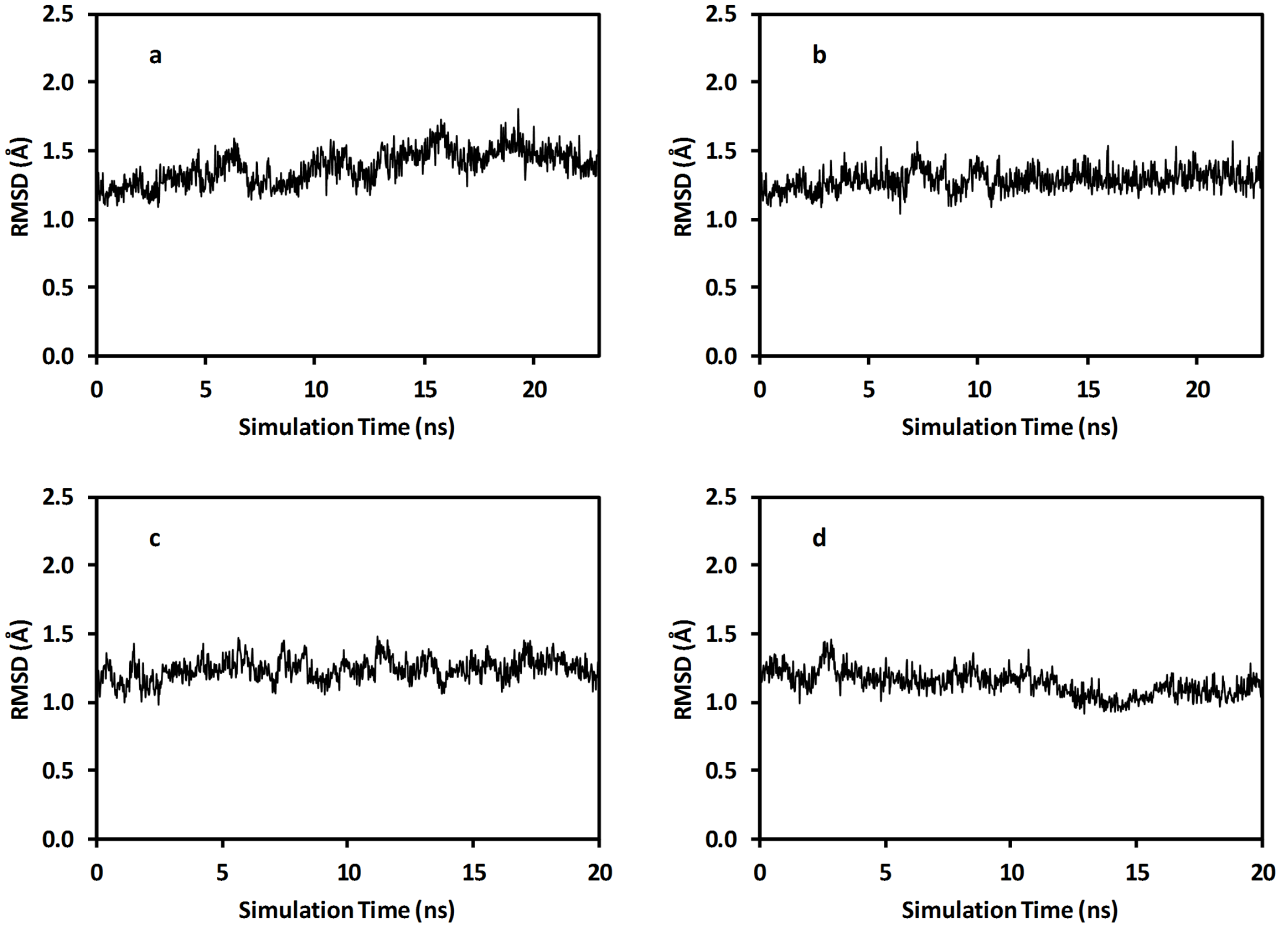
Validating the robustness of the conformations probed in the reference simulations. Four different seeds were used to produce 23-ns and 20-ns simulations for the same 300 K, 0.3 M NaCl glycosylated state serving as reference for other data (the two 23 ns simulations were started from a prequilibrated, well-behaved 3-ns simulation with different starting velocities).

MD simulations, regardless of simulation time, will run into regimes of phase space where they probe new conformations - this is the statistical nature of such methods. Thus, instead of running single, very long MD simulations, we have investigated many short runs that are well-behaved (i.e. converged in the same conformational space), to use them in combination for better confidence in observed trends. To validate our methodology and check if the identified trends are conserved also for longer trajectories that remain in the same conformational space, we have performed four differently seeded simulations on the physiological reference state at 300 K in 0.3 M NaCl. The trajectories, shown in Figure 1, remain stable showing that the same conformational space is probed throughout, i.e. no transitions occur in the RMSD curves.

The numerical variation in average backbone-backbone hydrogen bonds was found to be the most sensitive proxy for structural changes (more rigorous analysis in Supporting Information). Furthermore, both the average number of backbone hydrogen bonds and the number of persistent hydrogen bonds per simulation appears to be sensitive probes of temperature, with robustness and significant trends across all 30 seeded simulations, to be discussed below. For these reasons, we concluded that backbone hydrogen bond counts are reliable proxies for secondary-structure integrity as a function of temperature and other perturbations studied.

### Analysis of Electrostatic Interaction Energies

To understand the relationship between energy and structural changes in the proteins, simulation trajectories were analyzed using the energy_groups plugin in the original simulation configuration (cfg) files. In the structure (cms) files, a unique index for the i_ffio_grp_energy field was assigned for each group of atoms constituting a structural element of interest. Next, the interaction energy (the sum of the “elec_nonbonded” and “far_terms”) between each group of atoms was obtained with the Desmond vrun utility.

We then investigated the electrostatic interaction energy between all atoms in residues losing persistent hydrogen bonds from 300 K to 350 K in the 0.3 M NaCl glycosylated protein (these residues are indicated in bold in Table S6 and Table S7 of File S1). The 300 K, 350 K, and 400 K trajectories were analyzed and the electrostatic energy was averaged over the last 100 NVT snapshots, corresponding to the last 2 ns. The analysis of these interactions is shown in File S1, Figures S51−S53.

We also analyzed the electrostatic changes associated with the partial disruption of the C-terminal α-helical structure observed in the 400 K simulation of the glycosylated protein in zero ionic strength (see Figure S50 in File S1 for a structural view of these changes). The atoms of the 300 K reference and the 400 K simulation were divided into three energy groups: “water” consisting of all TIP3P molecules, “helix” consisting of the last C-terminal residues 489−499, and “protein_nohelix” consisting of the remainder of the protein. The time-series for the electrostatic interactions between these groups is shown in Figure S53 in File S1.

## Results and Discussion

### Structural Deviations of Simulated Systems from the Crystal Structure

**Figure 2:**
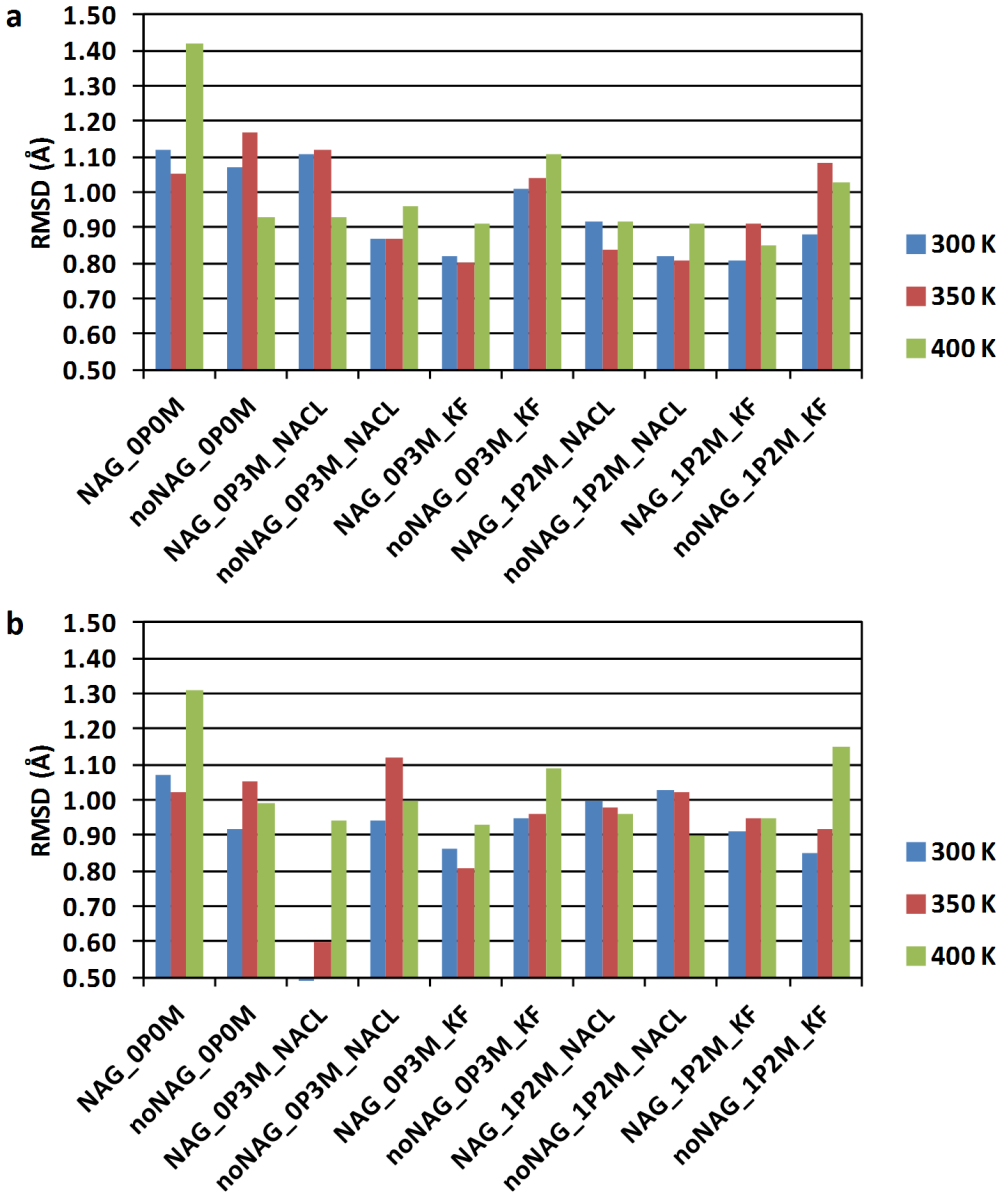
Simulation backbone RMSD (Å) relative to reference structures. (A) Backbone RMSD (Å) relative to the crystal structure for the average structure obtained from the last 2 ns of each 3 ns NVT simulation. (B) Backbone RMSD (Å) relative to the reference simulation [NAG_0P3M_NACL_300 K].

Figure 2a shows the backbone RMSD between the crystal structure of TvLα and the average structure obtained from the last 2 ns of each NVT-simulation. The RMSD ranges from 0.80 Å for [NAG_0P3M_KF_350 K] to 1.42 Å for [NAG_0P0M_400 K]. The median RMSD across all simulations of 0.92 Å shows that the backbone structure is generally well-conserved, so that it is meaningful to compare these structures. Increasing temperature, glycosylation, and ionic strength does not generally correlate with RMSD. However, the structures closest to the experimental structure are consistently from systems with conditions relatively close to physiological conditions, i.e. at T = 300−350 K and with moderate ionic strength. In contrast, the highest RMSD is observed for the [NAG_0P0M_400 K] simulation, to be discussed further below.

Most simulations with salt concentrations >0 M display smaller RMSD than the simulations with 0 M salt, notably [NAG_0P0M_400 K] and [noNAG_0P0M_350 K], which deviate the most from the crystal structure. This indicates that a realistic salt concentration is a necessary but not sufficient criterion for reproducing the experimental structure. Overall, Figure 2 shows that except for the mentioned 0 M simulations, all simulations are well-behaved and represent, to be discussed further below, similar globular states of the protein.

The crystal structure was not produced at the protein’s natural conditions, but rather in a solution containing 20%(W/V) PEG8000, 20%(V/V) isopropanol and 100 mM sodium citrate at low T (287 K), pH (5.6), and ionic strength, and with a specific post-translational modification status. Furthermore, the crystal lattice differs from the cytoplasm or extracellular environment where the laccases are active. Therefore, the RMSD analysis was repeated using as reference the [NAG_0P3M_NACL_300 K] simulation (File S1, Figure S10), which approximately corresponds to physiological conditions. Figure 2b shows the backbone RMSD evaluated against this structure. As for the comparison with the crystal structure, generally low RMSD values and stable trajectories are observed, i.e. the structures in the various simulations probe the same native globular state and are thus comparable. The most deviating structures deviate less from the reference structure in this comparison, suggesting a small, MD-specific effect, which could be due to the force field or other simulation aspect or could be a real effect of removing the crystal-solution discrepancy.

The average structure from the [NAG_0P3M_NACL_350 K] simulation has the smallest RMSD (0.6 Å) relative to the [NAG_0P3M_NACL_300 K] average structure, i.e. the protocol can probe structural information in a meaningful way. For the remaining glycosylated (NAG) simulations, those carried out in NaCl or KF background of 0.3 M provide the smallest RMSD values, confirming this observation. In contrast, the three 0 M glycosylated simulations yield a larger average RMSD, as do some of the non-glycosylated simulations at higher T. While not correlated with T, the average RMSD across temperatures is slightly but consistently larger (by up to ∼0.1 Å) for non-glycosylated protein, and this effect is robust, i.e. not caused by outliers, indicating a real effect of glycosylation on secondary structure. Altogether, these observations indicate that the laccase structures respond to changing conditions in a meaningful and significant way.

### Comparison of B-factors for Crystal Structure and Simulated Systems

**Figure 3:**
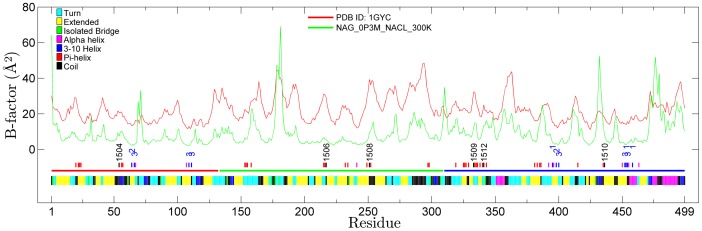
Overlay of crystal structure (red) and calculated (green) B-factors for TvLα. Calculated B-factors are from the reference simulation (glycosylated protein, 0.3 M NaCl). The bottom bar indicates secondary structure, with color codes in the upper-left legend. The horizontal three-colored line immediately above the secondary structure bar denotes the three laccase domains (D1: Red, D2: Green, D3: Blue). Above the domain line, short black vertical lines indicate NAG-positions, and red lines denote residues 4.5 Å from NAG. Blue and magenta lines indicate residues directly coordinating Cu and immediate structural neighbors of Cu-binding residues, respectively. Black vertical text denotes NAG residue numbers and blue vertical text denotes copper sites (1, 2, and 3 for T1, T2, and T3).

Root-mean-square fluctuations (RMSF) calculated from MD simulations have been used as a measure of protein structural integrity [65]. The relationship between RMSF and B-factor (Eq. 1) allows comparison between fluctuations in MD simulations and crystal structures. Backbone B-factor plots obtained for all MD simulations are included in File S1 (Figures S40−S49). Comparison of experimental and calculated B-factors shows that fluctuations in the simulations generally occur at the same sequence positions as in the TvLα crystal structure. The agreement is illustrated in Figure 3, which compares the B-factors from the simulation of the glycosylated protein in 0.3 M NaCl (corresponding roughly to physiological conditions) to those from the TvLα crystal structure. The baseline for crystal structure fluctuations is shifted higher relative to MD simulations as expected, since several effects increase observed baseline level disorder in the crystal [66], [67]. Fluctuations are generally larger and deviate more from the crystal B-factors in turns and loops, consistent with their looser modes and larger susceptibility to crystal packing forces, whereas secondary-structure elements (Figure 3, yellow/pink colors) and copper sites (blue numbers) display the lowest disorder.

**Figure 4:**
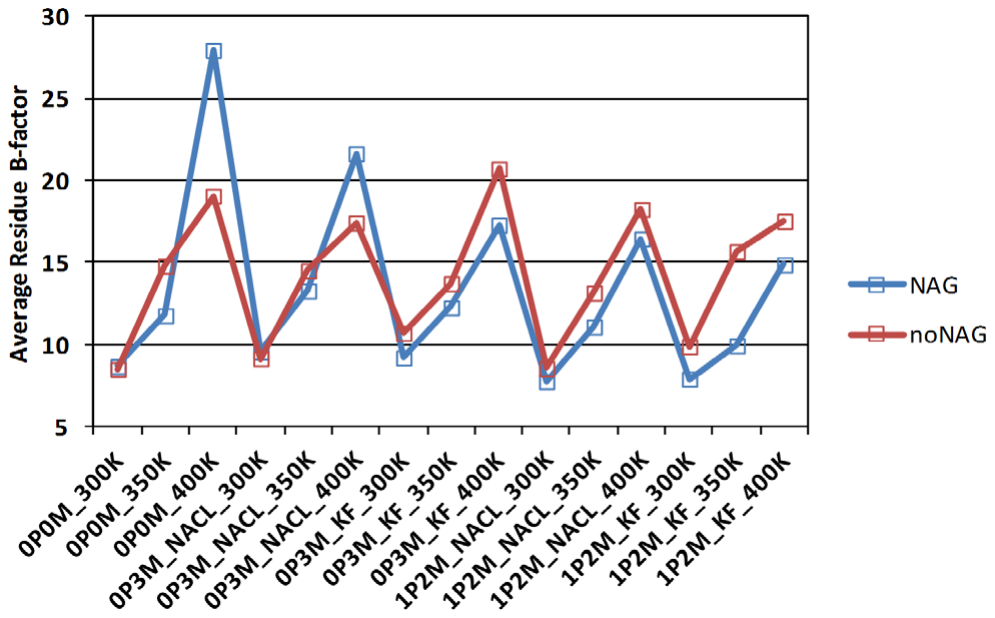
Average B-factor calculated across all 499 residues in each simulation.

The B-factor profiles from the simulations (File S1, Figures S40−S49) show that fluctuations generally increase with temperature as expected from the increase in thermal energy. This increase in fluctuations with temperature can be visualized in a compressed way by the average B-factor of all 499 TvLα residues (Figure 4). The average B-factor is smaller (by up to 2.5 Å^2^) for most simulations of glycosylated protein (blue), except the non-physiological 0 M and the 0.3 M NaCl simulation at 400 K, where disorder is markedly larger: These two cases of larger disorder are associated with loop motions, in particular in the C-terminal part of the protein, to be discussed below. In all other cases where larger motions have not occurred, the glycosylation effect is consistently reducing protein thermal motion across all physiological and near-physiological states. Altogether, the analysis of thermal disorder supports the physical realism of the simulation protocol.

### Secondary Structure Formation from Average Number of Backbone Hydrogen Bonds

**Figure 5:**
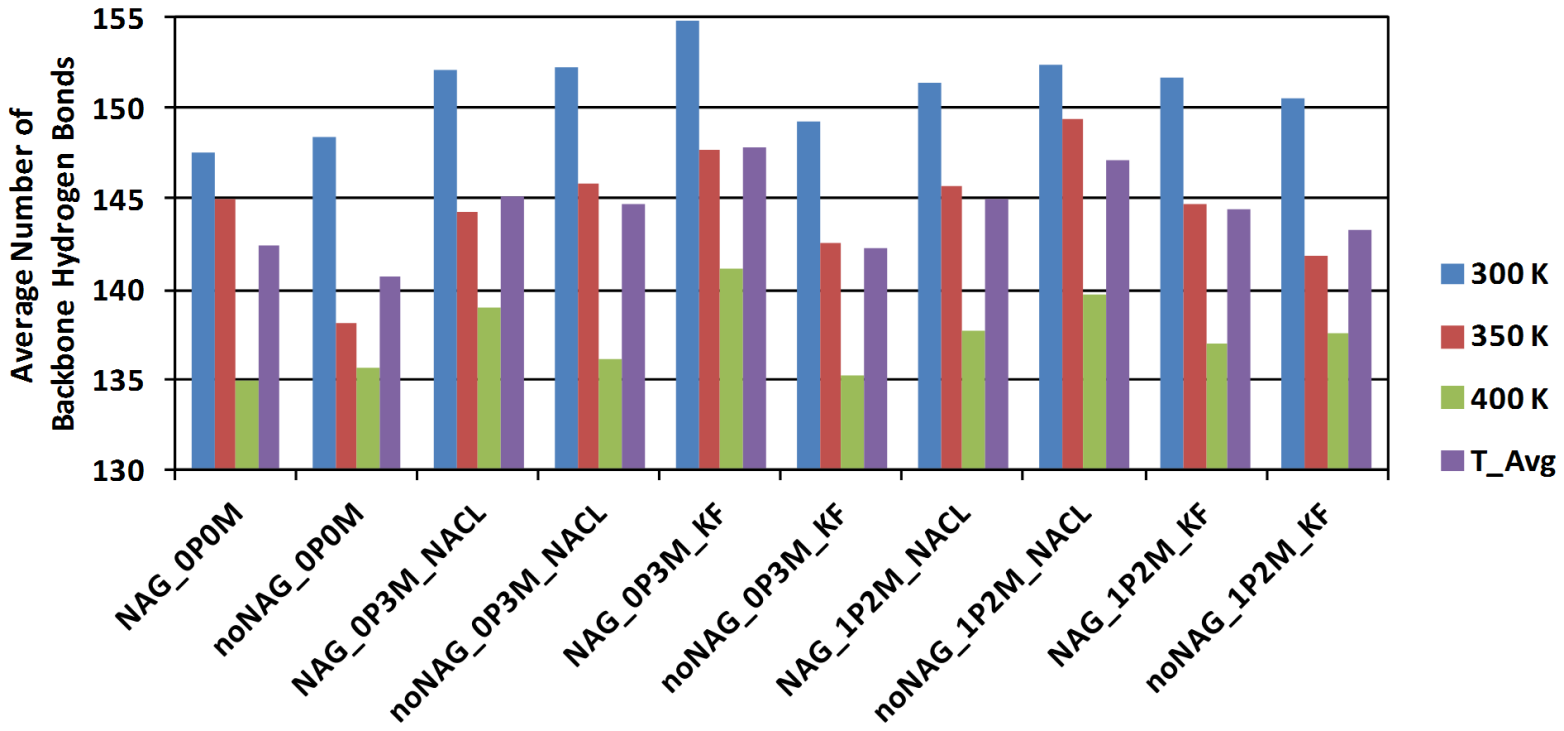
Average number of backbone hydrogen bonds in last 2 ns of each MD trajectory. 300 K, 350 K, 400 K and the temperature average (T_Avg) are shown with bars in blue, red, green, and purple, respectively.

After having compared the simulated structure and dynamics directly against crystal structure data, we now move on to discuss the changes in structure associated with perturbing the protein environment. Figure 5 shows the average number of backbone hydrogen bonds for the last 2 ns of the NVT-simulations, using the hydrogen-bond detection criteria of Jorgensen and co-workers [64].

Figure 5 shows that the number of hydrogen bonds is consistently anti-correlated with temperature: A temperature increase of 50 K typically leads to a loss of ∼7 backbone hydrogen bonds. Thus, the simulations consistently probe the disruption of secondary structure in the laccase as a function of higher T corresponding to a shift of interactions from enthalpy-dominated to entropy-dominated, as the TΔS term is favored at higher T. Such entropy-enthalpy compensation is well-known experimentally [26], [27], [28], [68], [69] but is rarely quantified structurally. The quantification of a loss of ∼7 average hydrogen bonds per 50 K relates only to the chosen criteria [64]. However, the trend (not the gradient) should be consistent regardless of the detection criteria used and thus defines an MD-based proxy of the thermodynamic restructuring of the protein of direct relevance to protein unfolding and stability. We find that heat is dispersed into hydrogen bonds not only of the loosest, more dynamic kind, but also into the structure-preserving secondary structure hydrogen bonds, to be investigated further below.

The largest amount of hydrogen bonds lost at high T occur in salt-free solution, suggesting that absence of salt renders the secondary structure less resistant to temperature. Such an effect of salt, although commonly observed experimentally, has to our knowledge never been identified theoretically, because it requires multiple seed MD-runs at varying ionic strength and T. Furthermore, in the non-glycosylated protein, KF causes substantial disruption of secondary structure both at 0.3 M and 1.2 M KF, with ∼2.5 less hydrogen bonds than corresponding simulations in NaCl, suggesting that glycosylation prevents small anion interactions with secondary structure components. Only these two simulations disrupt secondary-structure almost as much as the 0 M simulations.

Consistent with these observations, overall, [NAG_0P0M_400 K] and [noNAG_0P3M_KF_400 K] display the lowest number of average hydrogen bonds observed (134.9 and 135.2, respectively). As will be discussed later, the large RMSD of the [NAG_0P0M_400 K] simulation is due to partial denucleation at the C-terminal part of the protein, indicating a possible early unfolding pathway at very rough conditions, to be analyzed at the end of this paper.

### Secondary Structure Formation from Persistent Backbone Hydrogen Bonds

**Figure 6:**
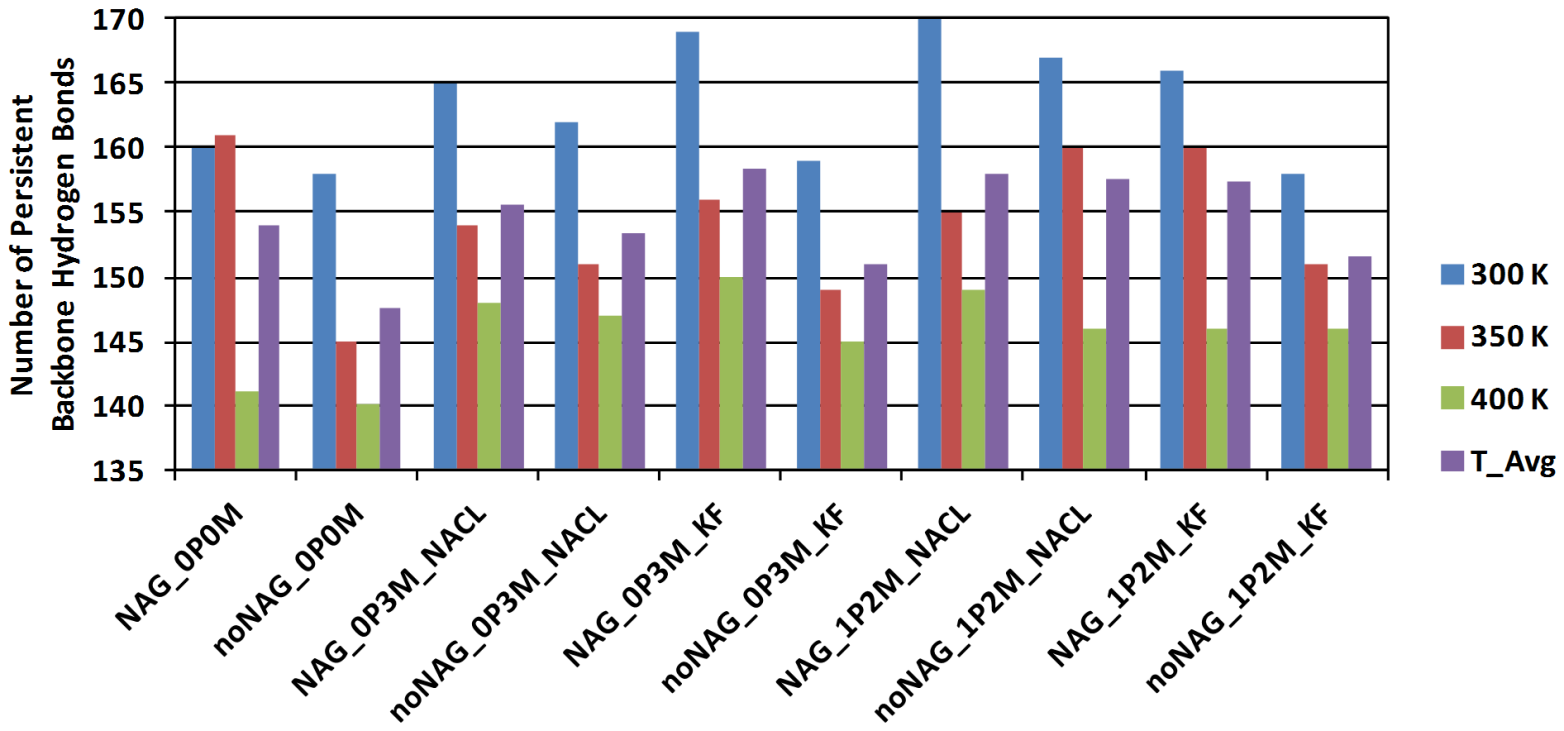
Number of persistent backbone hydrogen bonds in last 2 ns of each MD trajectory. Persistence is given as the percentage of the number of trajectory frames (100) saved for the last 2 ns of the NVT simulations. 300 K, 350 K, 400 K and the temperature average (T_Avg) are shown with bars in blue, red, green, and purple, respectively.

To make the analysis more stringent, Figure 6 shows the number of *persistent* backbone hydrogen bonds per simulation, each computed from 100 snapshots of the equilibrated trajectories. The greater range of counted hydrogen bonds in Figure 6 reflects that the average number of hydrogen bonds at a given time is typically smaller than the number of hydrogen bonds present for more than 50 percent of the total simulation time.

Overall, the trends in persistent hydrogen bonds agree with those for the average number of hydrogen bonds, demonstrating that structurally important persistent backbone hydrogen bonds are consistently influenced by the perturbations. The strongest trend is again seen for temperature, with an average loss of ∼9 persistent hydrogen bonds per 50 K. More hydrogen bonds are lost from 300 K to 350 K than from 350 K to 400 K in most cases, excluding the non-physiological glycosylated 0 M simulation as well as [noNAG_1P2M_NACL] and [NAG_1P2M_KF], which behave similarly. In salt-free solution, the largest number of hydrogen bonds is lost at high T in both the glycosylated and non-glycosylated case, strengthening the notion that the absence of salt renders secondary structure more vulnerable to disruption at high T.

The increased disruption in the non-glycosylated protein at 0.3 M and 1.2 M KF is also found in the more statistically significant persistency analysis: Relative to the corresponding simulations in NaCl, there are ∼2 fewer persistent hydrogen bonds for 0.3 M KF and ∼6 fewer for 1.2 M KF. The effect is mostly seen at 300 K and 350 K. One possible explanation is that small hard anions form persistent enthalpic interactions via strong F–H hydrogen bonds that are entropically unfavorable at high T, consistent with the enthalpy-entropy compensation changes as T increases discussed above.

To substantiate this, we computed the radial distribution functions (RDF) for backbone NH and F^−^ (for KF simulations) and backbone NH and Cl^−^ (for NaCl simulations) (File S1, Figures S34−S39). In addition to an expected thermal broadening of the RDFs as entropy overtakes the enthalpy of the NH–X hydrogen bonds, the absence of NAG increases the number of bound F^−^ but slightly decreases the number of bound Cl^−^. Thus, the RDF for KF confirms a mechanism of small-anion intrusion as a source of secondary structure disruption, partly prevented by glycosylation. The larger Cl^−^ ions interact more weakly with the secondary structure due to weaker N-H–Cl (2.5 Å) than NH–F (∼2 Å) hydrogen bonds, and due to reduced diffusion into the protein. The suggested competition between two identified salt effects, one general and stabilizing by surface-interactions (with Cl^−^ working more to this effect than F^−^), and one destabilizing by intrusion and backbone-hydrogen-bond disruption (with F^−^ being smaller, harder, and thus more intrusive), clearly requires more investigation.

It is generally considered that glycosylation may stabilize extracellular proteins in addition to its protecting of protease cleavage sites [16], and it is experimentally known that smaller halides inhibit laccases more (i.e. F^−^>Cl^−^) with weak correlation to the redox potential [12]. The identified small anion disruption of secondary structure and the role of glycosylation in protecting against this disruption is to our knowledge the first observed molecular mechanism for such a protective, stabilizing effect of glycosylation, which would be significant in an extracellular environment subject to large ionic strengths of small anions. Site-directed mutagenesis towards protecting the protein against small anion intrusion at critical sites (i.e. those contributing most to the NH–F RDF) would be a new, potentially valuable strategy for laboratory evolution of laccases (and possibly other stable industrial proteins).

For the non-glycosylated protein states relative to the glycosylated states, an average loss of ∼4.4 hydrogen bonds across temperature and ionic strength is observed from comparing the average-T purple bar one-by-one across the series. The effect is consistent in 14 of the 15 comparisons. As the notable outlier, the protein unexpectedly loses five persistent hydrogen bonds in 1.2 M NaCl at 350 K upon glycosylation. To be discussed later, NaCl has a stabilizing effect showing up when glycosylation does not prevent anion diffusion into the protein. Absent this case, glycosylation consistently strengthens secondary structure hydrogen bonds, which is a possible explanation for the experimental findings that N-glycosylation confers thermostability to *Trametes versicolor* laccase isoform 3 [70] as also observed for other classes of proteins [71], [72], [73]. Our MD-simulations produce a significant (14 of compared 15 pairs of simulations averaged over 100 snapshots each) secondary-structure-preserving effect of glycosylation and relate it to an intriguing interplay between salt penetration and thermal disruption of secondary structure.

The location of the most important sacrificed hydrogen bonds is analyzed in Tables S6 and S7 in File S1. The most consistently sacrificed hydrogen bonds as a function of T (300 to 350 K, i.e. moving towards T_opt_ where the protein starts to lose activity) occur mainly in exposed loops and in the ends of β-sheets, which is reasonable considering the more exposed and weaker interactions in those positions. The mechanism of thermal denaturation inferred without an actual denucleation event (to be discussed below) thus appears to be β-sheet “unzipping” in the cupredoxin domains. Incidentally, many standard MD protocols neglect post-translational modifications and sometimes even ionic strength, which as shown here significantly affects secondary-structure. Thus, even disregarding deficiencies in force fields and simulation protocols, the physical model alone should clearly be realistic when performing protein simulations.

### Structural Interpretation of Glycosylation Effects

**Figure 7:**
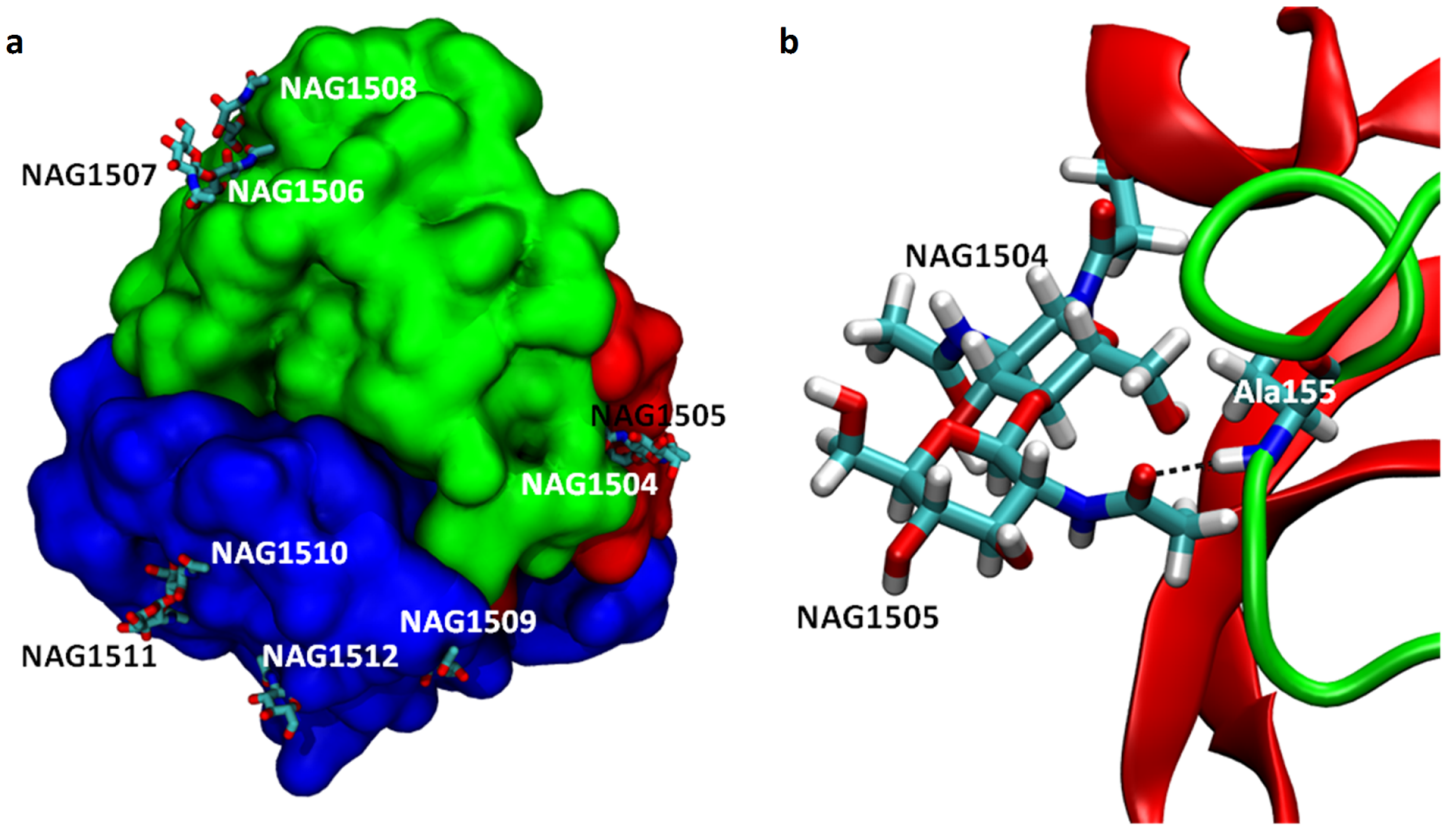
TvLα crystal structure N-glycosylation. (A) Overview: Domains D1, D2, and D3 are colored red, green, and blue, respectively. NAG residues proximal and distal to the protein surface are shown in white and black, respectively. (B) The domain-bridging NAG1504-NAG1505 attached to Asn54 in D1 (red) forms a hydrogen-bond with the backbone nitrogen of Ala155 in D2 (green) in the crystal structure of TvLα.

As described above, in addition to the expected effect of temperature on secondary structure, we have also observed a combined salt and glycosylation effect across simulations. To understand these effects further, The N-acetylglucosamine moieties present in the crystal structure of TvLα have been shown in Figure 7a.

One disaccharide (NAG1504-NAG1505) is located between domains D1 and D2, and could in principle provide stability against domain-domain denucleation (Figure 7b). Such a role has previously been suggested for the glycan located near the cleft between two domains in a laccase from the ascomycete *Thielavia arenaria* [74]. In the crystal structure of TvLα, the NAG1505 hydrogen bonds to the backbone NH of Ala155 in D2 (Figure 7b). This hydrogen bond is transient in the MD simulations, as evident from the persistence of all hydrogen bonds between NAG and amino acid residues (Table 2): The NAG1505-Ala155 hydrogen bond is present for ∼20% of the simulation time except in 0.3 M KF at 350 K where its persistence is 46%. In this case, stabilization by NAG does not appear to result from domain-NAG-domain hydrogen bonds. However, both glycans with direct hydrogen bonds to the protein and glycans that merely adhere to the surface may prevent anions from penetrating into secondary structure elements at higher temperatures (where anion diffusion into the protein will dominate) via steric hindrance.

**Table 2:** Persistence of hydrogen bonds between N-acetylglucosamine and amino acid residues in percent*.

		0.0 M Salt			0.3 M NaCl			1.2 M NaCl			0.3 M KF			1.2 M KF		
NAG site	Interaction	300 K	350 K	400 K	300 K	350 K	400 K	300 K	350 K	400 K	300 K	350 K	400 K	300 K	350 K	400 K
Asn54	NAG1504-Arg22	0	0	4	39	8	8	51	69	69	11	6	9	3	0	19
	NAG1504-Asp23	76	83	62	1	62	27	0	7	31	37	71	46	68	69	75
	NAG1504-Thr56	87	70	21	0	3	46	0	0	45	79	0	32	0	78	0
	NAG1505- Ala155	21	3	13	21	6	1	32	15	0	14	46	21	18	18	11
Asn217	NAG1506-Asp234	91	21	63	86	36	49	83	45	47	87	62	5	86	80	24
Asn251	NAG1508-Asn217	3	22	29	69	15	30	8	22	3	42	44	56	6	33	24
	NAG1508-Asn249	14	10	12	4	13	10	32	28	13	8	9	13	36	14	26
Asn333	NAG1509-Thr335	22	63	12	9	26	27	36	35	68	81	72	0	44	53	17
Asn436	NAG1510-Asn327	0	0	2	48	7	22	4	0	21	0	7	13	0	3	14

* Based on 100 trajectory frames of the last 2 ns of NVT-simulations.

Table 2 shows that local hydrogen bonds between the first (proximal) carbohydrate moiety and nearby residues on the protein surface for NAG1504, NAG1506, NAG1508, NAG1509, and NAG1510 persist for more than 50% in some simulations. This persistency reflects reduced mobility of the proximal carbohydrate moiety and defines the conformation of the carbohydrate on the surface of the protein as previously discussed [71]. NAG1504 is frequently hydrogen bonded to Asp23, and to a lesser degree to Thr56, and Arg22. This implies that the NAG1504-NAG1505 disaccharide maintains its overall crystal structure orientation and bridges D1 and D2 during simulation, and thus may reduce solvent and small anion intrusion into the cleft between D1 and D2.

**Figure 8:**
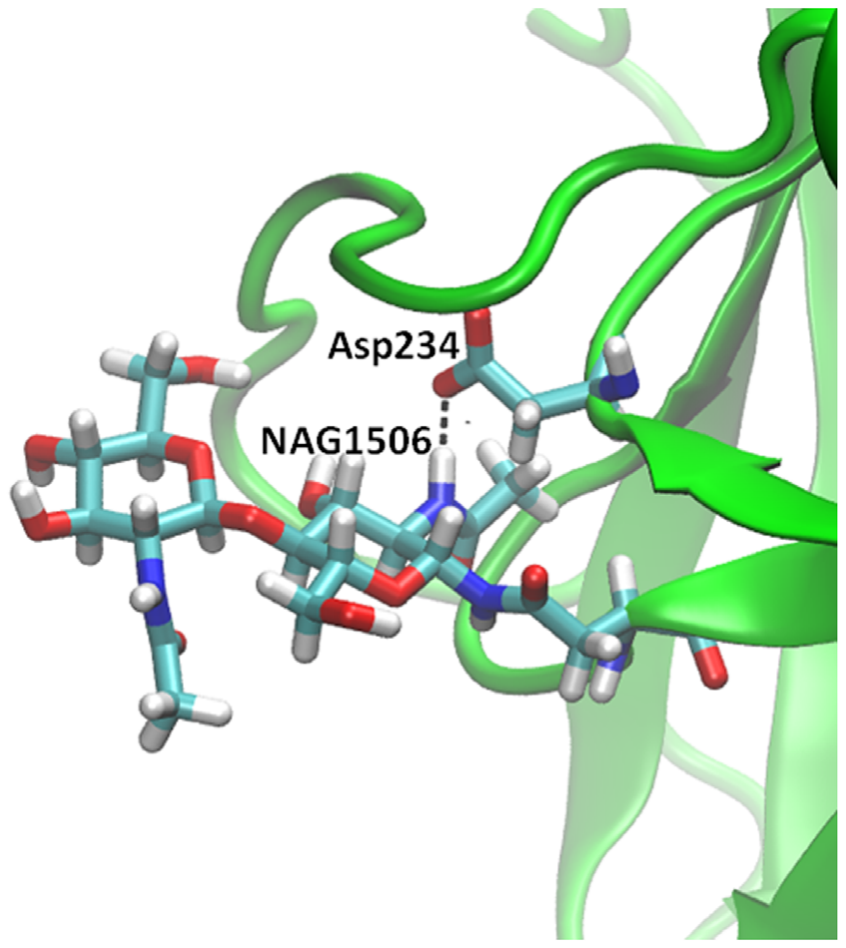
The most persistent hydrogen bond between a N-acetylglucosamine moiety and protein. The hydrogen bond occurs between NAG1506 and Asp234 and persists for 5%–91% of the time in NVT MD simulations, see Table 2. The snapshot was obtained from the simulation in 0.3 M NaCl at 300 K.

The most persistent hydrogen bond across temperature and ionic strength occurs between the proximal half of the NAG1506-NAG1507 disaccharide, NAG1506, and the neighboring Asp234 residue (Figure 8; see data in Table 2). The persistence is 21−91% for all simulations, except 0.3 M KF at 400 K for which the persistence is <10%. Unlike other NAG residues, there is a tendency for these hydrogen bonds to be more persistent at lower temperatures: The average persistence across all simulations is ∼72%, 41%, and 38% at 300 K, 350 K, and 400 K, respectively.

Overall, this analysis suggests that the carbohydrate moieties found on the TvLαsurface have relatively conserved orientations, partially stabilized by hydrogen bonds to the protein surface. This restricts small ion access to inter-domain (D1−D2) clefts, consistent with the reduced NH–F^−^ RDFs with glycosylation (Figures S34−S39 in File S1), and partially explains the enhanced secondary-structure integrity for the glycosylated protein observed in this work.

### Salt Bridges

Solvent-exposed salt-bridges may stabilize some thermophilic and hyperthermophilic proteins, in particular between domains and secondary structure elements [75]. Salt-bridges are likely to be more stabilizing at high temperatures, as the desolvation cost for fixing an ion pair decreases with temperature [76].

For the laccase studied here, some salt bridges, Asp128-Lys40, Asp214-Arg260, Asp224-Arg423, and Asp424-Arg243, are conserved in all simulations for substantially more than 50% of the equilibrated simulation time, see Tables S2− S5 in File S1. These long-lived interactions bridge distant parts of the enzyme and may thus be identified as affecting structural integrity. The average distance and persistence of these salt bridges is independent on temperature, ionic strength, or glycosylation status, suggesting that they are among the strongest protein interactions and thus late to be sacrificed upon denaturation.

Some salt bridges are present for more than 50% of the time in certain simulations but not others, and some salt bridges are formed and destroyed irrespectively of temperature. However, in addition to identifying the most persistent salt bridges (above), it is also relevant to identify those salt bridges that are persistent at physiological T but sacrificed at high T (400 K), as these are the likely interactions to be broken during thermal denaturation. The most notable such interaction is Asp486-Lys482, which is sacrificed in three simulations from 300 K to 400 K and not formed in any of these cases (Tables S2− S5 in File S1) (several other salt bridges are lost in one simulation at 400 K which is not significant). Asp486-Lys482 is located in the N-terminal part of the C-terminal α-helix, which may be important for initial unfolding according to the partial denucleation in zero ionic strength at 400 K to be discussed below. Thus, stabilization of this salt bridge, e.g. by introduction of hydrophobic residues to reduce electrostatic screening of the interaction, may assist in preserving the nucleated state of the C-terminal helix.

### Large Backbone Fluctuations

Although fluctuations in most parts of the TvLα sequence are comparable between the crystal structure and MD simulations (Figure 3; disregarding the crystal structure offset for B-factors), certain residues are associated with strongly amplified fluctuations in some simulations, reflecting structural rearrangements in response to environmental changes. To understand these fluctuations, the residue segments displaying the largest fluctuations are shown in Table 3 together with the B-factor for the most fluctuating amino acid in each segment. In agreement with previously determined relationships between environmental effects and residual fluctuations for 184 proteins [77], the most prominent fluctuations in TvLα occur in turns, coils, and helices exposed to solvent. Furthermore, the segments that are involved in all prominent fluctuations contain a high percentage of residues categorized [77] as “highly fluctuating” (average 57%) and “moderately fluctuating” (average 29%).

**Table 3:** Maximum B-factors for highly disordered segments of TvLα.

Simulation	T(K)	Residues							
		153–166	177–183	265–275	308–312	358–364	409–414	430–436	470–499
NAG_0P0M	300	22	41	35	15	93	18	25	29
	350	35	70	99	25	26	21	72	31
	400	89	129	126	39	49	37	169	1142
noNAG_0P0M	300	26	17	19	46	17	14	24	36
	350	77	92	23	98	28	30	66	154
	400	82	25	28	161	42	54	58	196
NAG_0P3M_NACL	300	23	69	20	35	21	23	52	52
	350	44	98	42	161	22	29	113	56
	400	45	201	59	60	42	108	386	120
noNAG_0P3M_NACL	300	48	28	16	24	20	17	41	39
	350	78	26	49	158	22	49	101	32
	400	48	147	25	84	80	39	114	55
NAG_0P3M_KF	300	27	14	15	50	23	16	19	35
	350	40	19	17	55	24	30	96	136
	400	124	46	37	43	29	37	166	54
noNAG_0P3M_KF	300	21	41	104	51	17	30	139	29
	350	26	33	79	199	23	27	63	33
	400	61	193	97	139	36	68	114	102
NAG_1P2M_NACL	300	17	29	22	32	19	17	44	25
	350	28	37	28	73	32	25	99	42
	400	74	56	31	92	33	36	179	47
noNAG_1P2M_NACL	300	21	14	24	70	16	19	45	37
	350	44	18	15	54	27	25	74	144
	400	63	99	133	81	25	69	84	245
NAG_1P2M_KF	300	13	18	12	24	20	13	48	27
	350	20	21	16	46	24	24	70	28
	400	28	35	22	50	86	41	83	54
noNAG_1P2M_KF	300	39	25	15	40	24	15	52	32
	350	84	71	52	191	30	23	115	58
	400	225	34	88	70	33	53	70	43

Fluctuations in the loop comprised of residues 153–166 (His, Thr, Ala, Ala, Arg, Leu, Gly, Pro, Arg, Phe, Pro, Leu, Gly, Ala) near the T1 site generally increase monotonously with temperature, with typical maximum B-factors of ∼80–90 Å^2^ at 400 K. The largest deviation occurs in the [noNAG_1P2M_KF_400 K] simulation, with a maximum B-factor of 225 Å^2^. Since backbone hydrogen bond counts for this simulation resemble several other 400 K simulations, the strong 153–166 fluctuation is not in itself further disrupting secondary structure. The loops’ exposed location and interaction with the distal half of the NAG1504-NAG1505 disaccharide instead suggests, as discussed above, that this disaccharide dampens thermal motion and excludes F^−^, which diffuses more easily at high temperature (400 K) and high ionic strength and without the protecting glycan. Arg157 and Arg161 probably participate in tethering the loop to the protein surface.

Residues 177–183 (Ser, Ala, Ser, Thr, Pro, Thr, Ala) comprise two consecutive type I β-turns: (Ser177-Ala178-Ser179-Thr180) and (Thr180-Pro181-Thr182-Ala183). The four largest fluctuations of 201 Å^2^, 193 Å^2^, 147 Å^2^, and 129 Å^2^ occur for [NAG_0P3M_NACL_400 K], [noNAG_0P3M_KF_400 K], [noNAG_0P3M_NACL_400 K], and [NAG_0P0M_400 K], respectively. As it is remote from copper- and N-glycosylation sites, it constitutes a potential target for stabilization by turn-sequence optimization. The statistical preferences of amino acid positions in β-turns [25] suggest that mutations such as A178P and A183G may potentially increase laccase stability.

Residues 265–275 fluctuate maximally in the [noNAG_1P2M_NACL_400 K] and [NAG_0P0M_400 K] simulations (133 Å^2^ and 126 Å^2^, respectively). Most other simulations have fluctuations of 50 Å^2^ or below. Thus fluctuations in this area are principally triggered by rough conditions. The Type II and type IV turns corresponding to residues (270–273) and (265–268), respectively, may thus be suggested as targets for a site-directed mutagenesis strategy.

**Figure 9:**
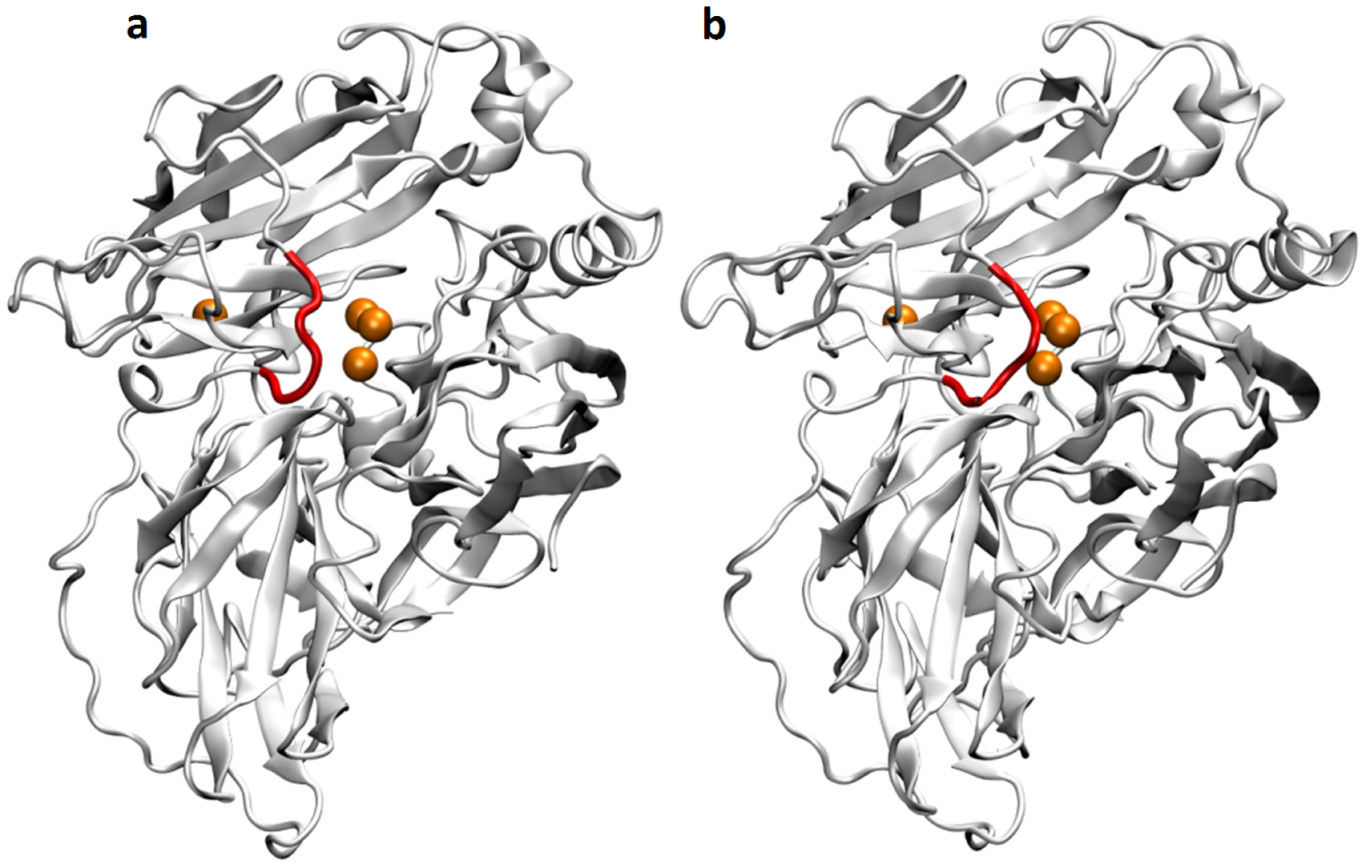
Movement of the turn 308–312. The turn comprised by residues 308–312 (red) undergoes a hinge-like movement in the glycosylated 0.3 M NaCl simulation at 350 K simulation (B) but not at 300 K (A).

A structural change revealed from the fluctuation plots occurs in residues 308–312 (Leu, Ala, Arg, Met, Pro), comprising a turn that undergoes a hinge-like movement in the physiological, glycosylated 0.3 M NaCl simulation at 350 K (Figure 9b) but not at 300 K (Figure 9a), resulting in partial occlusion of the water exit channel at the T2 site. The maximum fluctuations occur at 350 K in five simulations, close to *T*_opt_ = 350 K for ABTS oxidation for the closely related TvLα [5]. This finding could be coincidental or could reflect a mechanistically relevant correlation with optimal enzyme function at 350 K, which could be investigated by site-directed mutagenesis in this turn.

**Figure 10:**
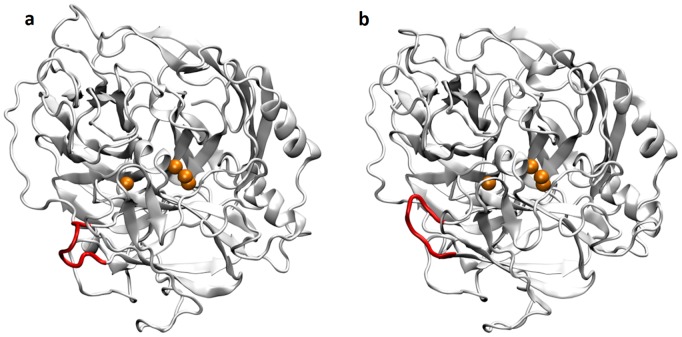
Movement of the turn 430–436. (A) first and (B) last frame from the glycosylated 0.3 M NaCl simulation at 400 K illustrating the movement of the turn 430–436 (in red). Copper atoms are shown as orange spheres.

Residues 430–436 (Thr, Pro, Ala, Ala, Gly, Asp Asn) show large fluctuations in all simulations. Although Asn436 is glycosylated with NAG1510-NAG1511, the fluctuations do not correlate with glycosylation, suggesting that NAG1510-NAG1511 does not modulate protein stability by direct interactions, supported by the absence of hydrogen bonds between protein and NAG1510-NAG1511 in most simulations (Table 2). The largest fluctuation (386 Å^2^) is found for [NAG_0P3M_NACL_400 K]. Reorganization of this backbone hydrogen bonding network coincides with a hinge motion (Figure 10a vs. Figure 10b). This rearrangement does not occur in e.g. the physiologically relevant [NAG_0P3M_NACL_300 K] simulation, where the peak B-factor of 52 Å^2^ is associated with fluctuations around the initial state (Figure 10a). Therefore, this turn should also be a relevant target in laboratory evolution of enhanced fungal laccase stability.

### Electrostatic Energy Analysis of Secondary-Structure Disruption

**Figure 11:**
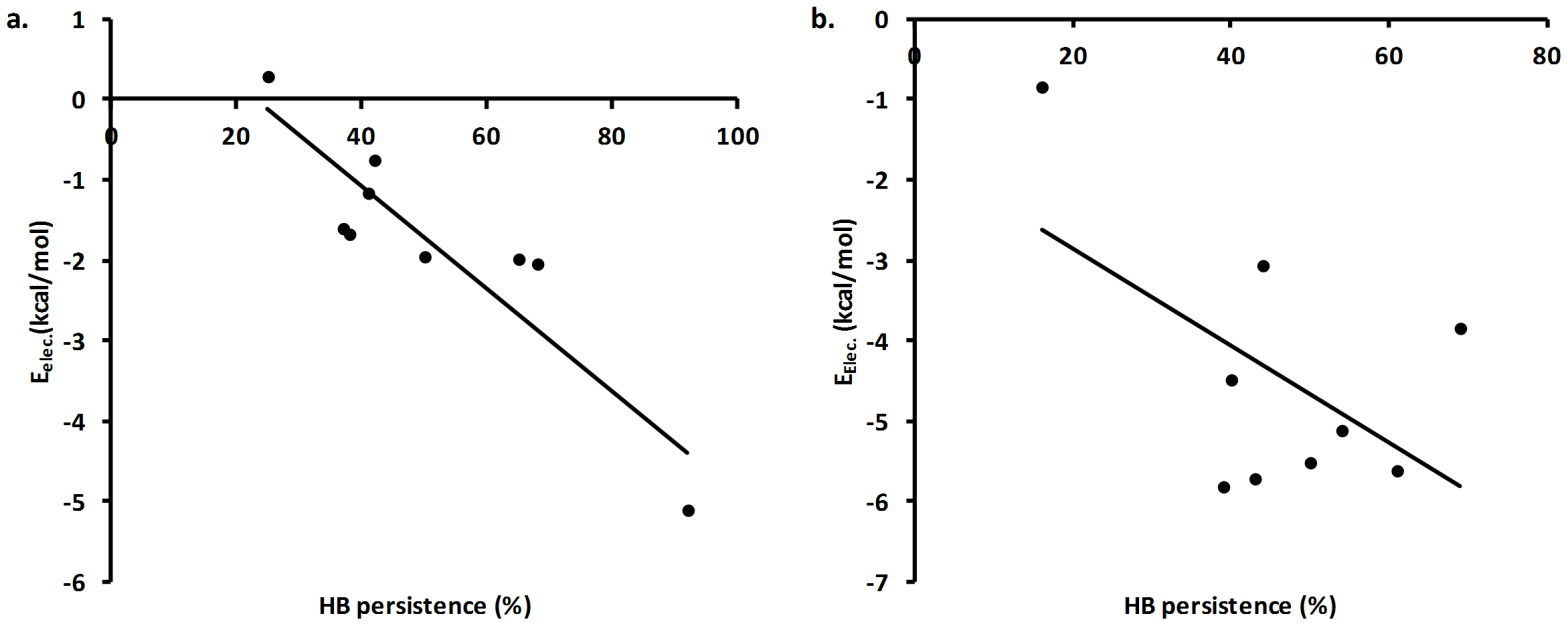
Loss of enthalpy correlates with secondary-structure loss at high T. Correlation between backbone hydrogen bond persistence (%) and electrostatic interaction energy (kcal/mol) for (a) residue pairs in structured parts of the protein and (b) residue pairs in loosely structured parts of the protein.

Six backbone-backbone hydrogen bonds in the laccase (Asp492−Cys488, Gln45−Pro4, Thr383−Leu326, Ala134−Asp131, Asn304−Ile301, and Ser370−Pro367) were found to be consistently sacrificed at 350 K in both physiological ionic strengths of 0.3 and 1.2 M NaCl (marked in bold in Tables S6 and S7 in File S1). The persistence averaged for these six hydrogen bond pairs (Table S8 in File S1) was compared with the averaged electrostatic interaction energy between the residues (Figure S51 in File S1). This energy and the hydrogen bond loss are correlated, as seen in Figure 11: As the average backbone hydrogen bond persistence is reduced with temperature, the average residual electrostatic interaction energy becomes less negative. Both proxies are thus applicable for monitoring the secondary structure disruption in the proteins.

As seen in Figure 11, the correlation between hydrogen-bond persistence and electrostatic interaction decreases (*r* = −0.44, *r* ^2^ = 0.19) when all six interactions for all simulations are considered simultaneously but the correlation is very high (*r* = −0.90, *r* ^2^ = 0.81) when only the three interactions that are strict secondary structure (Asp492−Cys488, Gln45−Pro4, Thr383−Leu326) are considered, and this is consistent across the simulations (the correlation plot for the total set of interactions can be seen in File S1, Figure S52 based on the persistence and electrostatic energies given in Table S8 in File S1).

Importantly, both backbone hydrogen bond persistence and simulation-averaged electrostatic energies of the involved residues vary in a predictable and physically reasonable way with temperature. This shows that hydrogen bond sacrifice associated with secondary structure loss is directly related to energy/enthalpy increase in these critical laccase sites. Both of these observations indicate the loss of enthalpy of folding as temperature increases and favors entropy over enthalpy.

### Observed Partial Denucleation of TvLα under Rough Conditions

**Figure 12:**
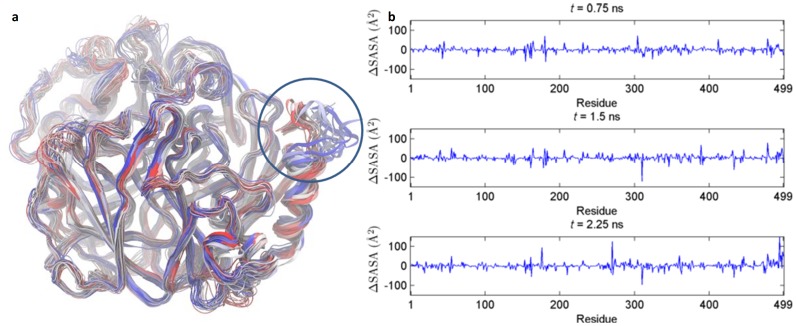
Increased C-terminal flexibility in zero ionic background at 400 K. (A) Multiple frame overlay sampled every 100 ps showing the increased flexibility at the C-terminal end of TvLα in the 3 ns NVT simulation of the glycosylated protein at 400 K and zero ionic background ([NAG_0P0M_400 K]). The color shifts from red to blue with the progression of time. (B) Time-development of the residue-resolved SASA for the same simulation. The SASA per residue at *t = *0 has been subtracted from each plot, yielding the ΔSASA at *t* = 0.75, 1.5, and 2.25 ns, respectively.

Overall, the largest local fluctuations (maximum B-factor = 1142 Å^2^ in the 470–499 segment) and structural changes occur in the simulation of the glycosylated protein at 400 K and zero ionic background ([NAG_0P0M_400 K]). The hydrogen bonds stabilizing the C-terminal in the crystal structure are disrupted, markedly increasing flexibility. Distortions of backbone dihedrals cause the C-terminal to denucleate partly over time, as illustrated in Figure 12a using an overlay of MD frames. This mode increases solvent accessibility as noted from the time-development of the SASA for each residue (Figure 12b). To confirm the susceptibility of the C-terminus, we extended the NVT simulations at 400 K in 0, 0.3, and 1.2 M NaCl with and without glycosylation a further 10 ns each. Comparison of the MD snapshots at 10 ns (Figure S50 in File S1) again showed dislodgement of the C-terminal in both the salt-free simulations. In contrast, the simulations in 0.3 M and 1.2 M NaCl conserved the C-terminal conformation. These findings suggest that the C-terminal of TvLαis the most sensitive secondary-structure element, partly denucleating at high T and low IS. It remains to be investigated if it is involved in early unfolding of the entire enzyme. The C-terminal unwinding may be limited by the Cys488-Cys85 bond which tethers the C-terminal α-helix (residues 481−499) to the surface of the enzyme. Previous studies have demonstrated that modifications of the C-terminal can impair function in a 99% homologous TvLα isoform [78]. The denucleation of the same residues observed in this study might possibly explain this observation.

The C-terminal denucleation and solvent exposure in the 400 K simulation is accompanied by increasing electrostatic interactions between water and the helix as seen from an electrostatic energy analysis of the helix-water interactions (File S1, Figure S53). A complementary decrease in the electrostatic interaction between the helix and the rest of the protein is also observed, and these changes are not observed for the 300 K simulation, in agreement with the preservation of C-terminal structure in that trajectory. The partial C-terminal denucleation and the partial unzipping of β-sheets are thus both consistent with electrostatic energy analysis pointing towards enthalpy increase. Thus, in addition to backbone RMSD normally applied, analysis of electrostatic interactions and persistent hydrogen bonds between key structural elements provide important insight into conformational changes occurring in laccases under stressed conditions of potential relevance for optimization strategies in the laboratory.

### Conclusions

In this work, molecular dynamics simulations were carried out for a well-studied fungal laccase (TvLα) with and without glycosylation, in variable ionic strengths, and at 300, 350, and 400 K, to understand in a more systematic way than normally done how environmental properties and glycosylation status affect the structure and dynamics of large proteins.

The overall global conformation of TvLα was conserved in all simulations as evidenced by the median backbone RMSD of 0.92 Å relative to the crystal structure, and both structure and disorder (B-factors) were in good residual agreement between crystal structure and simulations for the most physiologically relevant conditions, confirming the realism of the simulations. From these reference simulations, altered conditions then led to significant changes within the protein as described below:

While the protein size and compactness (radius of gyration, RMSD from a physiological reference state, and solvent-accessible surface) generally varied weakly and unsystematically across simulations, the backbone-backbone hydrogen bond count was identified as a key, sensitive indicator of secondary-structure integrity under the perturbations investigated. Backbone hydrogen bonds decreased significantly with temperature, reflecting entropy-enthalpy compensation: Overall, ∼9 persistent backbone hydrogen bonds were lost per 50 degree temperature increment using a 3.5 Å cutoff. Ionic strength (0.3 M or 1.2 M) was associated with a larger number of persistent hydrogen bonds, but the higher ionic strength (1.2 M) was not associated with more hydrogen bonds than 0.3 M, possibly suggesting an “IS_opt_” in this range for the laccase. On average, moderate ionic strength added ∼3.3 and ∼5.8 persistent hydrogen bonds for glycosylated and non-glycosylated protein, respectively.

Both the average number in each simulation and the number of conserved hydrogen bonds across simulations increased consistently with glycosylation: Averaging across different temperature and ionic strength simulations ∼2.2 average and ∼4.4 persistent hydrogen bonds were gained by glycosylation. This effect was related to an intriguing interplay between salt penetration and temperature disorder disrupting secondary structure. For the non-glycosylated protein, simulations in a KF background produced ∼2 and ∼6 fewer persistent hydrogen bonds at 0.3 M and 1.2 M, respectively, compared to the corresponding simulations in a NaCl background. F^−^ interrupts more the laccase secondary structure by forming strong hydrogen bonds to amide hydrogens, whereas Cl^−^ forms weaker bonds and is less intrusive, probably explaining the experimental observation of the F^−^>Cl^−^ destabilization effect on laccases [12]. Site-directed mutagenesis protecting the protein against small anion intrusion at critical sites would be a new, potentially valuable strategy for laboratory evolution of laccases. However, the two identified salt effects, one general and stabilizing by surface-interactions (e.g. Cl^−^), and one destabilizing by intrusion (with harder F^−^ being more intrusive), clearly requires more investigation.

A number of salt bridges were identified, and the persistence of four of them (Asp128-Lys40, Asp214-Arg260, Asp224-Arg423, Asp424-Arg243) across all temperatures (300–400 K) suggested their role in maintaining structural integrity of TvL, probably being strong and thus late to break during unfolding. On the other hand, salt bridge Asp486-Lys482 is destroyed in three simulations from 300 to 400 K and is thus likely to be weaker and early on the unfolding coordinate, rendering it a more critical target for stabilization by mutagenesis. It is located at the top of the C-terminal helix, which was observed to partially denucleate in our salt-free 400 K simulation (it also showed the largest local fluctuations of all simulations in the glycosylated simulations at 400 K without an ionic background). While F^−^ destabilizes the protein, NaCl was found to protect the C-terminal from denucleation, also correlating with a positive correlation between NaCl presence and overall secondary structure integrity.

As expected, the strongest fluctuations were consistently found at solvent-exposed loops and turns. The most commonly observed loss of secondary structure (i.e. backbone hydrogen bonds) occurred in exposed loops and at the ends of distinct β-sheets (Tables S6, S7 in File S1). A consistent observation was the sacrifice of the three structured hydrogen bonds Asp492−Cys488, Gln45−Pro4, and Thr383−Leu326, correlating strongly with loss of associated electrostatic interaction energy at higher T. Optimization of these critical residues is suggested as part of a strategy for producing highly stable TvLα mutants.

More generally, the work shows that multi-seed, variable environment molecular dynamics can be used to consistently probe critical segments of proteins to understand destabilization mechanisms and explain experimental observations of e.g. glycosylation and salt effects. Several experimental observations regarding laccase stability have been explained in this work and correlated to specific segments of the protein, of potential value for site-directed mutagenesis towards optimizing the stability of this important enzyme.

## Supporting Information

File S1 **Combined supporting information file, containing: Figure S1:** RMSD time series for 10 ns NPT equilibration of glycosylated proteins (NAG) in (a) zero ionic strength, (b) 0.3 M NaCl, (c) 0.3 M KF, (d) 1.2 M NaCl, and (e) 1.2 M KF ionic backgrounds. **Figure S2:** RMSD time series for 10 ns NPT equilibration of proteins without glycosylation (noNAG) in (a) zero ionic strength, (b) 0.3 M NaCl, (c) 0.3 M KF, (d) 1.2 M NaCl, and, (e) 1.2 M KF ionic backgrounds. **Figure S3:** Backbone RMSD time series for 3 ns NVT simulations in 0.3 M NaCl background extended to 20 ns. (a) 300 K, without glycosylation. (b) 400 K, with glycosylation. (c) 400 K, without glycosylation. The curves include RMSD for the initial 3 ns simulations. **Figures S4−S33:** Time series of studied properties from 3 ns NVT simulations. a) SASA, b) Backbone RMSD, c) Radius of Gyration, d) Backbone hydrogen bonds. **Figures S34−S39:** Radial Distribution Functions from the last 3 ns NVT MD for backbone amide-H and halide anions in 0.3 M salt background. **Figures S40−S49:** B-factor plots for 3 ns NVT MD simulations. **Figure S50:** Last snapshots from extended (10 ns) NVT simulations at 400 K of TvLαwith and without glycosylation in NaCl backgrounds of 0 M, 0.3 M, and 1.2 M. **Figure S51:** Backbone hydrogen bond persistence (a) and electrostatic interaction (b) between the involved residues, averaged across the labile hydrogen bond pairs marked in Table S6 and Table S7. Standard deviations are indicated with error-bars. **Figure S52:** Correlation between backbone hydrogen bond persistence (%) and electrostatic energy (kcal/mol) evaluated between the entire residues for (a) residue pairs in structured parts of the protein (red in Table S8), (b) residue pairs in loosely structured parts of the protein (green in Table S8), and (c) residue pairs in both structured and unstructured parts of the protein. **Figure S53:** Electrostatic analysis of the C-terminal unfolding observed for glycosylated TvL in zero ionic background at 400 K (b, d, f), but not at 300 K (a, c, e). The time series show electrostatic interactions between three groups: “water” consisting of all TIP3P molecules, “helix” consisting of the last C-terminal residues 489–499, and “protein_nohelix” consisting of the remainder of the protein. **Table S1:** Statistics calculated from the last 3 ns NVT MD simulations: Average, Standard Deviation, Minimum and Maximum Values for SASA, Rg, and backbone RMSD. **Tables S2−S5:** Salt bridges found in the last 3 ns NVT MD simulations and their persistence (Cons. %) in percentage of 100 frames sampled from 2 last ns of simulations. **Table S6:** Loss of persistent hydrogen bonds for glycosylated TvL in 0.3 M NaCl due to the 300 K to 350 K temperature increase. Hydrogen bonds lost in both the 0.3 M and 1.2 M NaCl background are indicated in bold. **Table S7:** Loss of persistent hydrogen bonds for glycosylated TvL in 1.2 M NaCl due to the 300 K–350 K temperature increase. Hydrogen bonds lost in both the 0.3 M and 1.2 M NaCl background are indicated in bold. **Table S8:** Simulation averaged electrostatic interaction energy between the residue pairs listed in bold in Table S6 and S7 and persistence of backbone hydrogen bonds for the same residues. The analysis was carried out on the last 2 ns of the 300 K, 350 K, and 400 K simulations of glycosylated TvL in 0.3 M NaCl. Hydrogen bonds in structured and loop regions are indicated in red and green, respectively. **Table S9:** Persistent backbone hydrogen bonds (HB) calculated for the 500 MD snapshots between t = 10 ns and t = 20 ns in extended NVT simulations.
(PDF)
