# Supplementary material for: Stability Mechanisms of a Thermophilic Laccase Probed by Molecular Dynamics
Source: PLoS One. 2013 Apr 29;8(4):e61985. doi: 10.1371/journal.pone.0061985 (PMC3639223; doi:10.1371/journal.pone.0061985)
Supplement: File S1 — Combined supporting information file, containing: Figure S1: RMSD time series for 10 ns NPT equilibration of glycosylated proteins (NAG) in (a) zero ionic strength, (b) 0.3 M NaCl, (c) 0.3 M KF, (d) 1.2 M NaCl, and (e) 1.2 M KF ionic backgrounds. Figure S2: RMSD time series for 10 ns NPT equilibration of proteins without glycosylation (noNAG) in (a) zero ionic strength, (b) 0.3 M NaCl, (c) 0.3 M KF, (d) 1.2 M NaCl, and, (e) 1.2 M KF ionic backgrounds. Figure S3: Backbone RMSD time series for 3 ns NVT simulations in 0.3 M NaCl background extended to 20 ns. (a) 300 K, without glycosylation. (b) 400 K, with glycosylation. (c) 400 K, without glycosylation. The curves include RMSD for the initial 3 ns simulations. Figures S4−S33: Time series of studied properties from 3 ns NVT simulations. a) SASA, b) Backbone RMSD, c) Radius of Gyration, d) Backbone hydrogen bonds. Figures S34−S39: Radial Distribution Functions from the last 3 ns NVT MD for backbone amide-H and halide anions in 0.3 M salt background. Figures S40−S49: B-factor plots for 3 ns NVT MD simulations. Figure S50: Last snapshots from extended (10 ns) NVT simulations at 400 K of TvLαwith and without glycosylation in NaCl backgrounds of 0 M, 0.3 M, and 1.2 M. Figure S51: Backbone hydrogen bond persistence (a) and electrostatic interaction (b) between the involved residues, averaged across the labile hydrogen bond pairs marked in Table S6 and Table S7. Standard deviations are indicated with error-bars. Figure S52: Correlation between backbone hydrogen bond persistence (%) and electrostatic energy (kcal/mol) evaluated between the entire residues for (a) residue pairs in structured parts of the protein (red in Table S8), (b) residue pairs in loosely structured parts of the protein (green in Table S8), and (c) residue pairs in both structured and unstructured parts of the protein. Figure S53: Electrostatic analysis of the C-terminal unfolding observed for glycosylated TvL in zero ionic background at 400 K (b, d, f), b [file pone.0061985.s001.pdf]

SUPPORTING INFORMATION

Stability Mechanisms of a Thermophilic Laccase Probed by Molecular Dynamics

*Niels J. Christensen and Kasper P. Kepp**

Department of Chemistry, Technical University of Denmark, Kongens Lyngby, Denmark

* Corresponding author E-mail: **kpj@kemi.dtu.dk**

RMSD time series for 10 ns NPT equilibrations

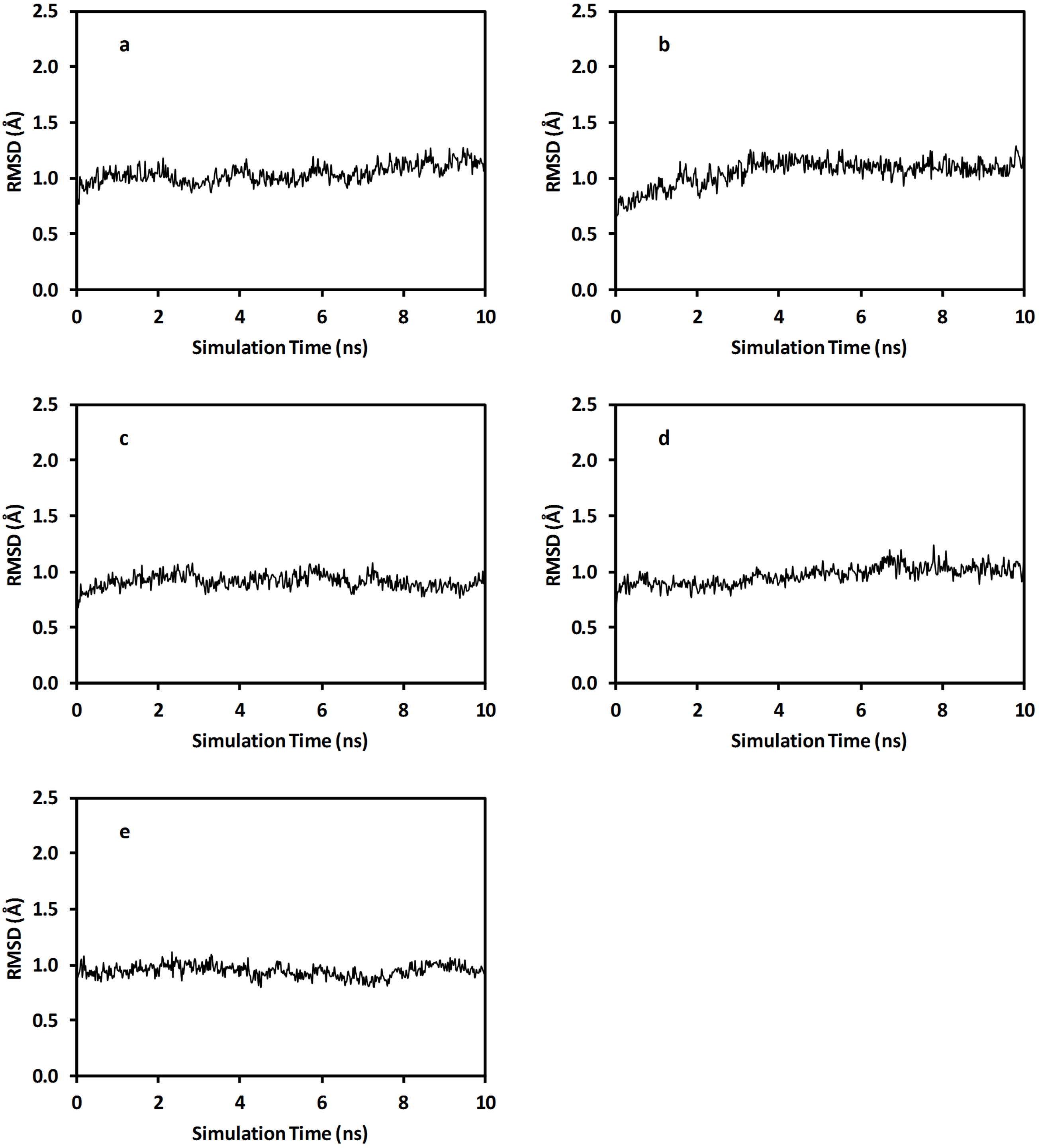

Figure S1. RMSD time series for 10 ns NPT equilibration of glycosylated proteins (NAG) in (**a**) zero ionic strength, (**b**) 0.3 M NaCl, (**c**) 0.3 M KF, (**d**) 1.2 M NaCl, and (**e**) 1.2 M KF ionic backgrounds.

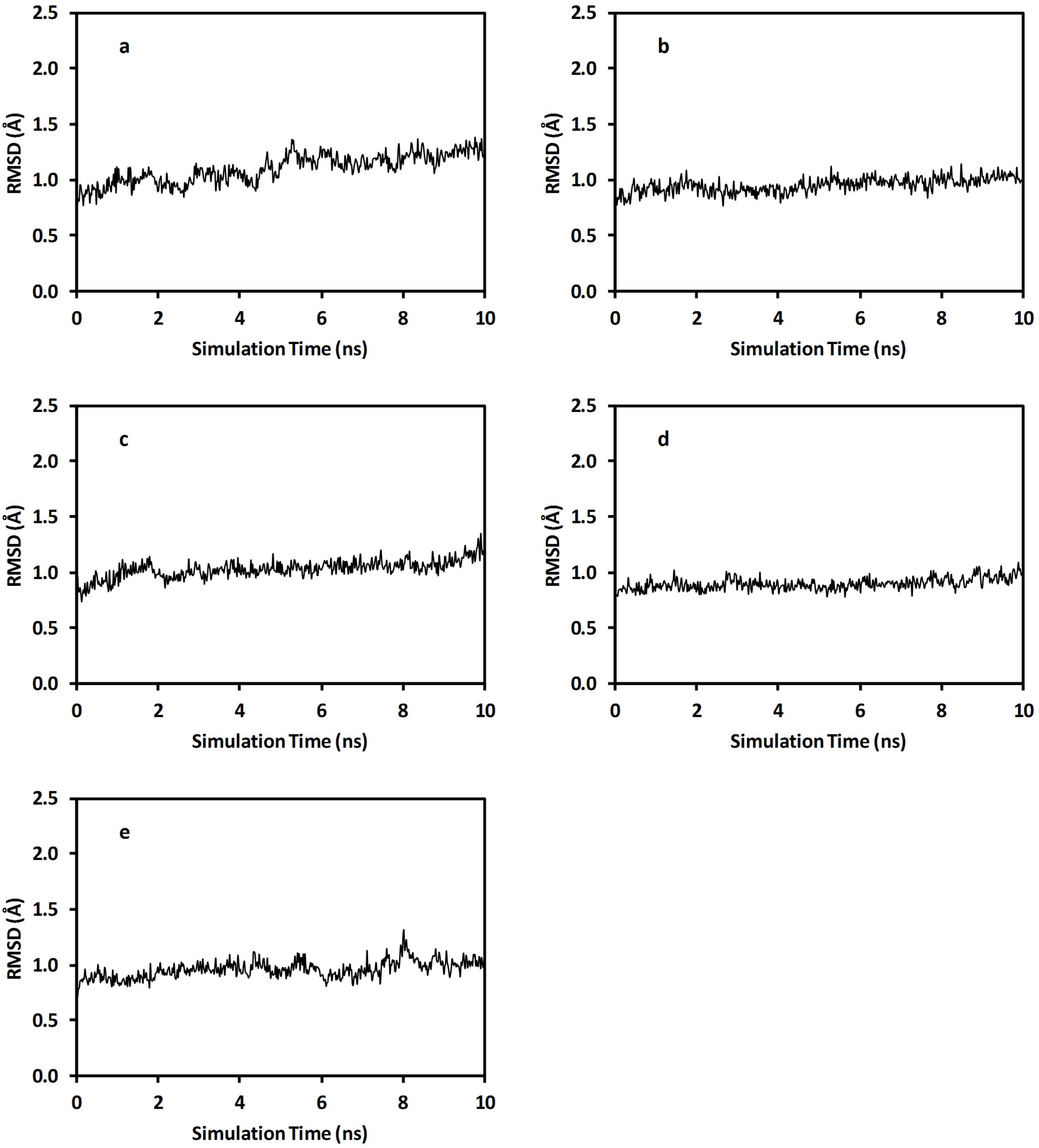

Figure S2. RMSD time series for 10 ns NPT equilibration of proteins without glycosylation (noNAG) in (**a**) zero ionic strength, (**b**) 0.3 M NaCl, (**c**) 0.3 M KF, (**d**) 1.2 M NaCl, and, (**e**) 1.2 M KF ionic backgrounds.

Extended Reference Simulations

As discussed in the main text, the 3 ns NVT reference simulation (glycosylated protein, 0.3 M NaCl, 300 K) was extended by 20 ns in four additional simulations with different seeds. Similarly, we extended the following simulations to 20 ns: TvL without glycosylation in 0.3 M NaCl at 300 K, TvL with glycosylation in 0.3 M NaCl at 400 K, and TvL without glycosylation in 0.3 M NaCl at 400 K. The backbone RMSD curves for these simulations are shown in Figure S3a, S3b, and S3c, respectively.

The RMSD curve for the non-glycosylated protein at 300 K in 0.3 M NaCl background (Figure S3a) is well-behaved throughout the entire 20 ns simulation. The RMSD curves for the 400 K simulations of the glycosylated protein (Figure S3c) and non-glycosylated protein (Figure S3d) both shows substantial increases after ~3 - 5 ns in agreement with the magnitude of the temperature perturbation.

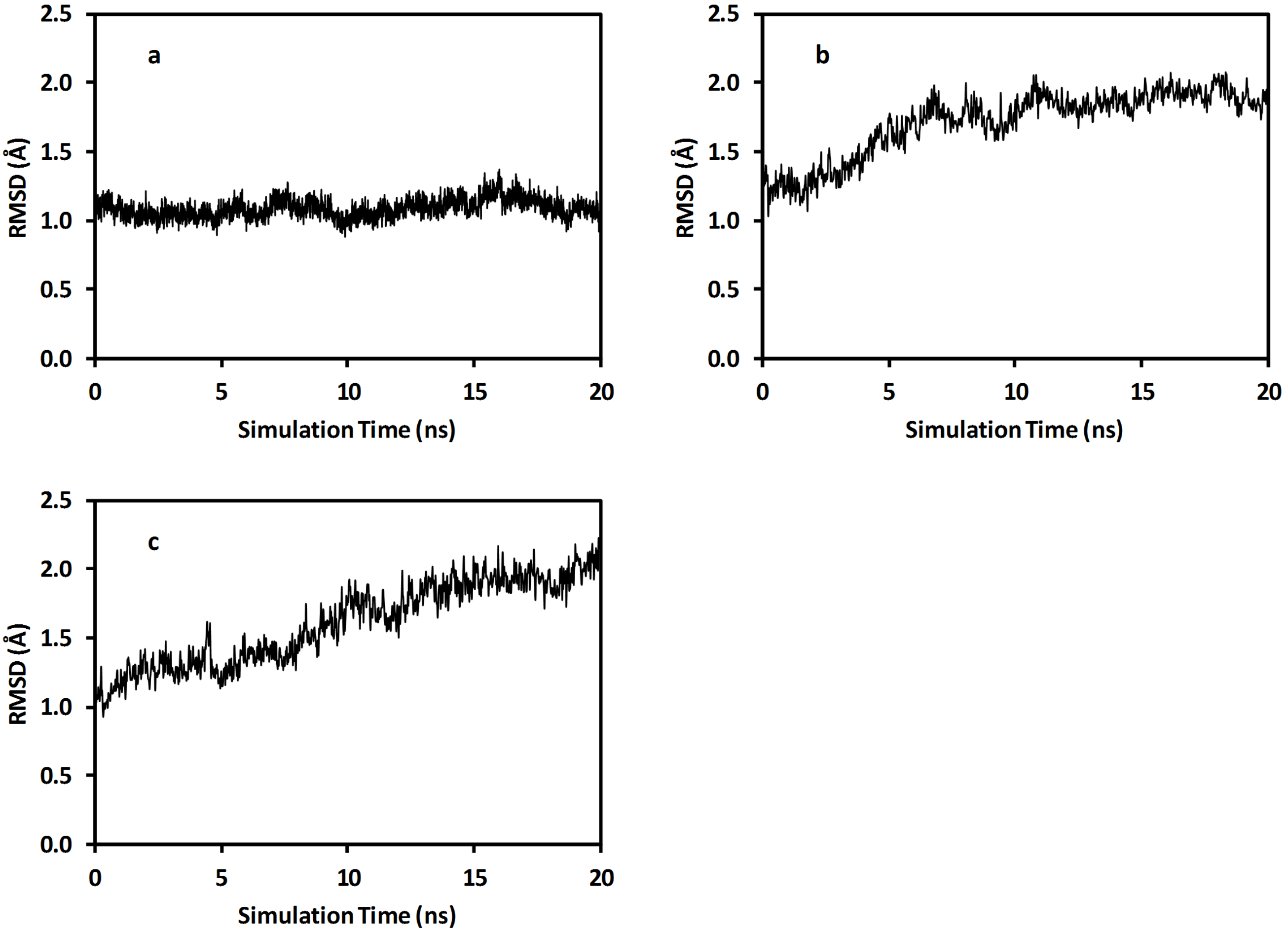

Figure S3. Backbone RMSD time series for 3ns NVT simulations in 0.3M NaCl background extended to 20 ns. (**a**) 300 K, without glycosylation. (**b**) 400 K, with glycosylation. (**c**) 400 K, without glycosylation. The curves include RMSD for the initial 3 ns simulations.

Time Series for SASA, RMSD, R_{gyr}, and Backbone HB for 3 ns NVT Simulations

NAG_0P0M_300K

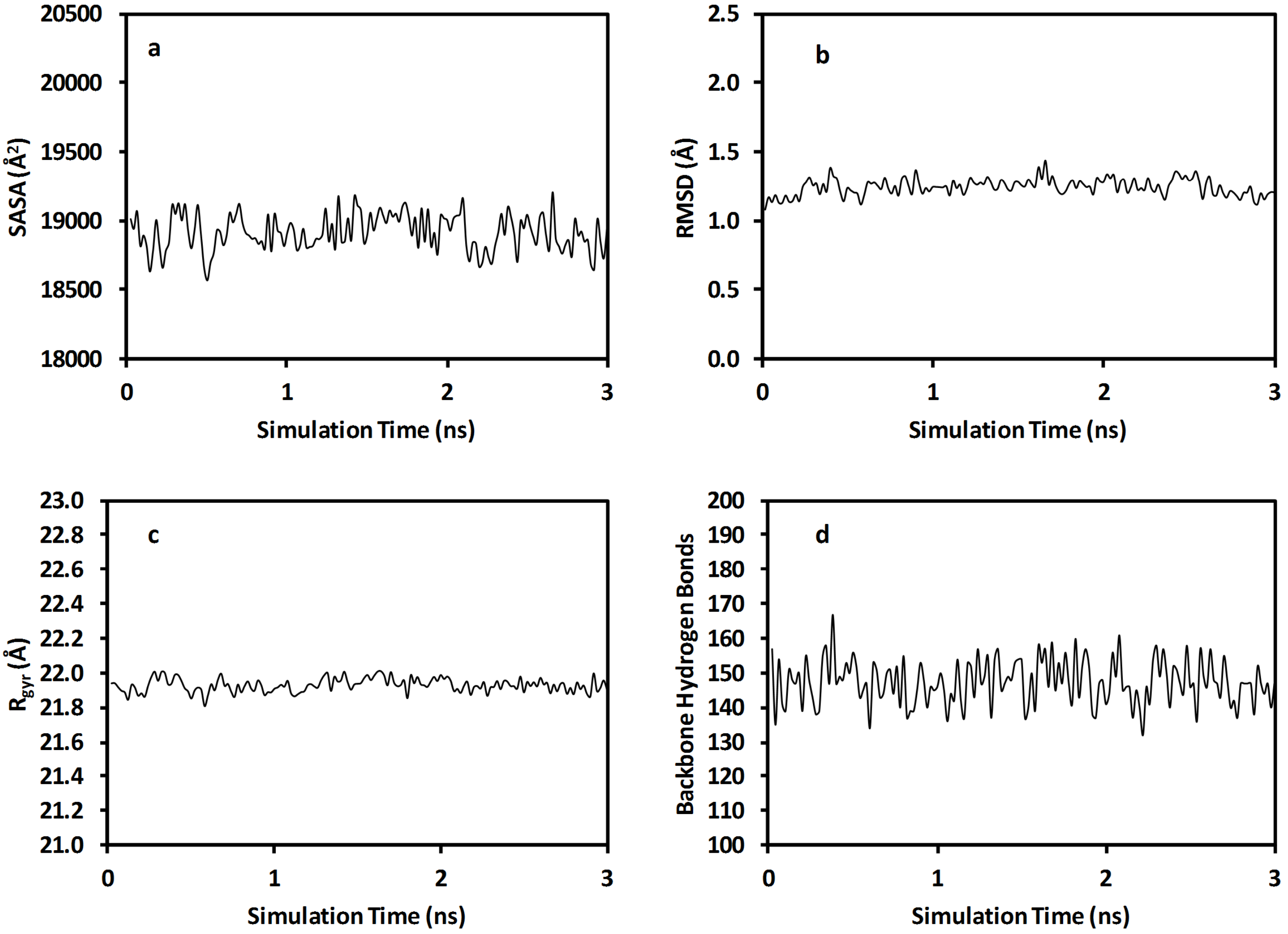

Figure S4. Time series of studied properties from 3 ns NVT simulations. a) SASA, b) Backbone RMSD, c) Radius of Gyration, d) Backbone hydrogen bonds.

NAG_0P0M_350K

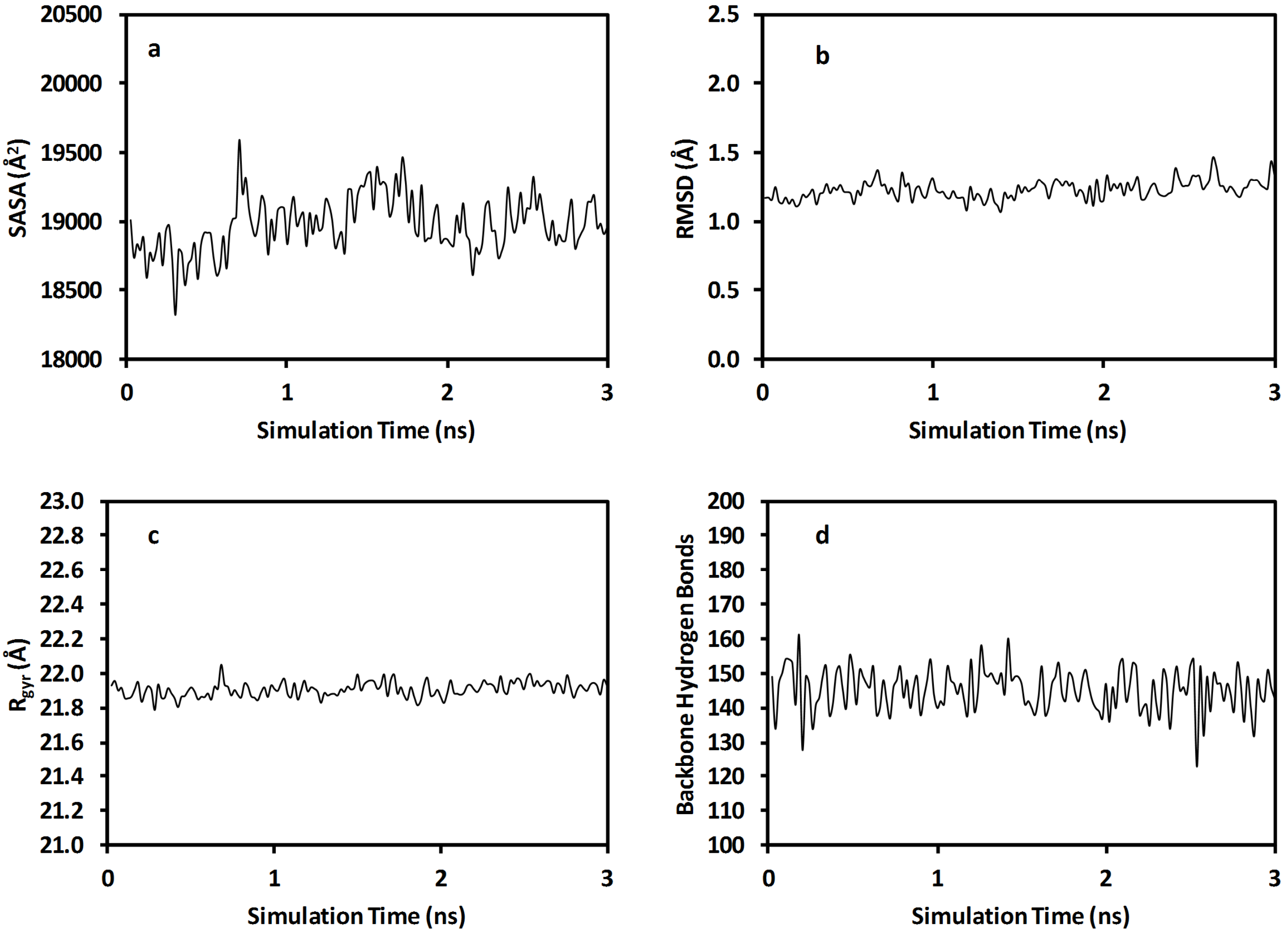

Figure S5. Time series of studied properties from 3 ns NVT simulations. a) SASA, b) Backbone RMSD, c) Radius of Gyration, d) Backbone hydrogen bonds.

NAG_0P0M_400K

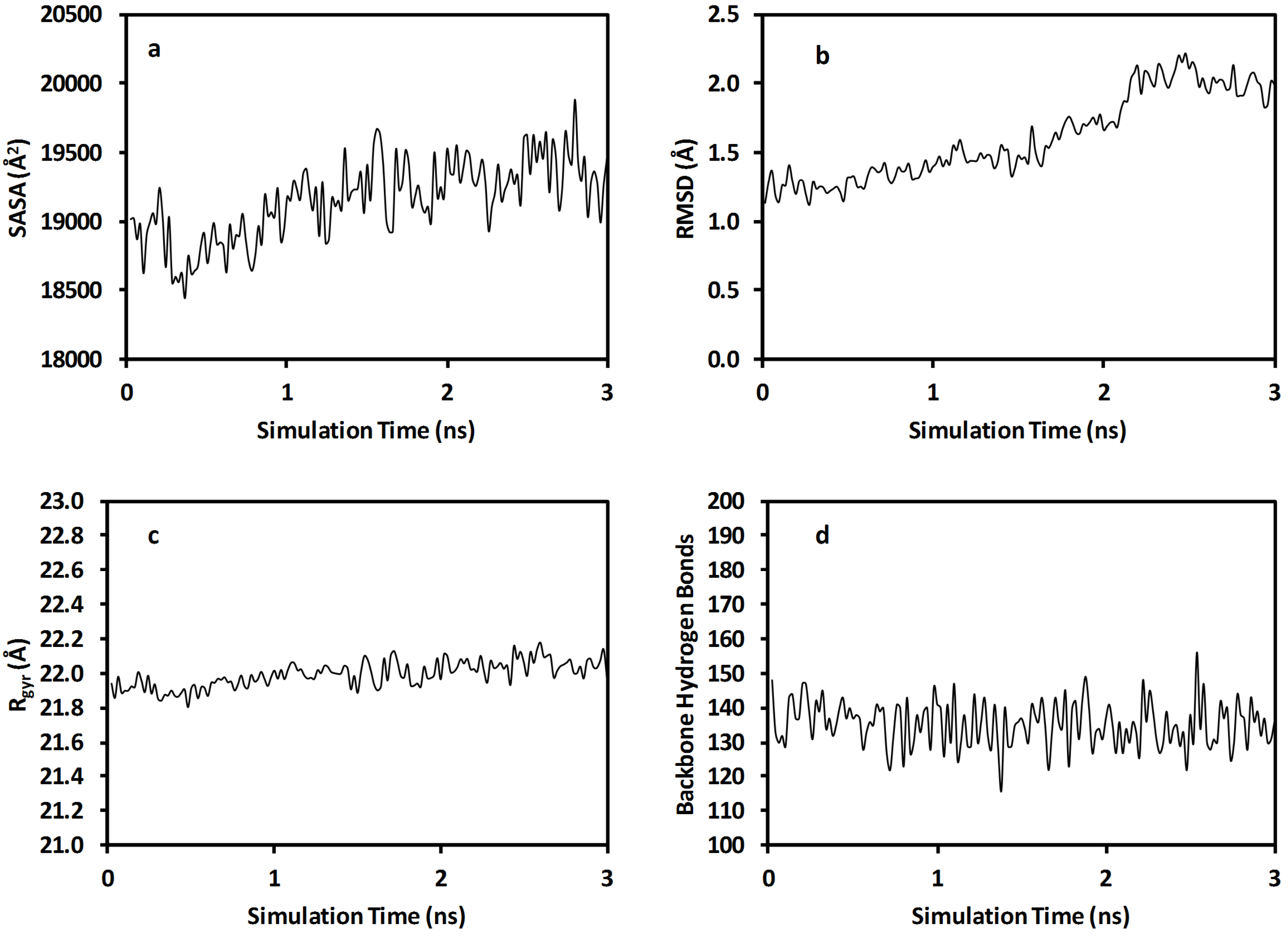

Figure S6. Time series of studied properties from 3 ns NVT simulations. a) SASA, b) Backbone RMSD, c) Radius of Gyration, d) Backbone hydrogen bonds.

noNAG_0P0M_300K

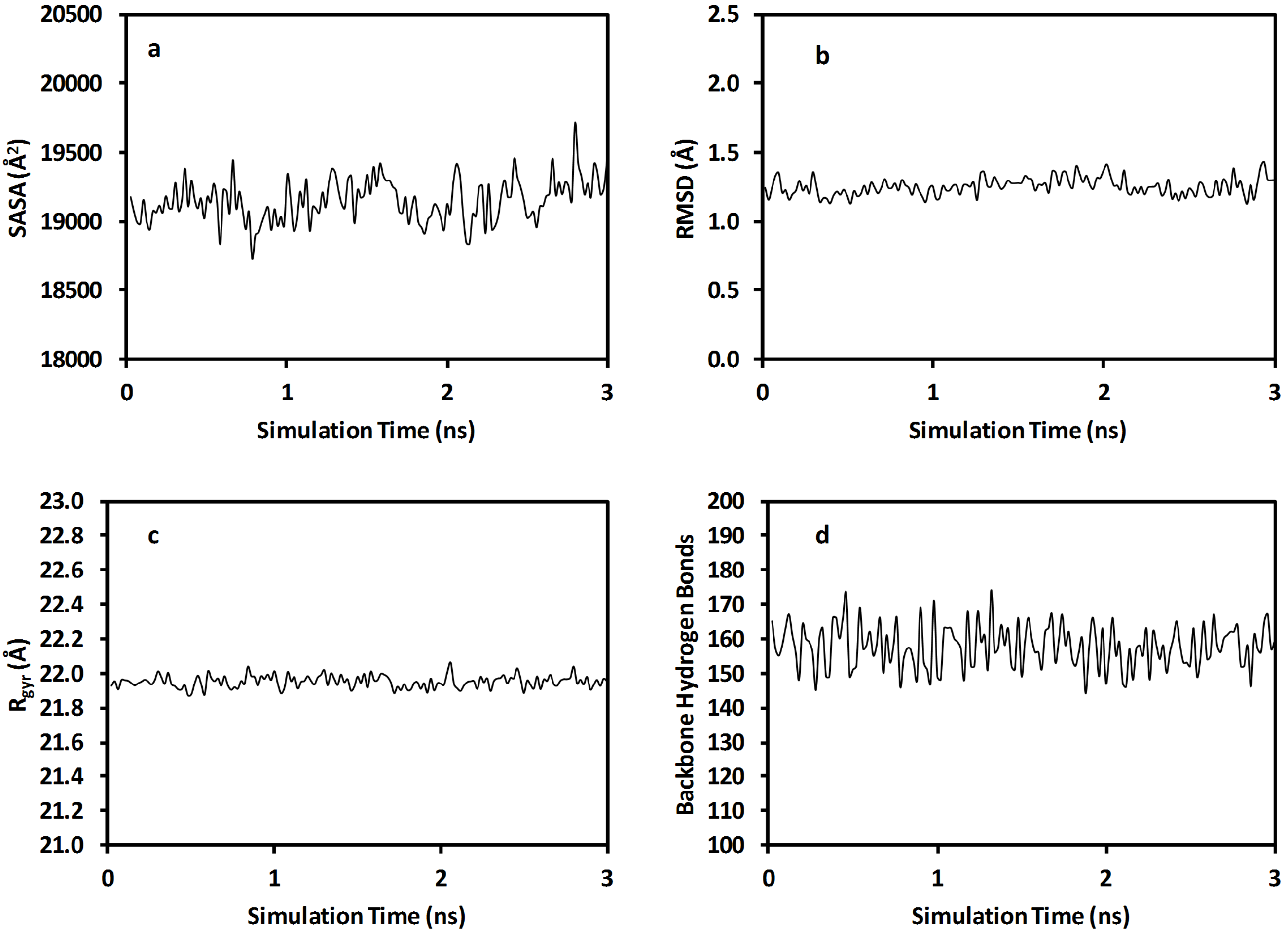

Figure S7. Time series of studied properties from 3 ns NVT simulations. a) SASA, b) Backbone RMSD, c) Radius of Gyration, d) Backbone hydrogen bonds.

noNAG_0P0M_350K

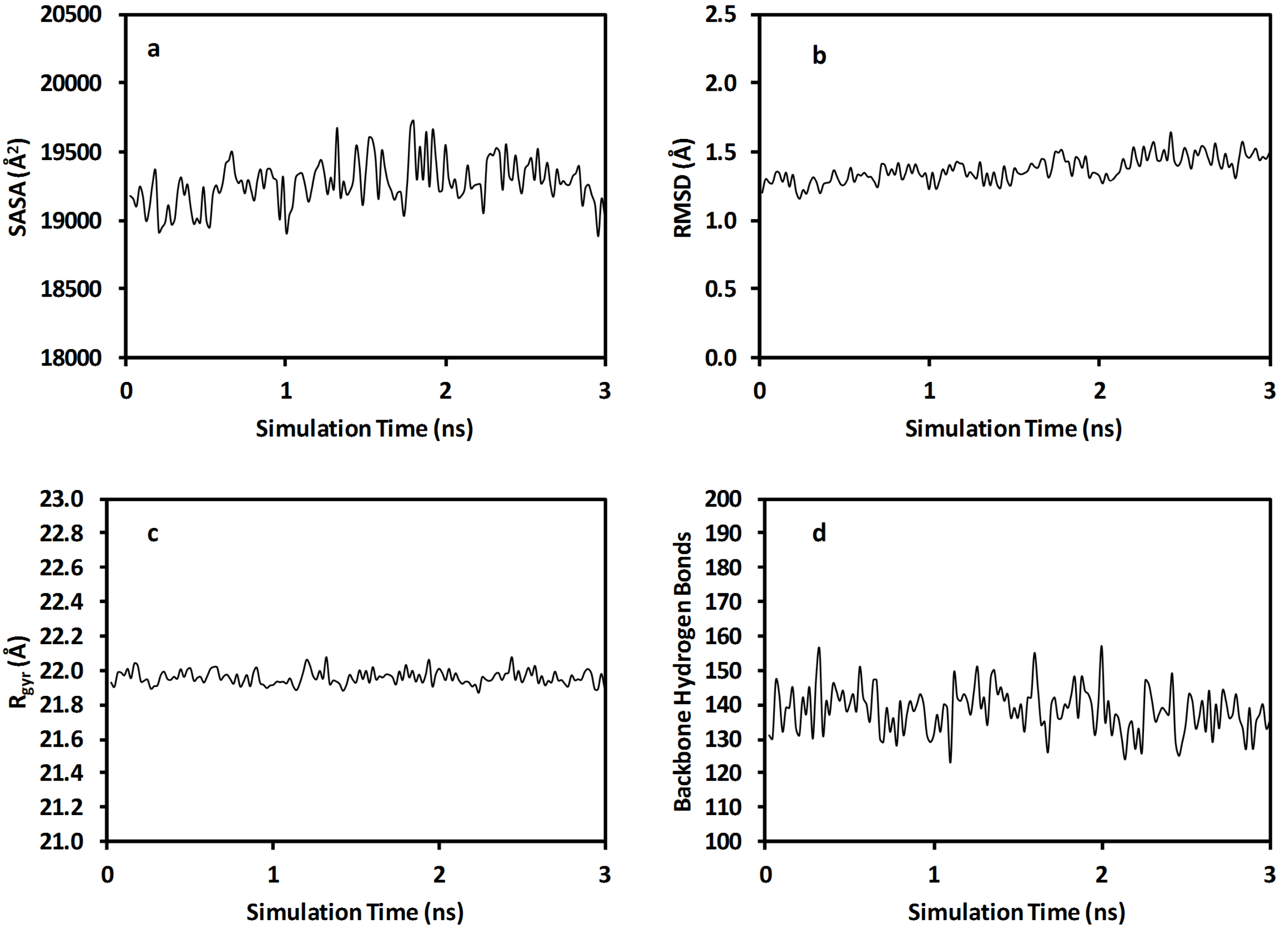

Figure S8 . Time series of studied properties from 3 ns NVT simulations. a) SASA, b) Backbone RMSD, c) Radius of Gyration, d) Backbone hydrogen bonds.

noNAG_0P0M_400K

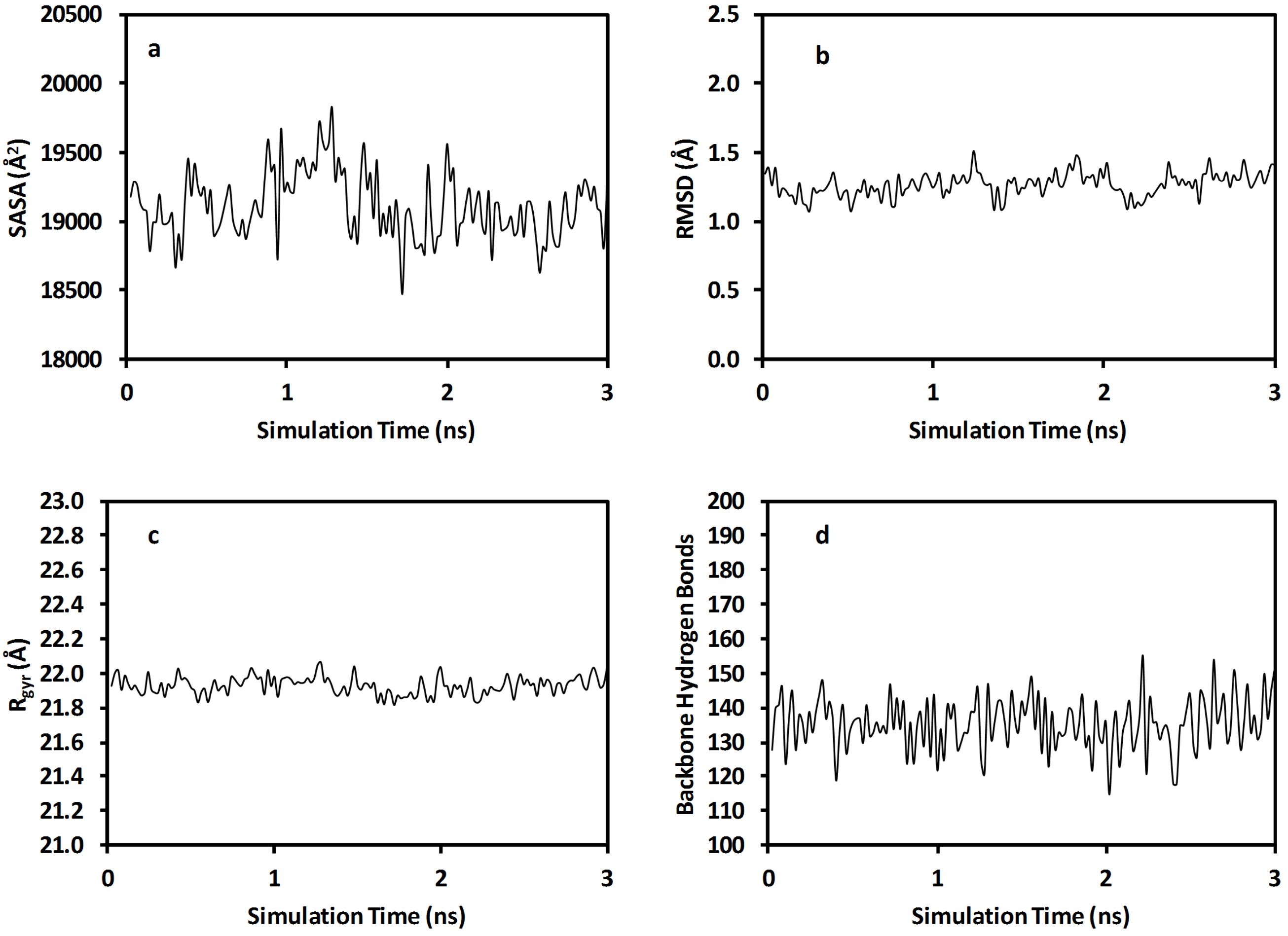

Figure S9. Time series of studied properties from 3 ns NVT simulations. a) SASA, b) Backbone RMSD, c) Radius of Gyration, d) Backbone hydrogen bonds.

NAG_0P3M_NACL_300K

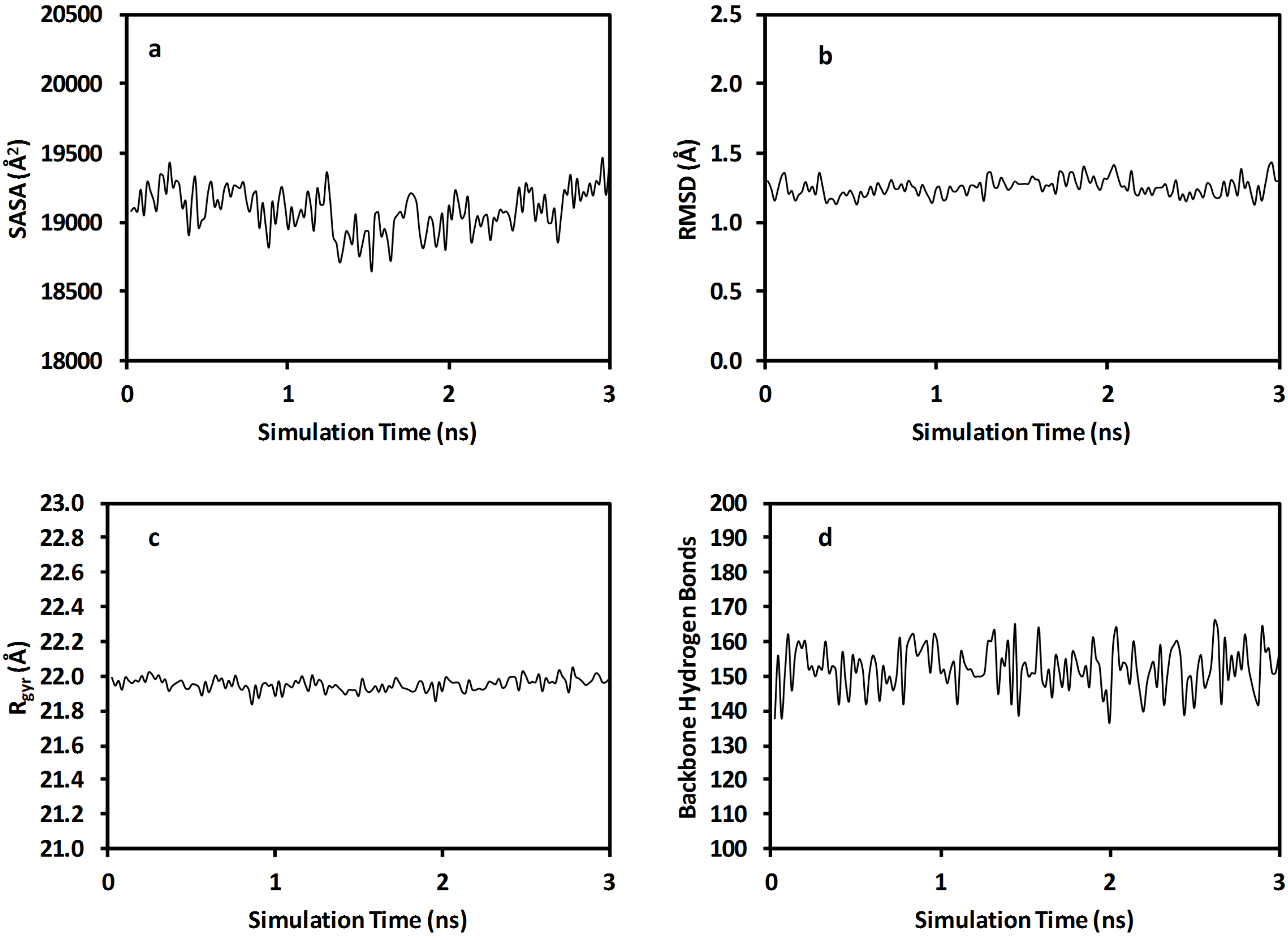

Figure S10. Time series of studied properties from 3 ns NVT simulations. a) SASA, b) Backbone RMSD, c) Radius of Gyration, d) Backbone hydrogen bonds.

NAG_0P3M_NACL_350K

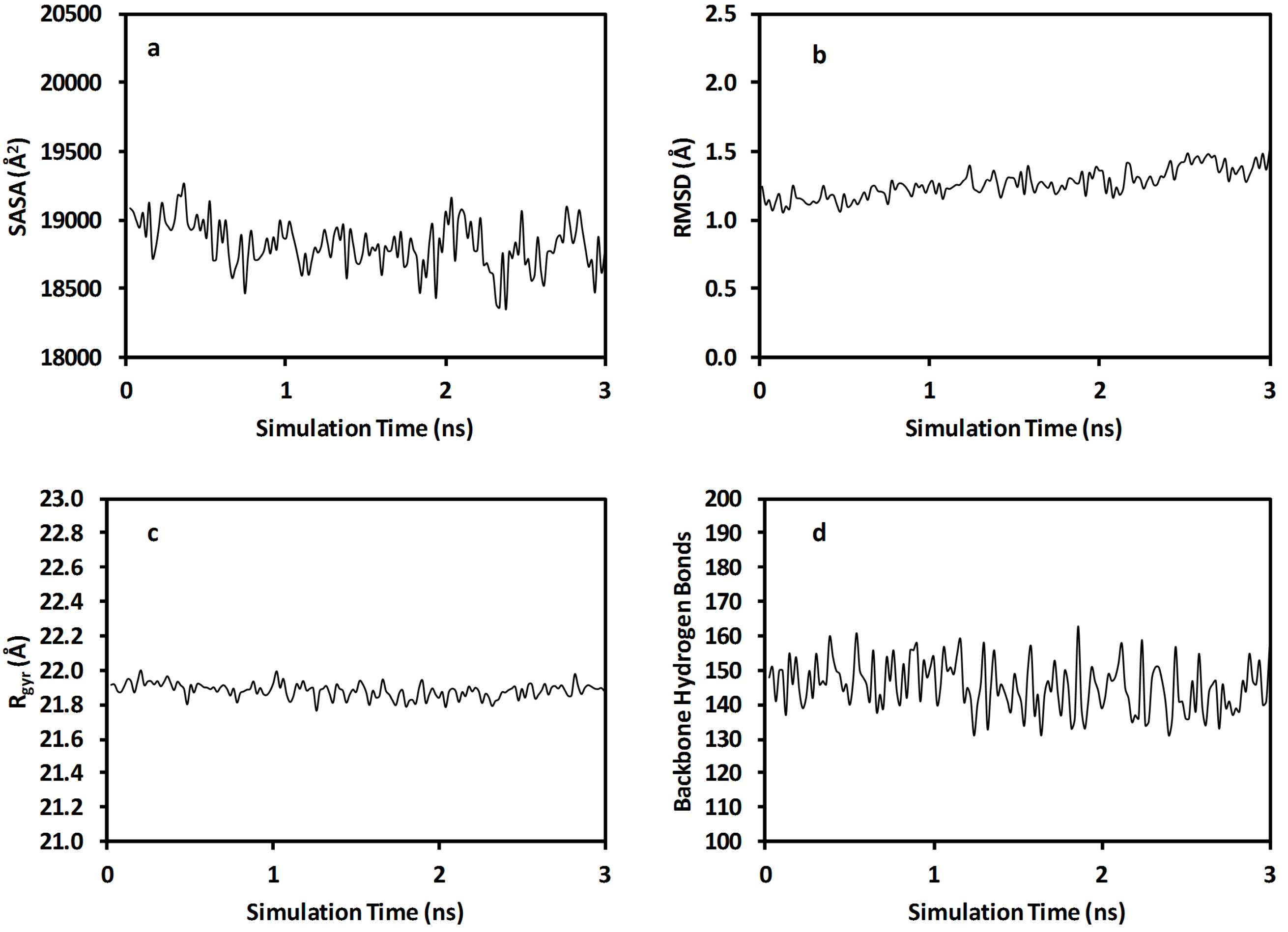

Figure S11. Time series of studied properties from 3 ns NVT simulations. a) SASA, b) Backbone RMSD, c) Radius of Gyration, d) Backbone hydrogen bonds.

NAG_0P3M_NACL_400K

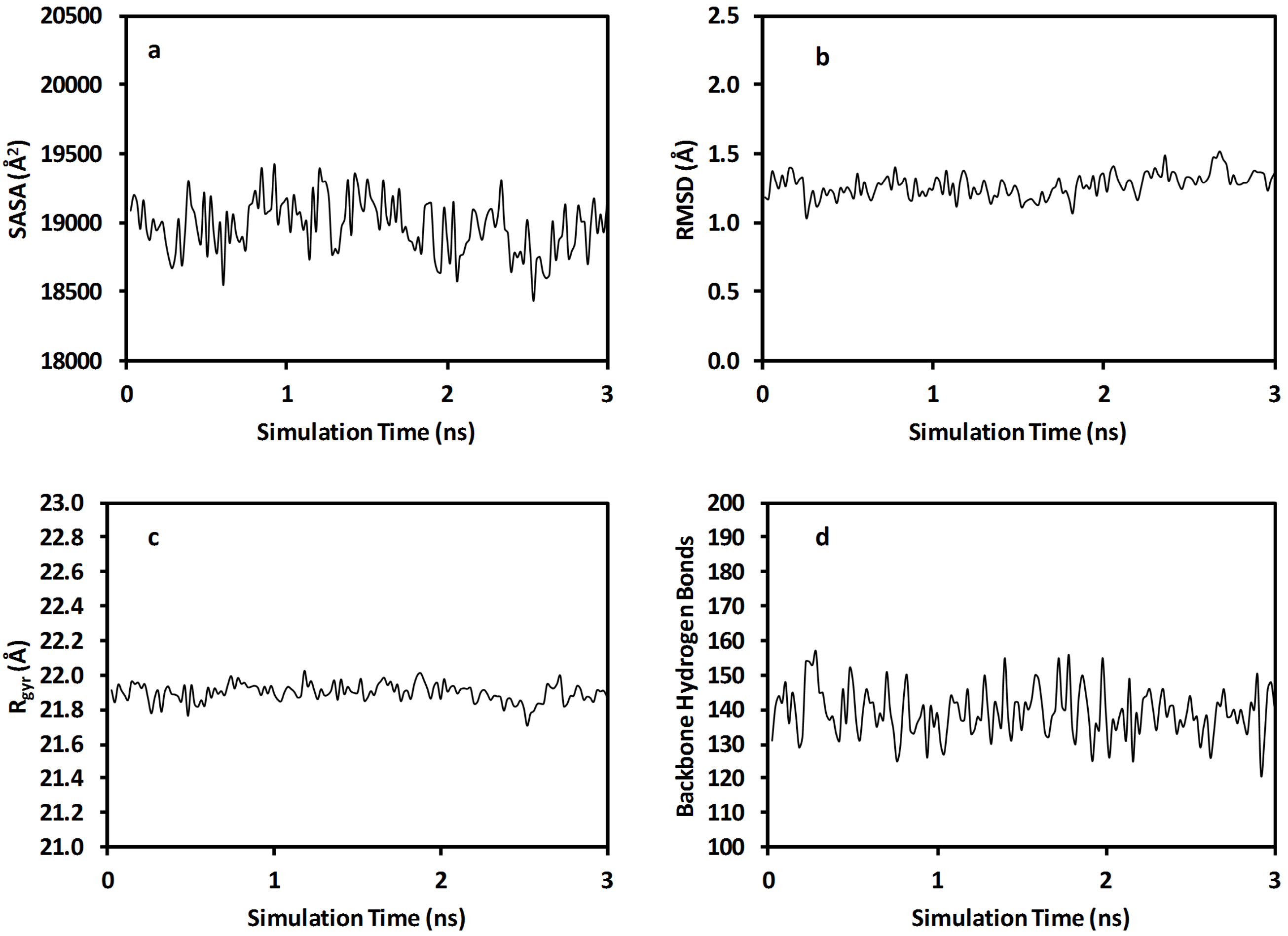

Figure S12. Time series of studied properties from 3 ns NVT simulations. a) SASA, b) Backbone RMSD, c) Radius of Gyration, d) Backbone hydrogen bonds.

noNAG_0P3M_NACL_300K

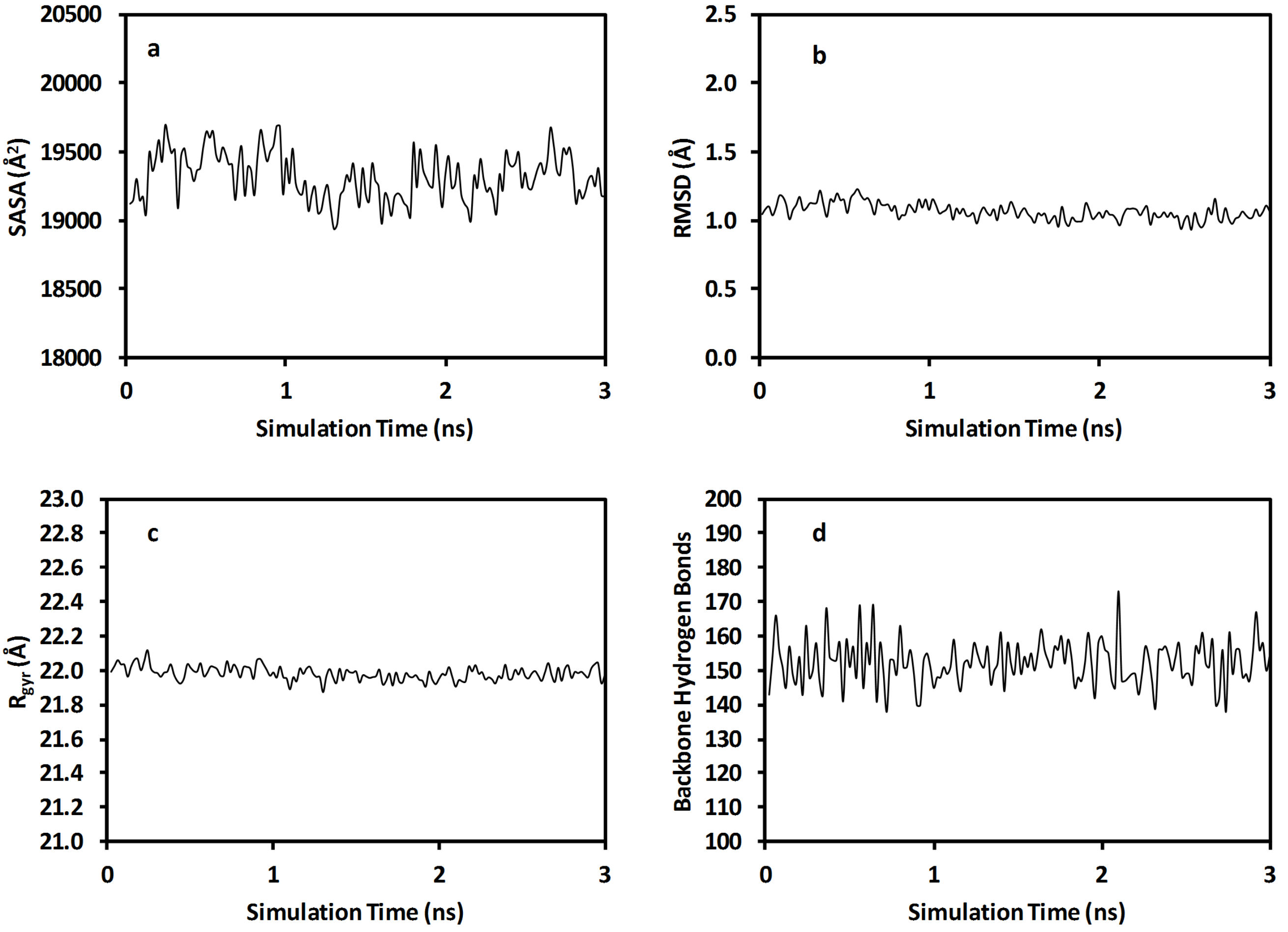

Figure S13. Time series of studied properties from 3 ns NVT simulations. a) SASA, b) Backbone RMSD, c) Radius of Gyration, d) Backbone hydrogen bonds.

noNAG_0P3M_NACL_350K

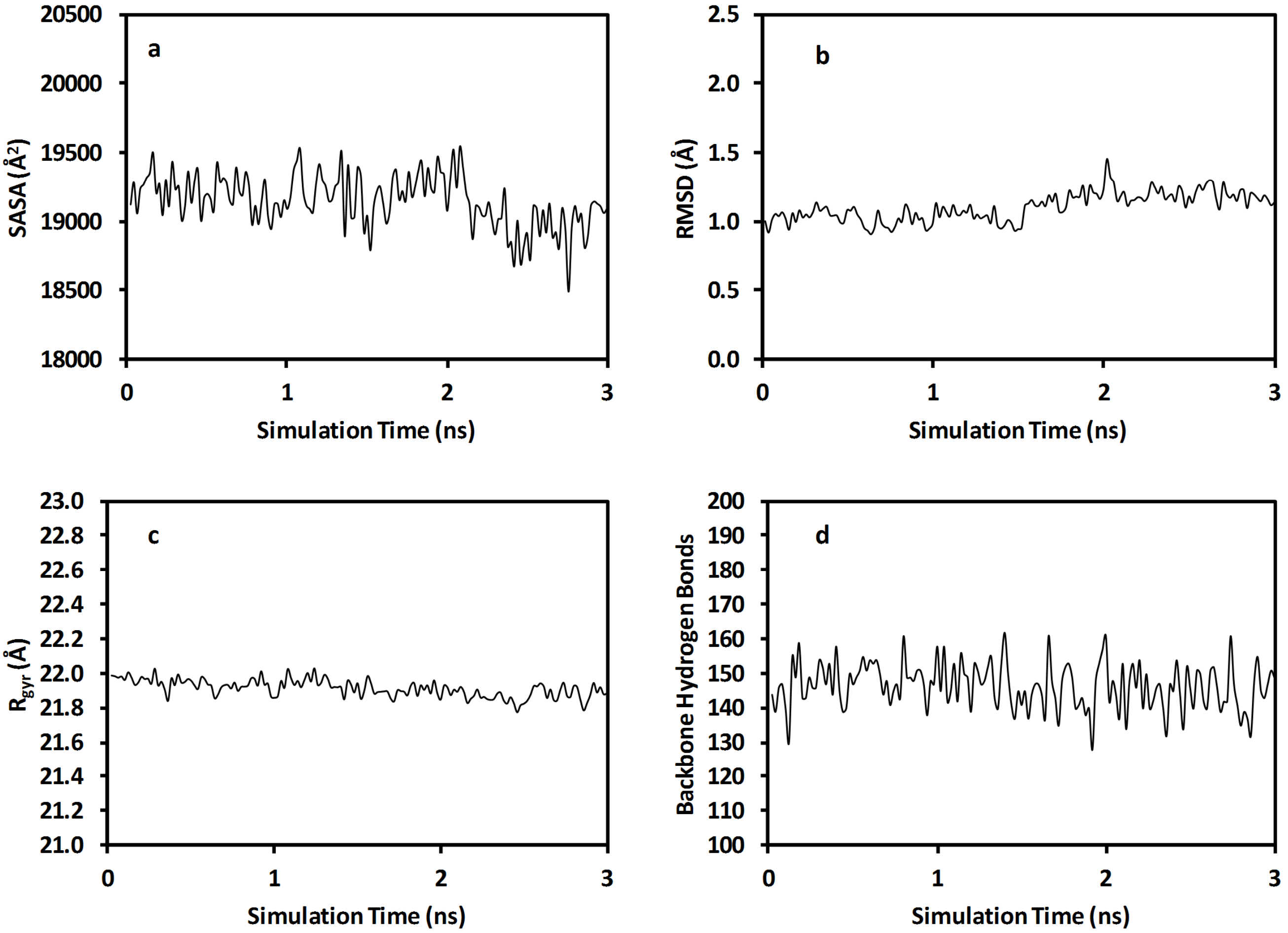

Figure S14. Time series of studied properties from 3 ns NVT simulations. a) SASA, b) Backbone RMSD, c) Radius of Gyration, d) Backbone hydrogen bonds.

noNAG_0P3M_NACL_400K

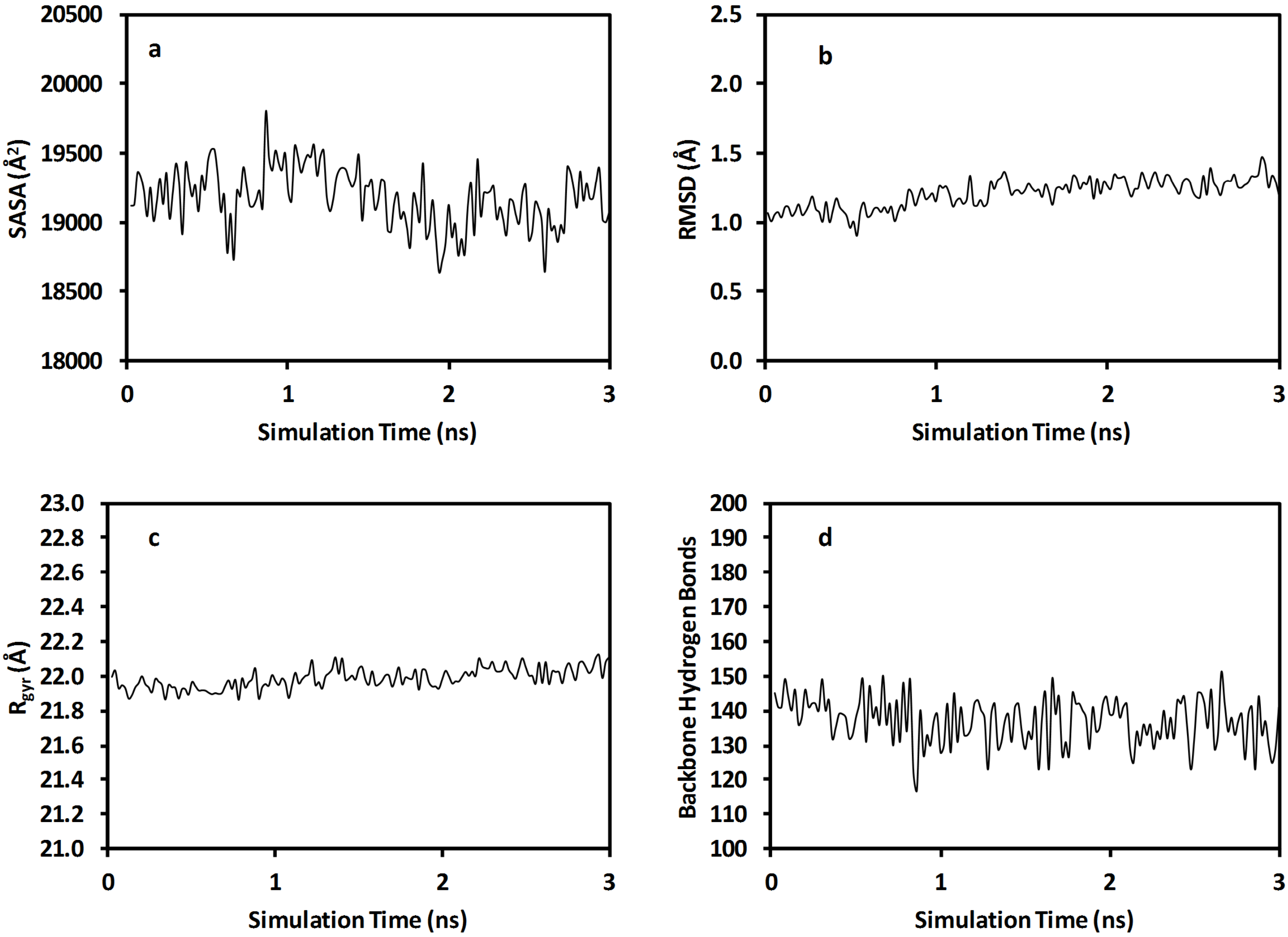

Figure S15. Time series of studied properties from 3 ns NVT simulations. a) SASA, b) Backbone RMSD, c) Radius of Gyration, d) Backbone hydrogen bonds.

NAG_0P3M_KF_300K

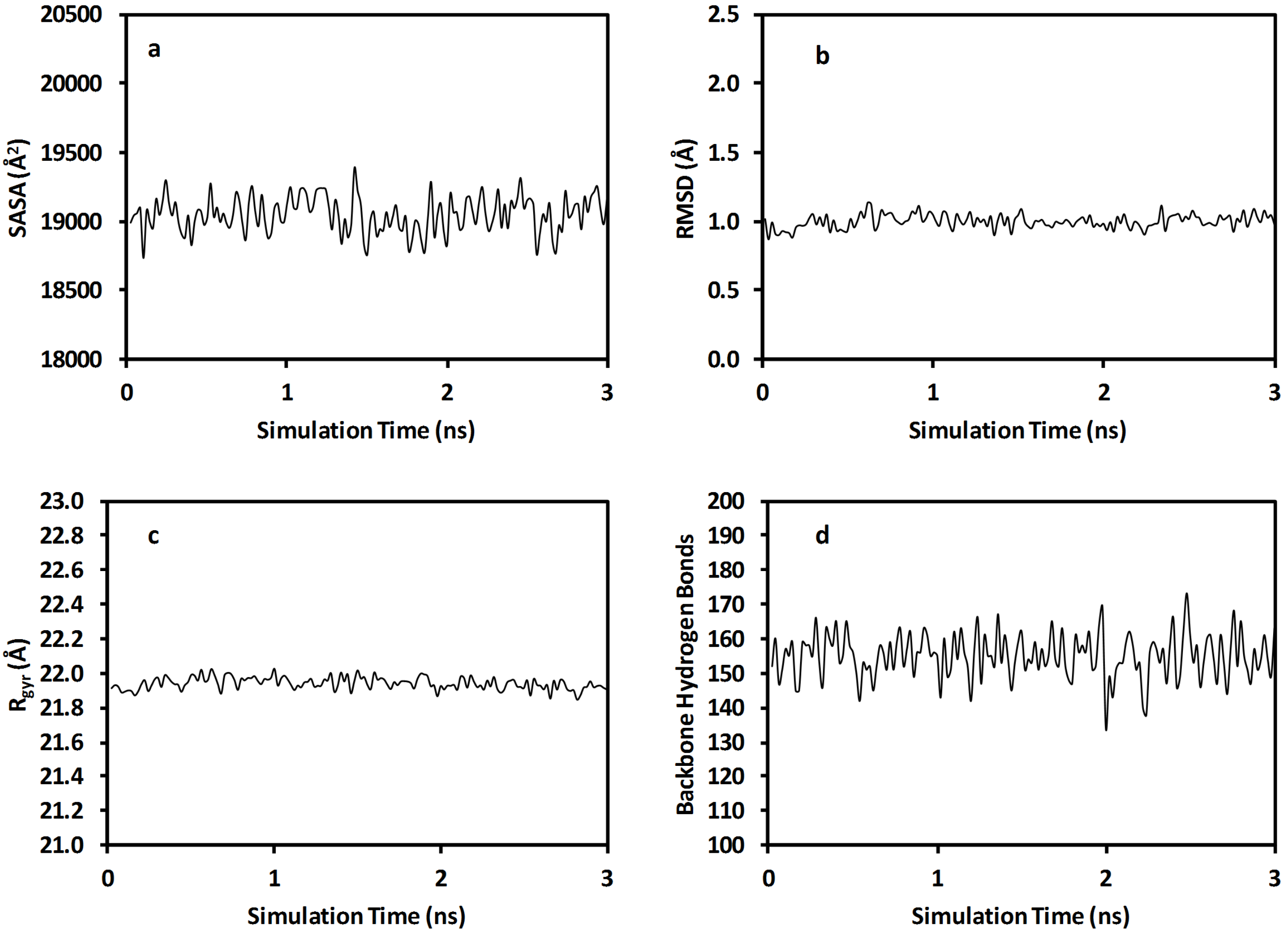

Figure S16. Time series of studied properties from 3 ns NVT simulations. a) SASA, b) Backbone RMSD, c) Radius of Gyration, d) Backbone hydrogen bonds.

NAG_0P3M_KF_350K

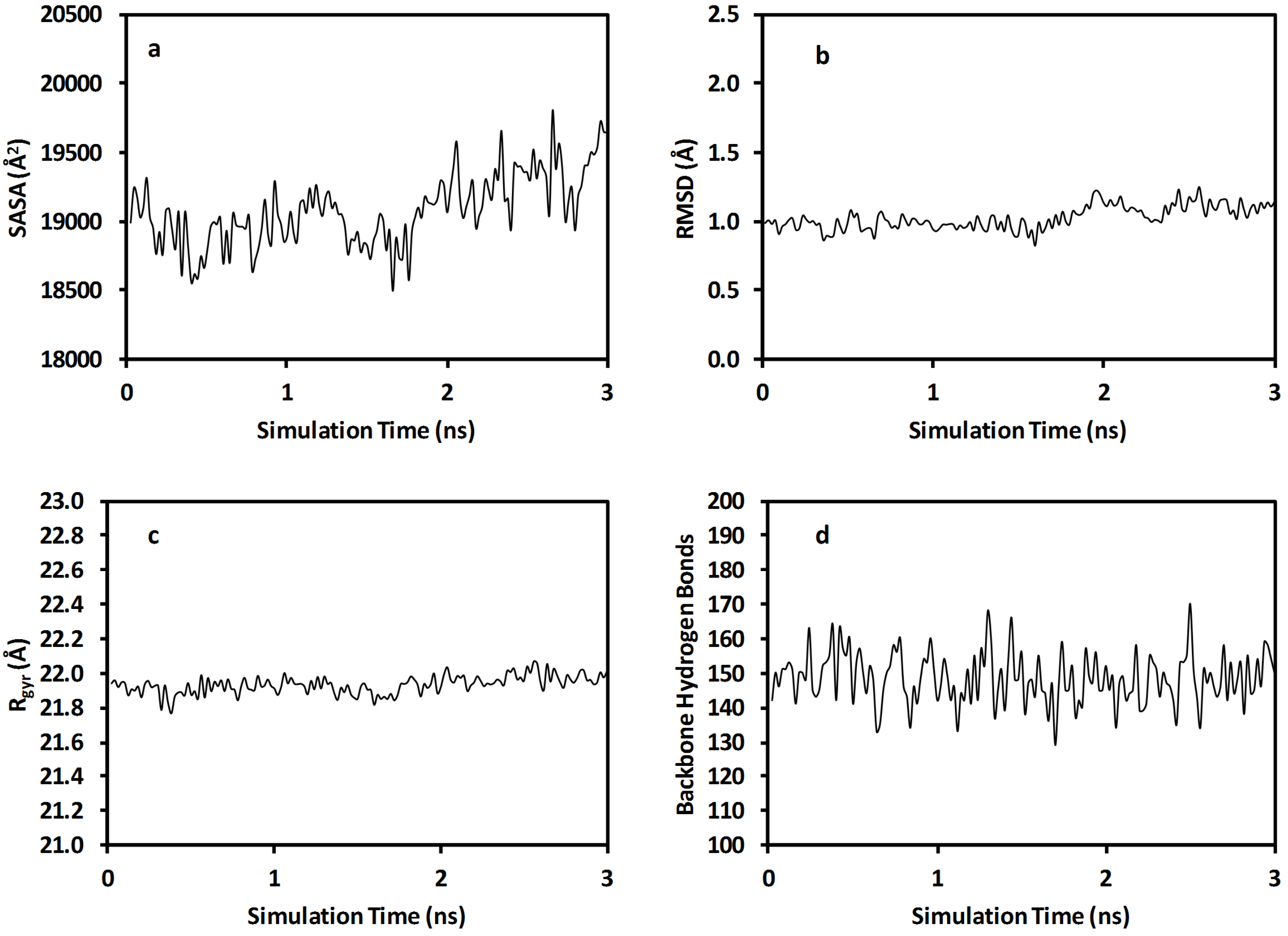

Figure S17. Time series of studied properties from 3 ns NVT simulations. a) SASA, b) Backbone RMSD, c) Radius of Gyration, d) Backbone hydrogen bonds.

NAG_0P3M_KF_400K

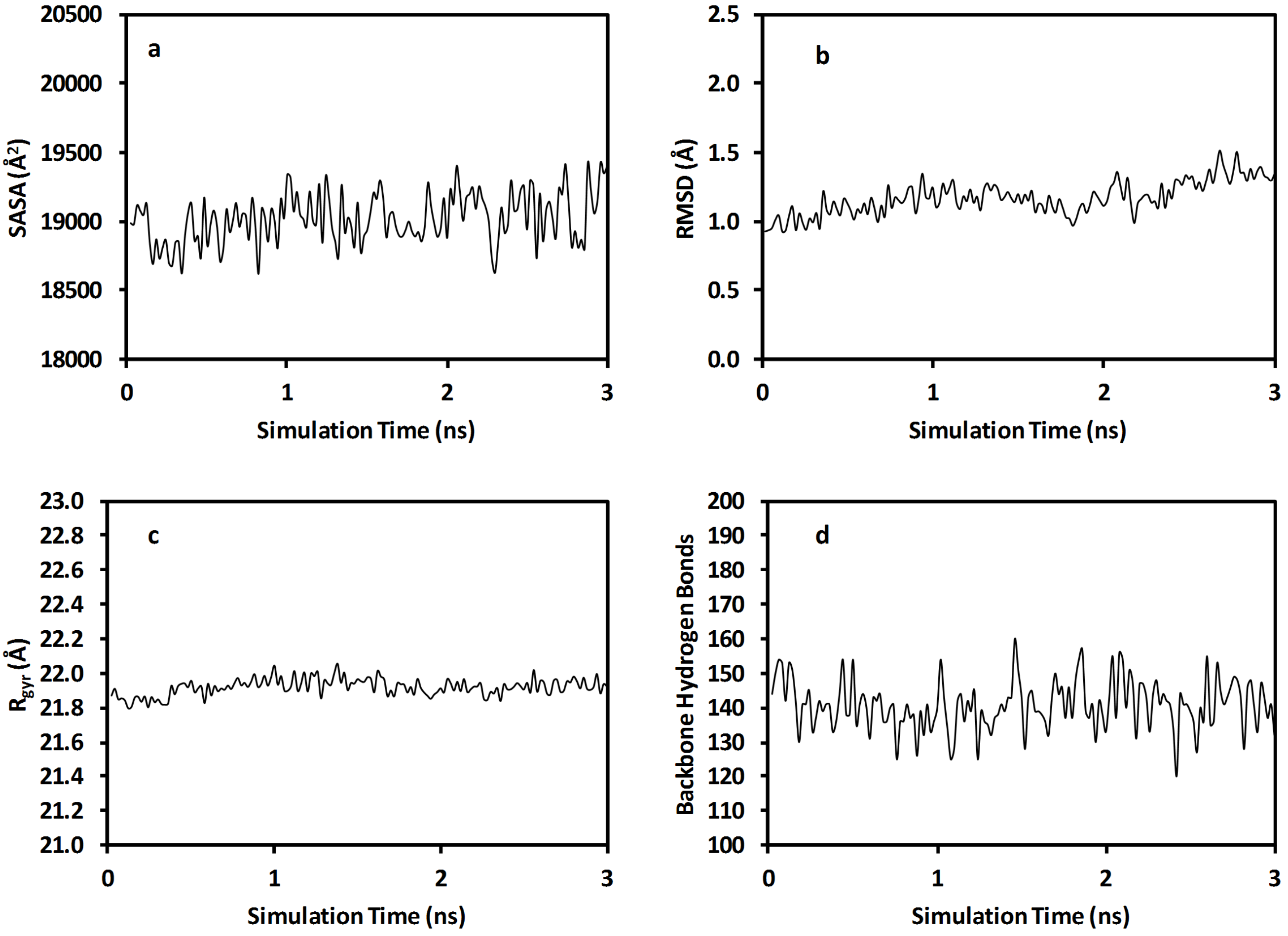

Figure S18. Time series of studied properties from 3 ns NVT simulations. a) SASA, b) Backbone RMSD, c) Radius of Gyration, d) Backbone hydrogen bonds.

noNAG_0P3M_KF_300K

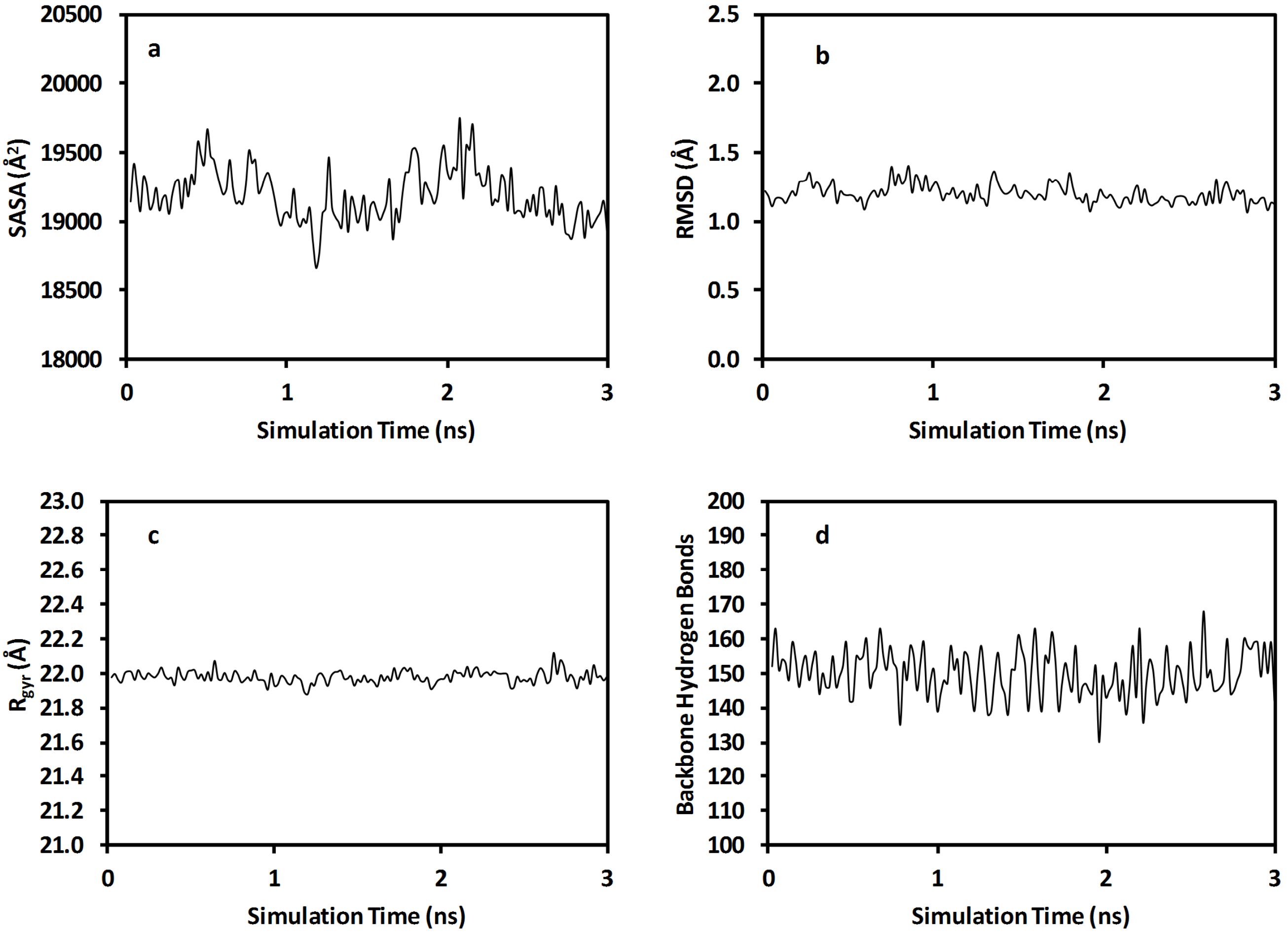

Figure S19. Time series of studied properties from 3 ns NVT simulations. a) SASA, b) Backbone RMSD, c) Radius of Gyration, d) Backbone hydrogen bonds.

noNAG_0P3M_KF_350K

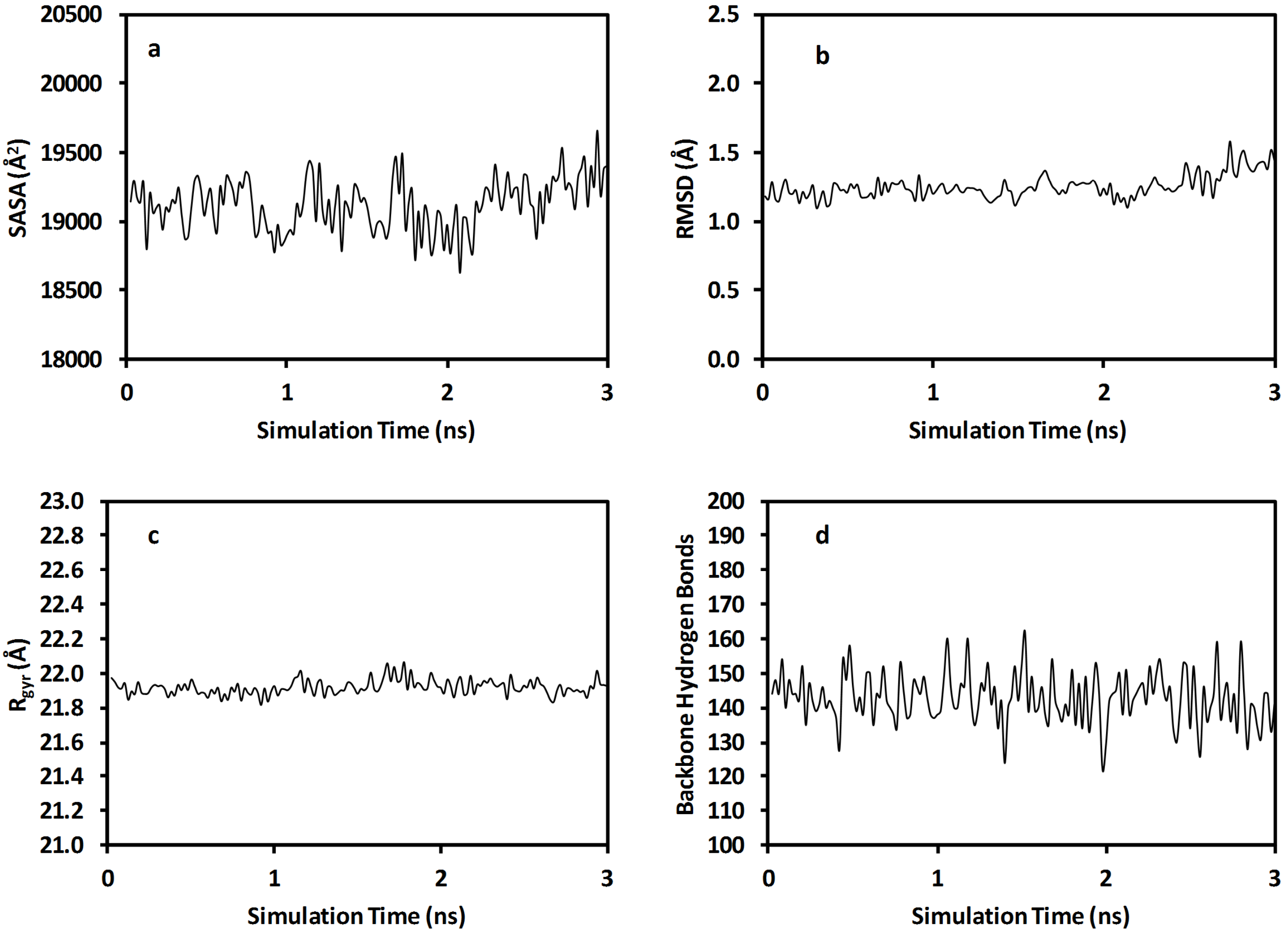

Figure S20. Time series of studied properties from 3 ns NVT simulations. a) SASA, b) Backbone RMSD, c) Radius of Gyration, d) Backbone hydrogen bonds.

noNAG_0P3M_KF_400K

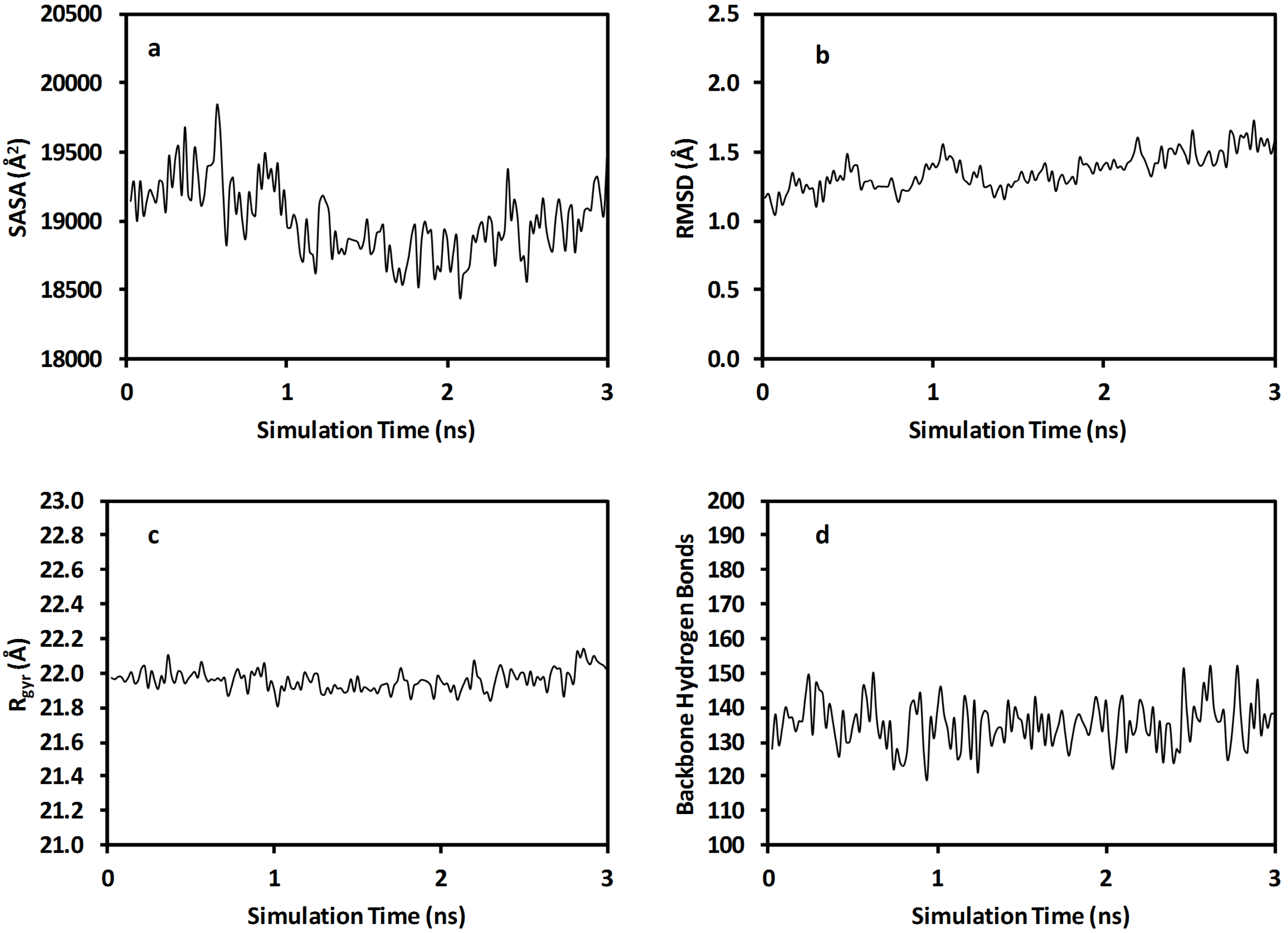

Figure S21. Time series of studied properties from 3 ns NVT simulations. a) SASA, b) Backbone RMSD, c) Radius of Gyration, d) Backbone hydrogen bonds.

NAG_1P2M_NACL_300K

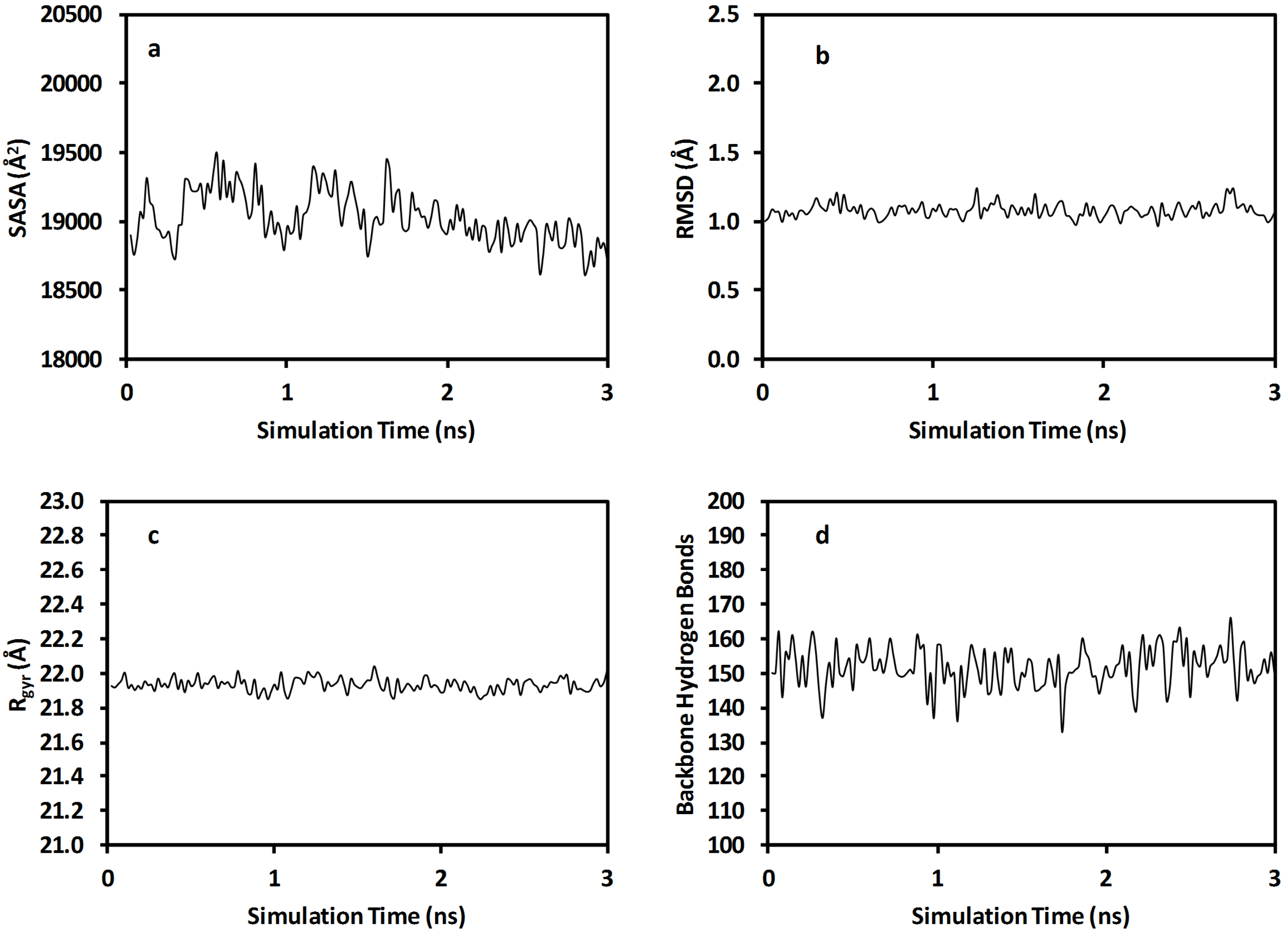

Figure S22. Time series of studied properties from 3 ns NVT simulations. a) SASA, b) Backbone RMSD, c) Radius of Gyration, d) Backbone hydrogen bonds.

NAG_1P2M_NACL_350K

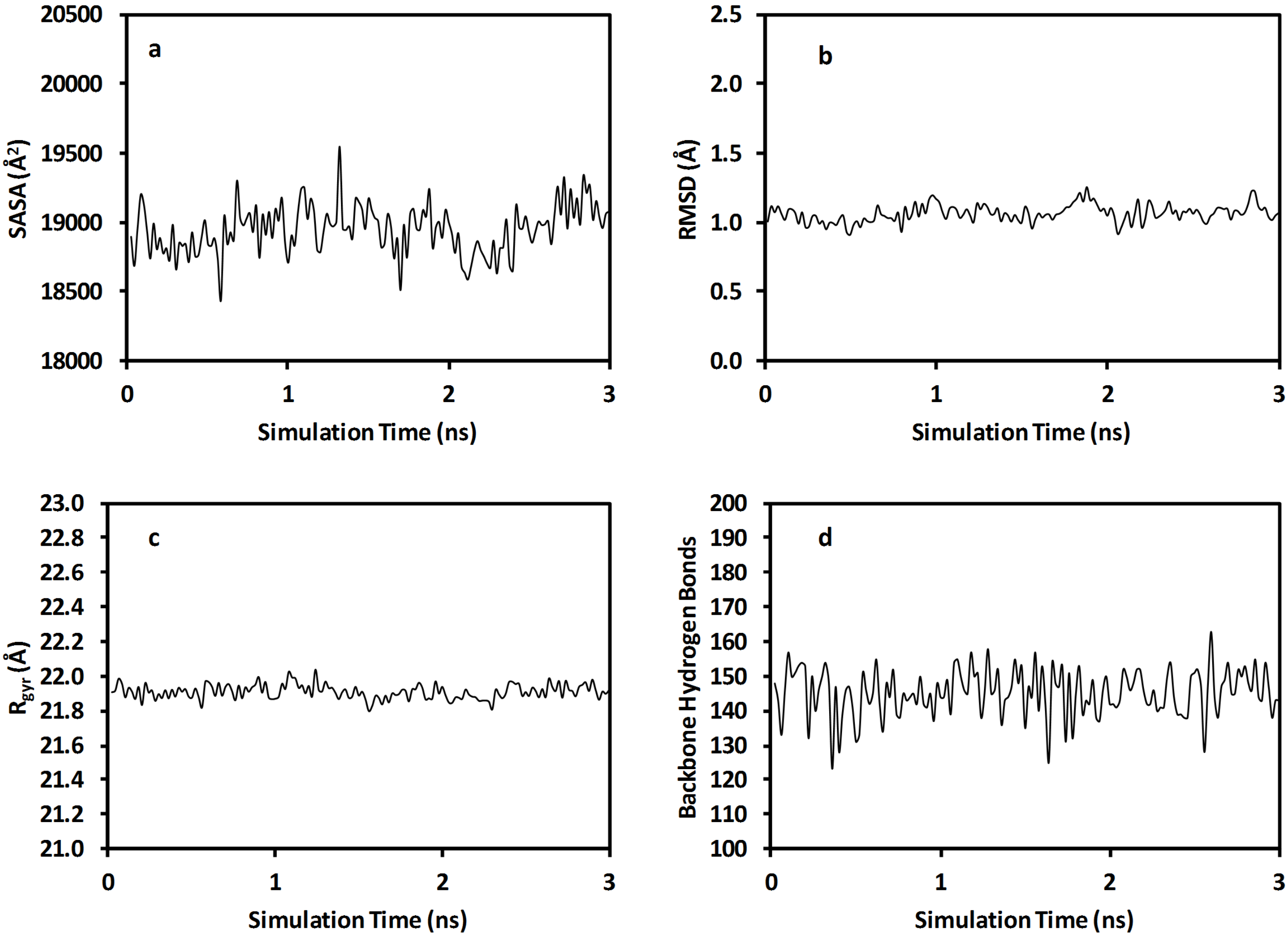

Figure S23. Time series of studied properties from 3 ns NVT simulations. a) SASA, b) Backbone RMSD, c) Radius of Gyration, d) Backbone hydrogen bonds.

NAG_1P2M_NACL_400K

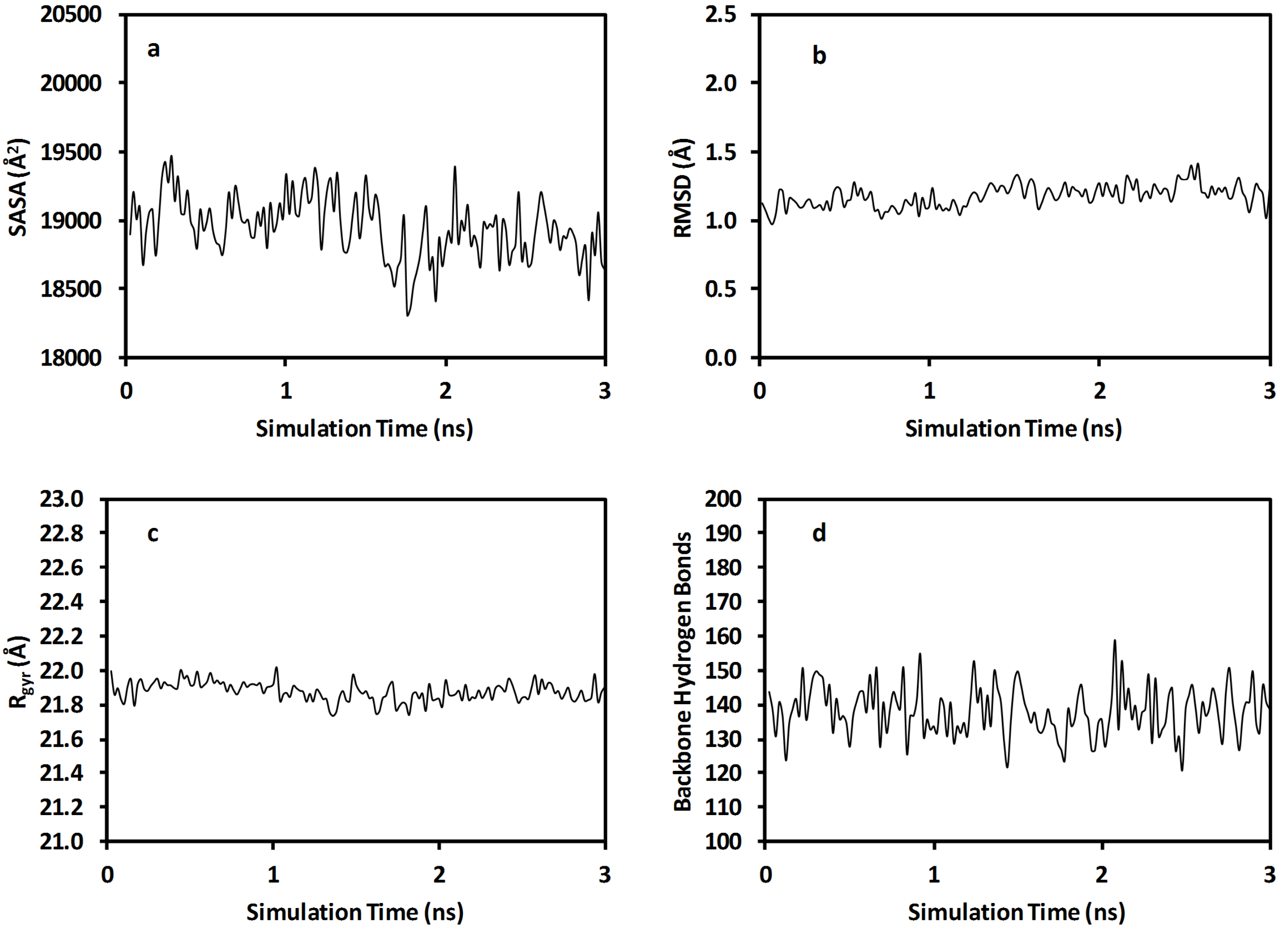

Figure S24. Time series of studied properties from 3 ns NVT simulations. a) SASA, b) Backbone RMSD, c) Radius of Gyration, d) Backbone hydrogen bonds.

noNAG_1P2M_NACL_300K

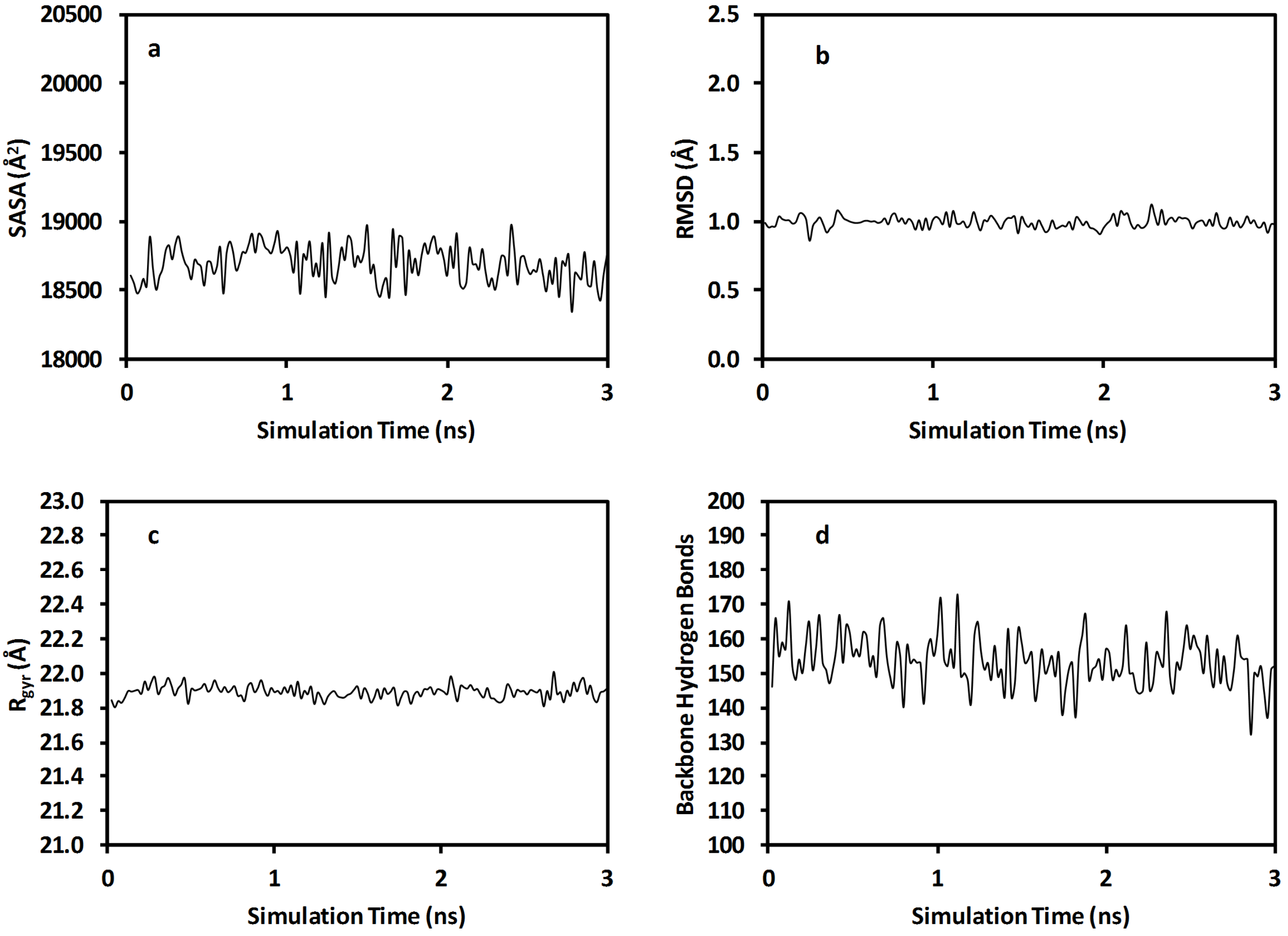

Figure S25. Time series of studied properties from 3 ns NVT simulations. a) SASA, b) Backbone RMSD, c) Radius of Gyration, d) Backbone hydrogen bonds.

noNAG_1P2M_NACL_350K

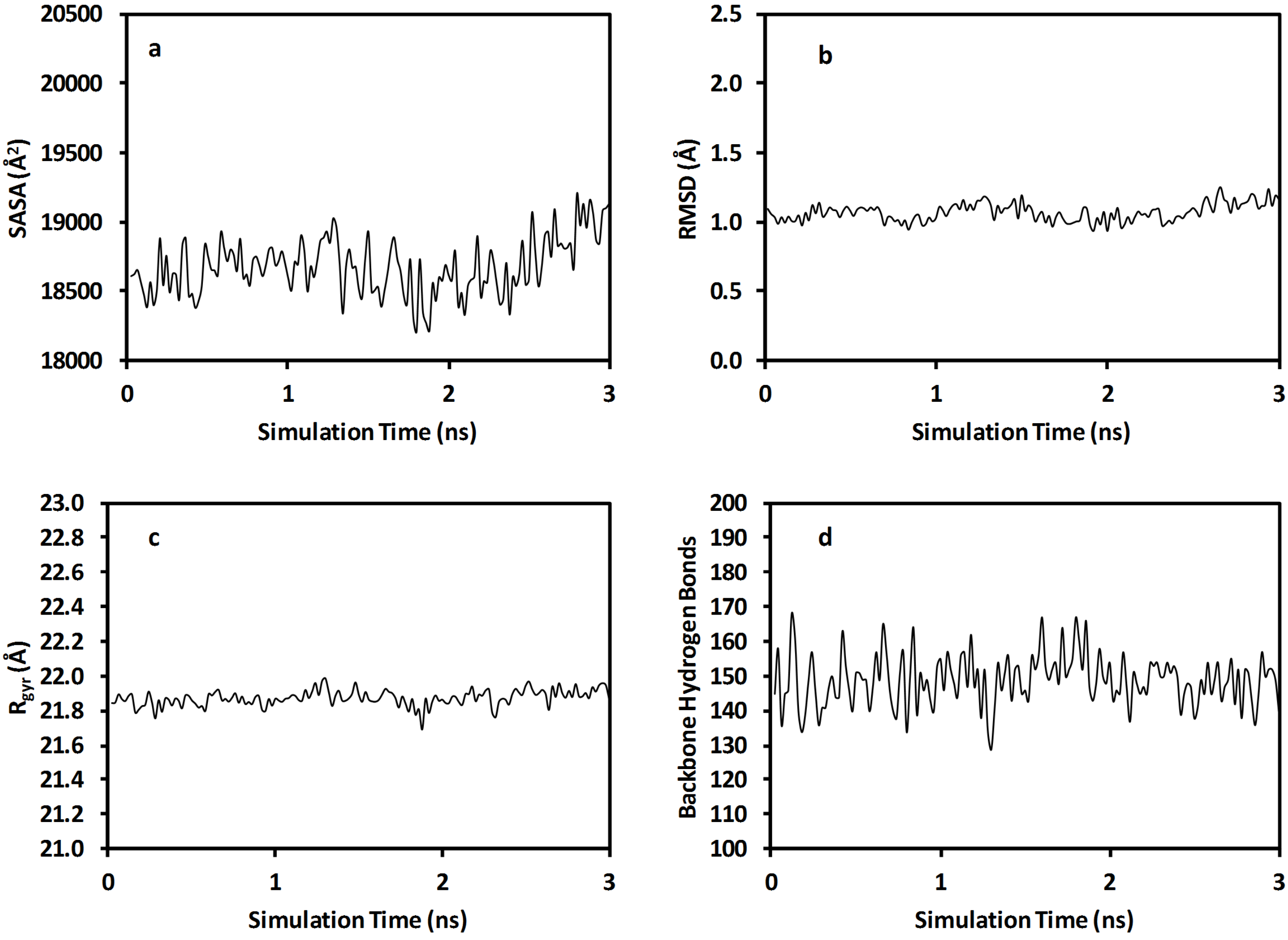

Figure S26. Time series of studied properties from 3 ns NVT simulations. a) SASA, b) Backbone RMSD, c) Radius of Gyration, d) Backbone hydrogen bonds.

noNAG_1P2M_NACL_400K

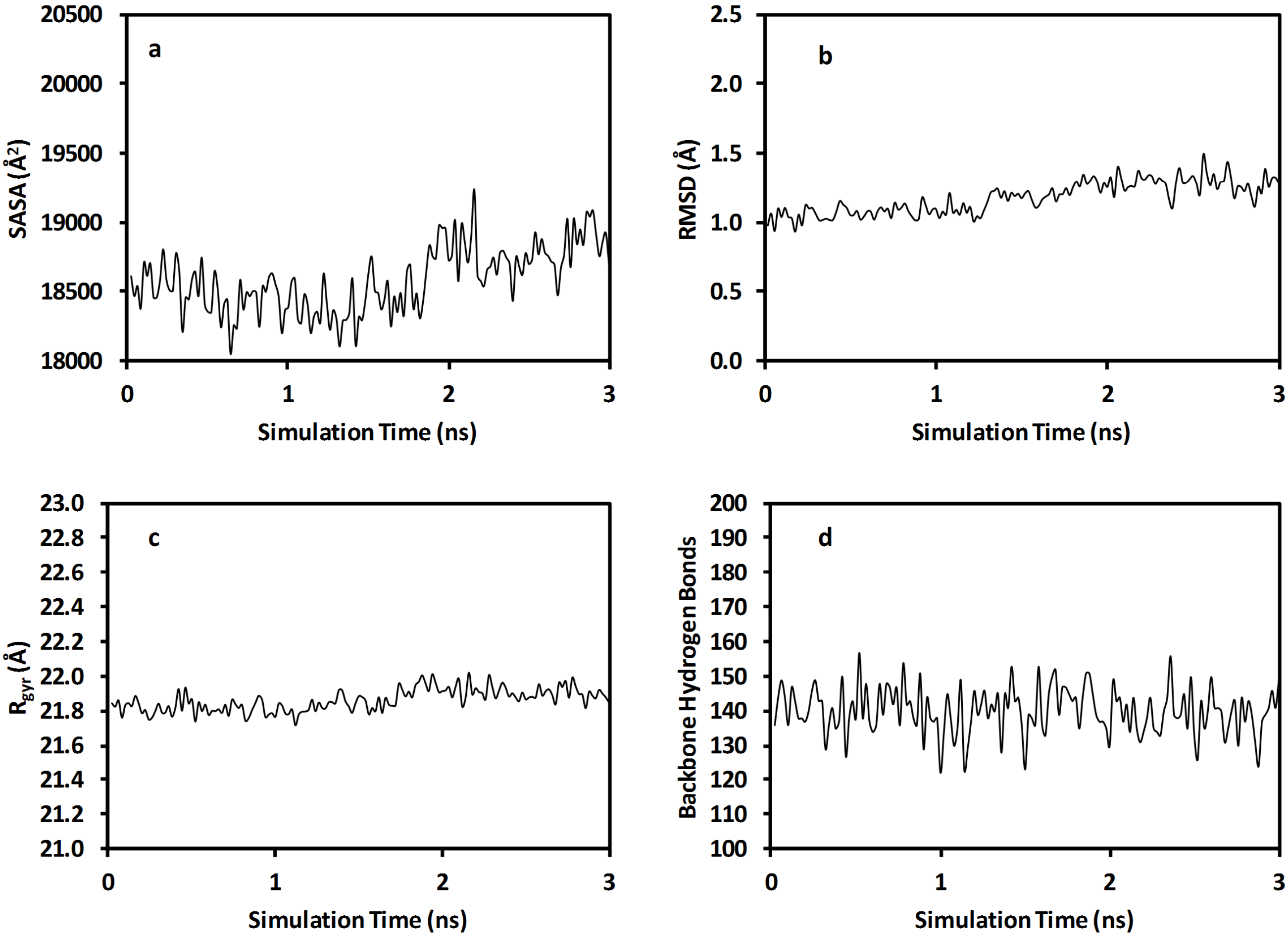

Figure S27. Time series of studied properties from 3 ns NVT simulations. a) SASA, b) Backbone RMSD, c) Radius of Gyration, d) Backbone hydrogen bonds.

NAG_1P2M_KF_300K

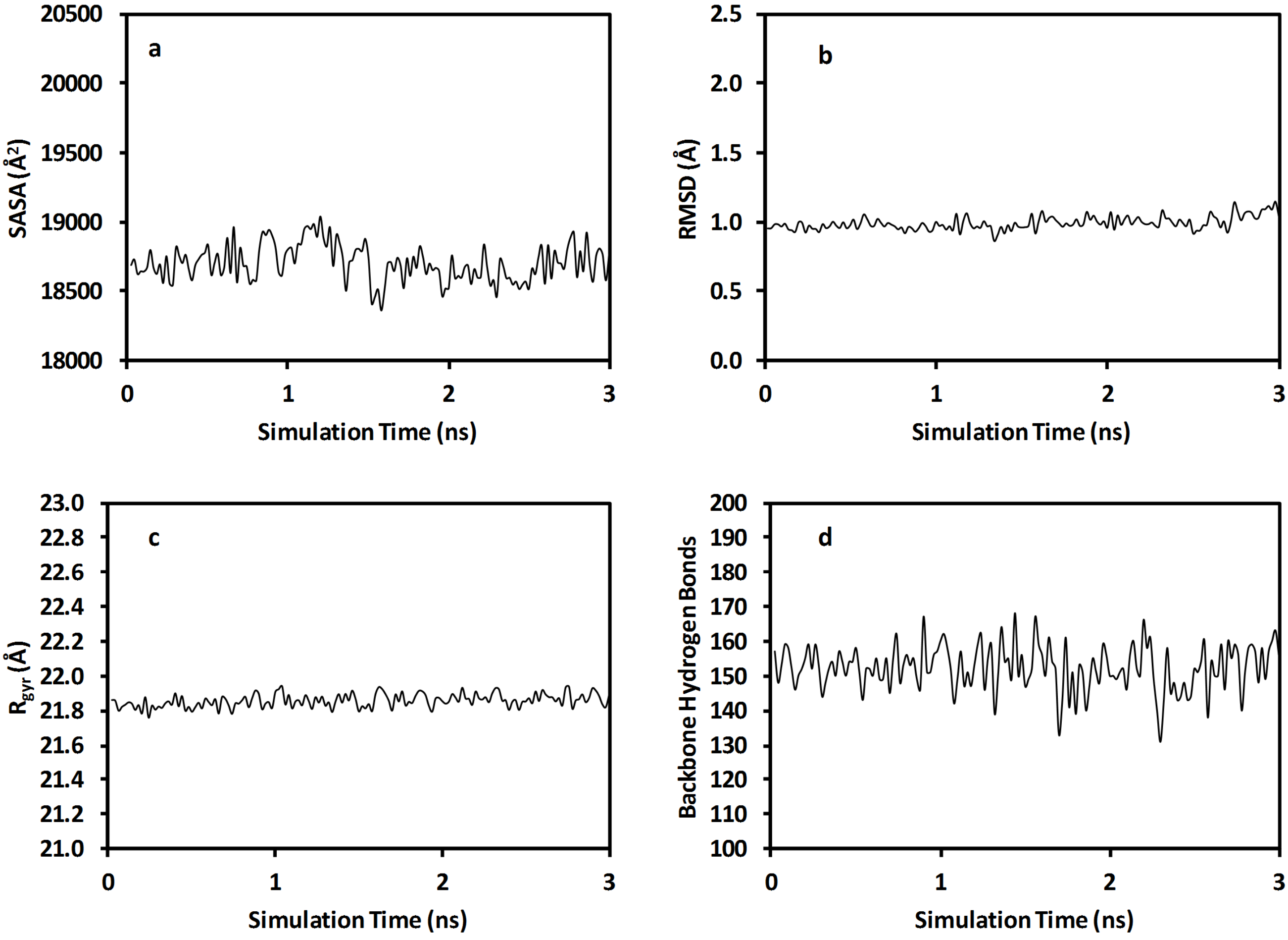

Figure S28. Time series of studied properties from 3 ns NVT simulations. a) SASA, b) Backbone RMSD, c) Radius of Gyration, d) Backbone hydrogen bonds.

NAG_1P2M_KF_350K

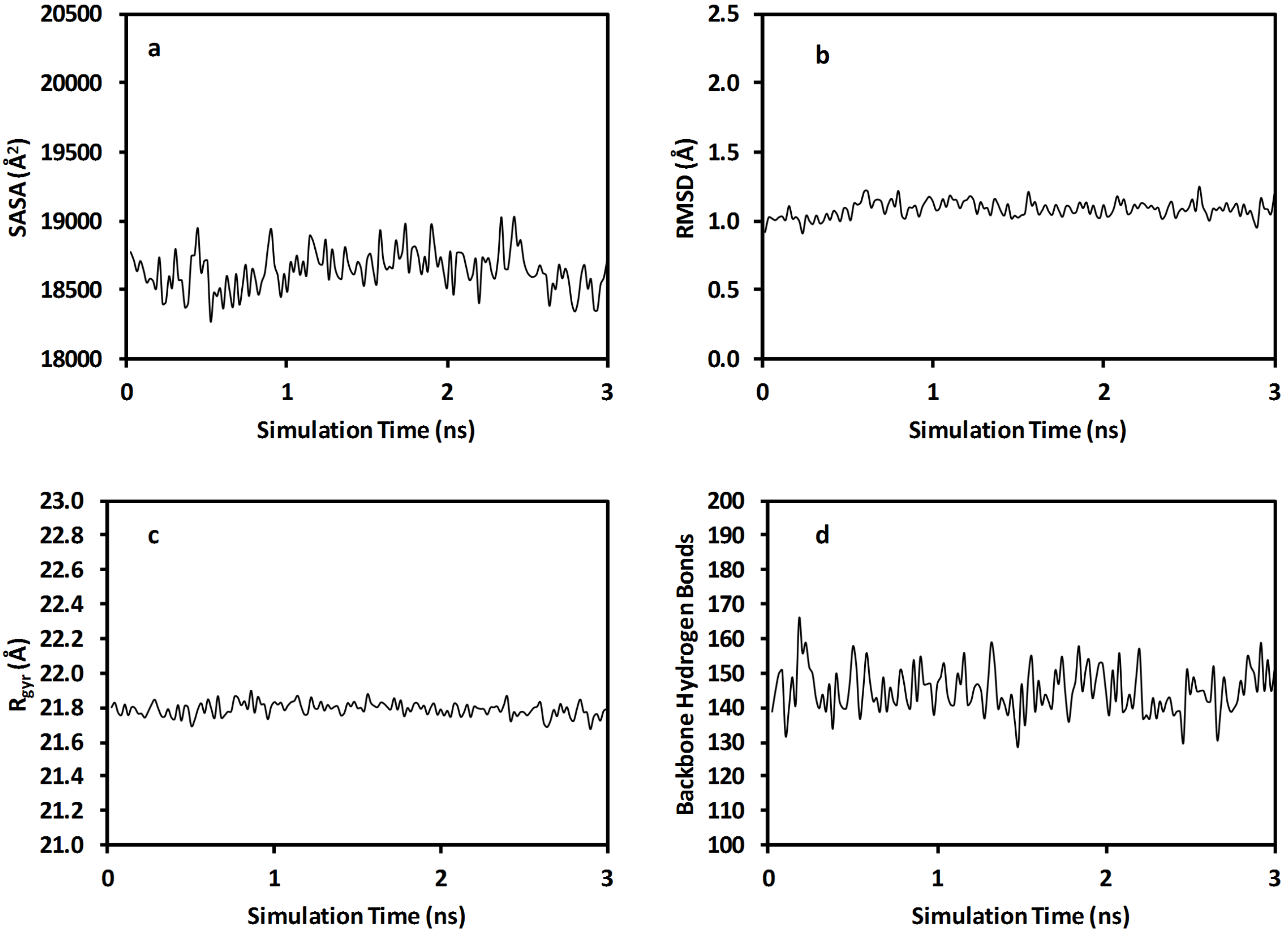

Figure S29. Time series of studied properties from 3 ns NVT simulations. a) SASA, b) Backbone RMSD, c) Radius of Gyration, d) Backbone hydrogen bonds.

NAG_1P2M_KF_400K

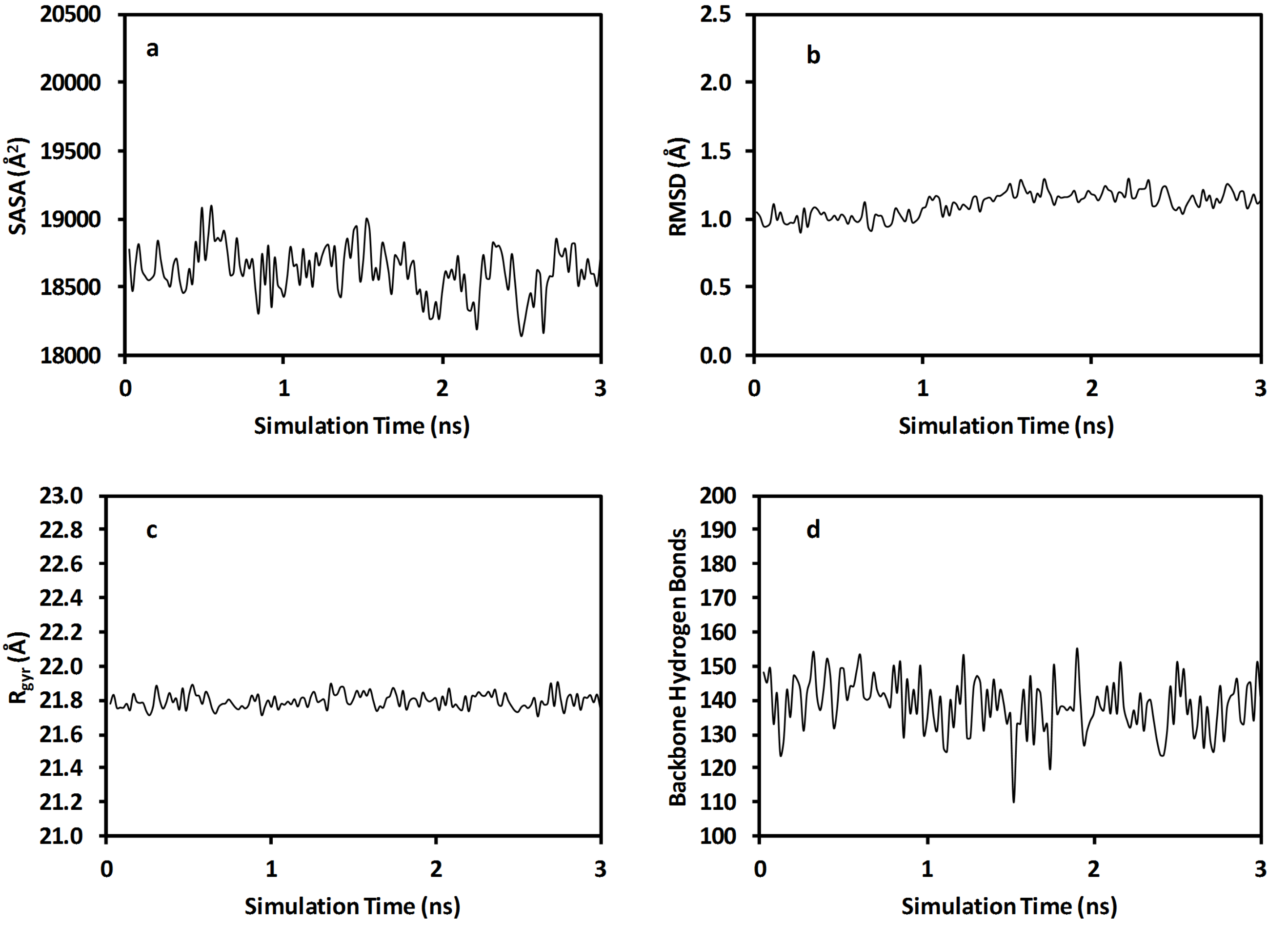

Figure S30. Time series of studied properties from 3 ns NVT simulations. a) SASA, b) Backbone RMSD, c) Radius of Gyration, d) Backbone hydrogen bonds.

noNAG_1P2M_KF_300K

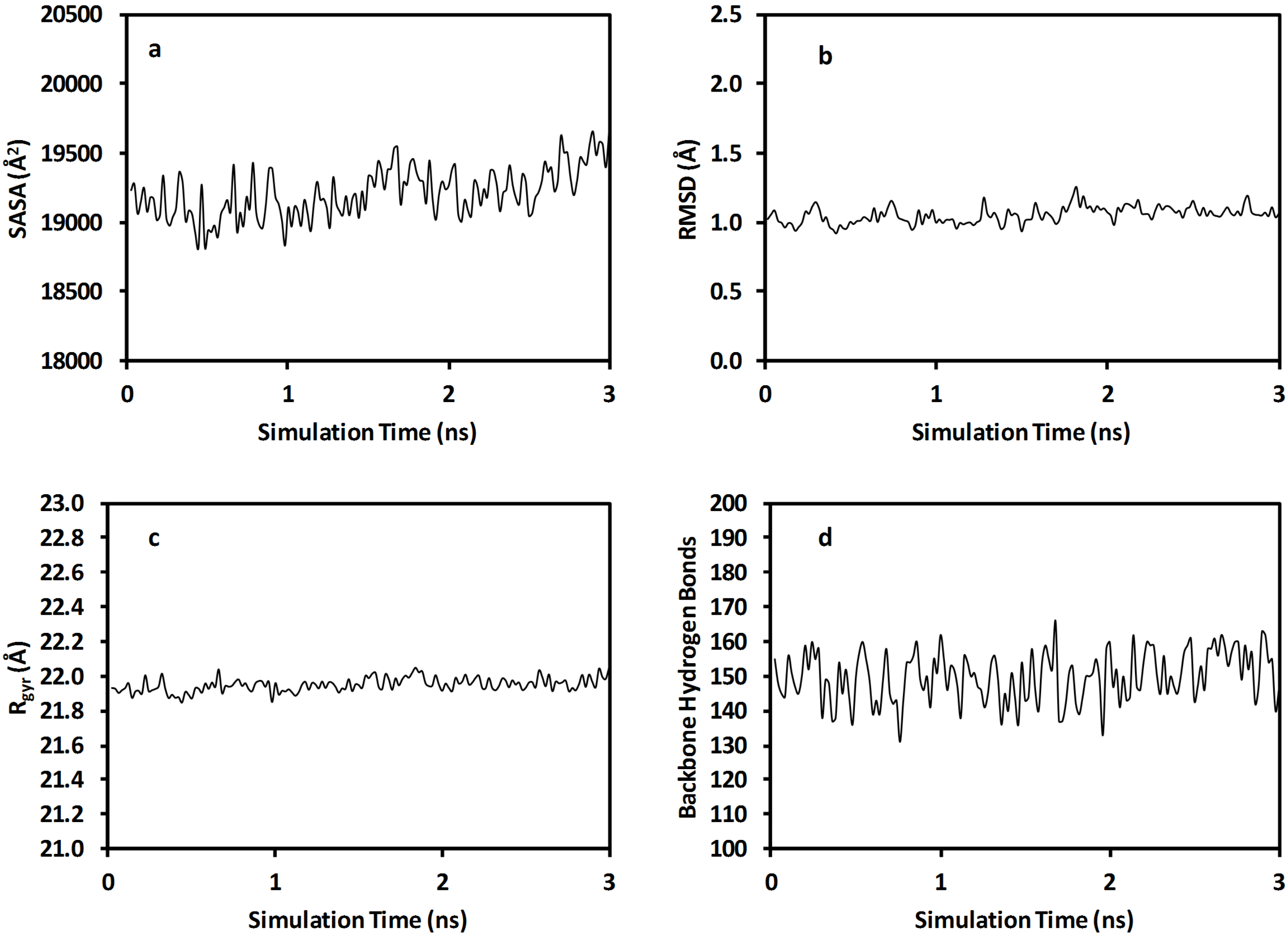

Figure S31. Time series of studied properties from 3 ns NVT simulations. a) SASA, b) Backbone RMSD, c) Radius of Gyration, d) Backbone hydrogen bonds.

noNAG_1P2M_KF_350K

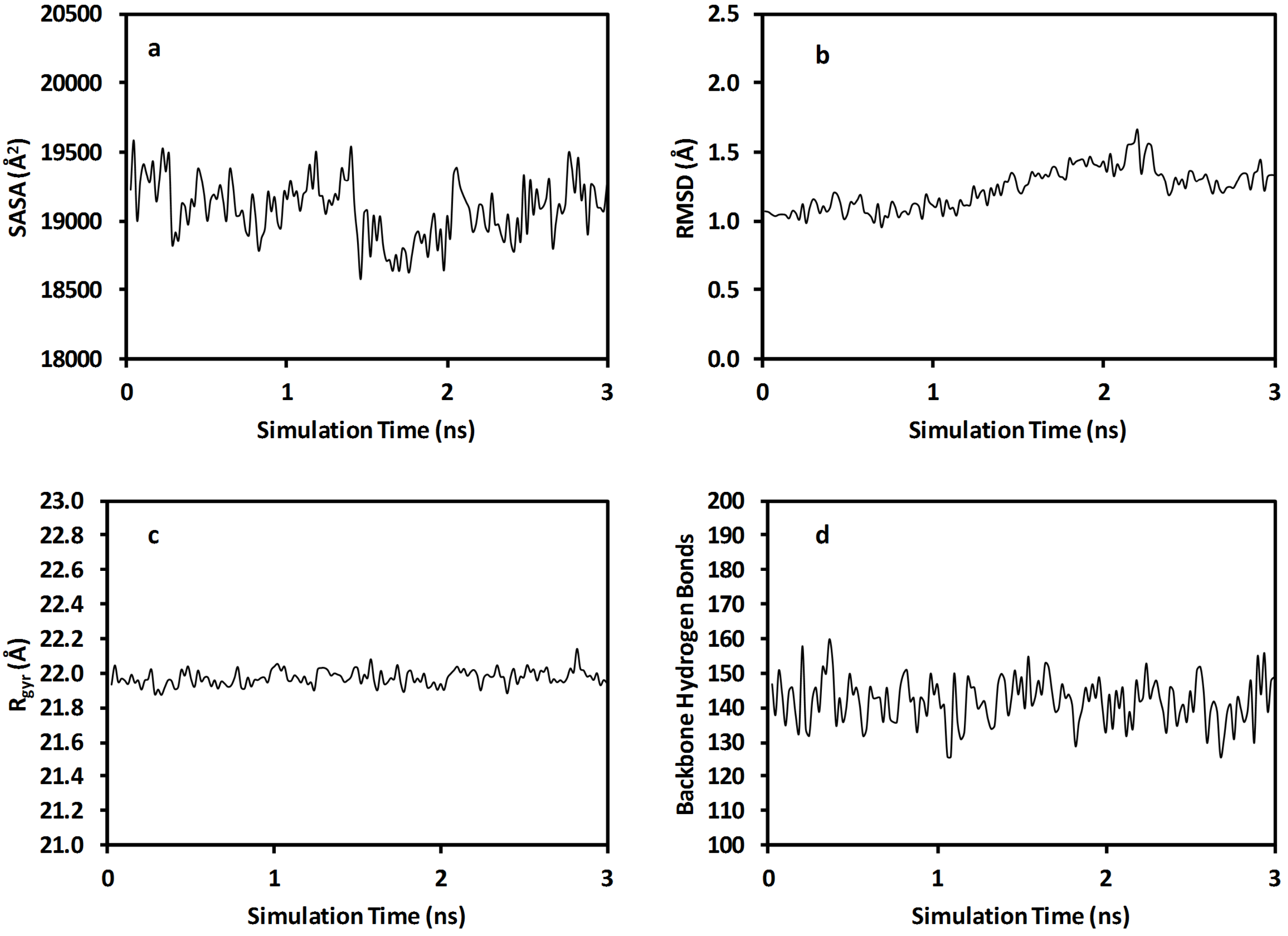

Figure S32. Time series of studied properties from 3 ns NVT simulations. a) SASA, b) Backbone RMSD, c) Radius of Gyration, d) Backbone hydrogen bonds.

noNAG_1P2M_KF_400K

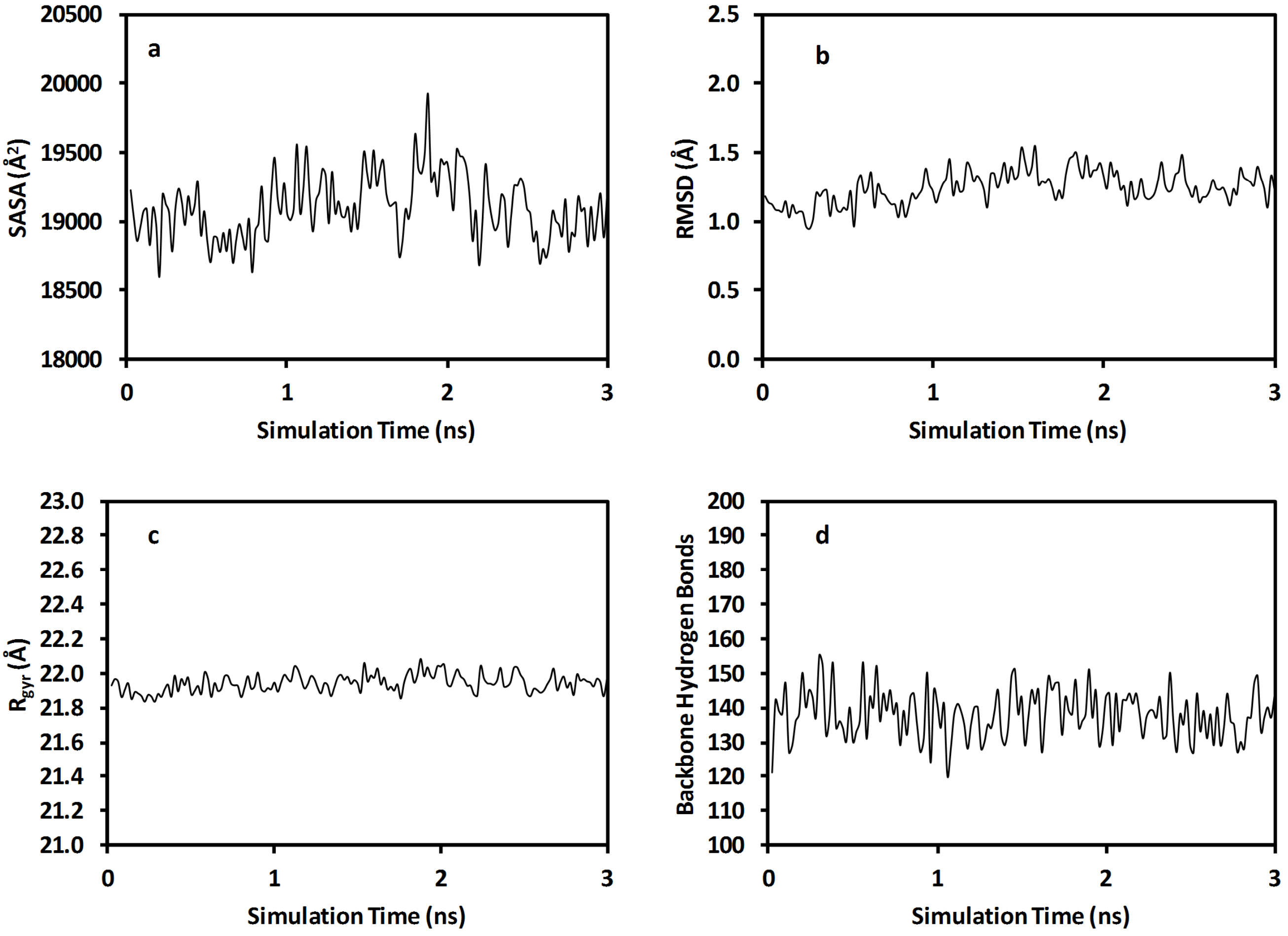

Figure S33. Time series of studied properties from 3 ns NVT simulations. a) SASA, b) Backbone RMSD, c) Radius of Gyration, d) Backbone hydrogen bonds.

Statistics for Molecular Dynamics Simulations

Table S1. Statistics calculated from the last 3 ns NVT MD simulations: Average, Standard Deviation, Minimum and Maximum Values for SASA, R_{gyr}, and backbone RMSD.

	300 K			350 K			400 K		
	SASA	R_{gyr}	RMSD	SASA	R_{gyr}	RMSD	SASA	R_{gyr}	RMSD
NAG_0P0M	18917<br>(130)<br>18572 -<br>19206	21.93<br>(0.04)<br>21.81 –<br>22.01	1.24<br>(0.06)<br>1.08 –<br>1.44	18979<br>(198)<br>18326 -<br>19596	21.91<br>(0.04)<br>21.79 –<br>22.06	1.23<br>(0.07)<br>1.07 –<br>1.47	19149<br>(283)<br>18442 -<br>19882	21.99<br>(0.07)<br>21.80 –<br>22.17	1.61<br>(0.31)<br>1.12 –<br>2.22
noNAG_0P0M	19152<br>(155)<br>18732-<br>19715	21.95<br>(0.04)<br>21.87-<br>22.07	1.22<br>(0.06)<br>1.10-1.41	19271<br>(170)<br>18882 -<br>19728	21.96<br>(0.04)<br>21.87 –<br>22.08	1.37<br>(0.09)<br>1.15 –<br>1.65	19110<br>(236)<br>18474 -<br>19827	21.93<br>(0.05)<br>21.81 –<br>22.06	1.27<br>(0.09)<br>1.07 –<br>1.51
NAG_0P3M_NACL	19096<br>(156)<br>18650 -<br>19473	21.95<br>(0.04)<br>21.83 –<br>22.05	1.25<br>(0.06)<br>1.12 –<br>1.43	18825<br>(172)<br>18354 -<br>19260	21.88<br>(0.04)<br>21.76 –<br>22.00	1.27<br>(0.11)<br>1.05 –<br>1.51	18976<br>(196)<br>18432 -<br>19427	21.91<br>(0.05)<br>21.71 –<br>22.02	1.27<br>(0.09)<br>1.03 –<br>1.52
noNAG_0P3M_NACL	19319<br>(172)<br>18938 –<br>19692	21.98<br>(0.04)<br>21.87 –<br>22.12	1.07<br>(0.06)<br>0.94 –<br>1.23	19149<br>(189)<br>18490 -<br>19545	21.92<br>(0.05)<br>21.78 –<br>22.03	1.10<br>(0.10)<br>0.91 –<br>1.45	19177<br>(210)<br>18645 -<br>19798	21.98<br>(0.05)<br>21.86 –<br>22.12	1.21<br>(0.10)<br>0.91 –<br>1.47
NAG_0P3M_KF	19051<br>(128)<br>18738 -<br>19388	21.94<br>(0.04)<br>21.85 –<br>22.03	1.00<br>(0.05)<br>0.87 –<br>1.13	19088<br>(270)<br>18496 -<br>19916	21.94<br>(0.05)<br>21.76 –<br>22.07	1.02<br>(0.09)<br>0.82 –<br>1.25	19029<br>(183)<br>18625 -<br>19434	21.92<br>(0.05)<br>21.79 –<br>22.06	1.17<br>(0.13)<br>0.89 –<br>1.51
noNAG_0P3M_KF	19188<br>(188)<br>18666 -<br>19747	21.98<br>(0.04)<br>21.88 –<br>22.12	1.20<br>(0.07)<br>1.07 –<br>1.40	19122<br>(187)<br>18630 -<br>19658	21.92<br>(0.04)<br>21.82 –<br>22.07	1.25<br>(0.09)<br>1.10 –<br>1.58	19011<br>(260)<br>18450 -<br>19839	21.96<br>(0.06)<br>21.81 –<br>22.14	1.37<br>(0.13)<br>1.05 –<br>1.73
NAG_1P2M_NACL	19024<br>(184)<br>18615 -	21.94<br>(0.04)<br>21.85 –	1.07<br>(0.05)<br>0.96 –	18948<br>(174)<br>18438 -	21.91<br>(0.04)<br>21.80 –	1.06<br>(0.06)<br>0.91 –	18946<br>(221)<br>18313 -	21.88<br>(0.06)<br>21.73 –	1.18<br>(0.08)<br>0.97 –

	19492	22.04	1.24	19551	22.04	1.25	19471	22.02	1.42
noNAG_1P2M_NACL	18698 (132) 18347 - 18975	21.89 (0.04) 21.80 – 22.01	0.99 (0.04) 0.86 – 1.13	18679 (203) 18213 - 19206	21.87 (0.05) 21.69 – 21.99	1.07 (0.06) 0.93 – 1.24	18583 (229) 18054 - 19231	21.86 (0.06) 21.72 – 22.02	1.18 (0.1) 0.93 – 1.49
NAG_1P2M_KF	18702 (134) 18362 - 19039	21.86 (0.04) 21.76 – 21.94	0.98 (0.04) 0.86 – 1.09	18644 (148) 18274 – 19036	21.79 (0.04) 21.68 – 21.90	1.09 (0.06) 0.91 – 1.25	18622 (179) 18148- 19097	21.79 (0.04) 21.70 – 21.09	1.10 (0.09) 0.90 – 1.29
noNAG_1P2M_KF	19210 (180) 18820 - 19651	21.95 (0.04) 21.85 – 22.05	1.05 (0.06) 0.92 – 1.25	19090 (214) 18581 - 1959	21.97 (0.04) 21.88 – 22.14	1.24 (0.15) 0.96 – 1.66	19099 (228) 18602 - 19931	21.95 (0.05) 21.84 – 22.09	1.24 (0.12) 0.94 – 1.55

Radial Distribution Functions for Backbone Amide-H and Halide Anions in 0.3 M Halide Simulations

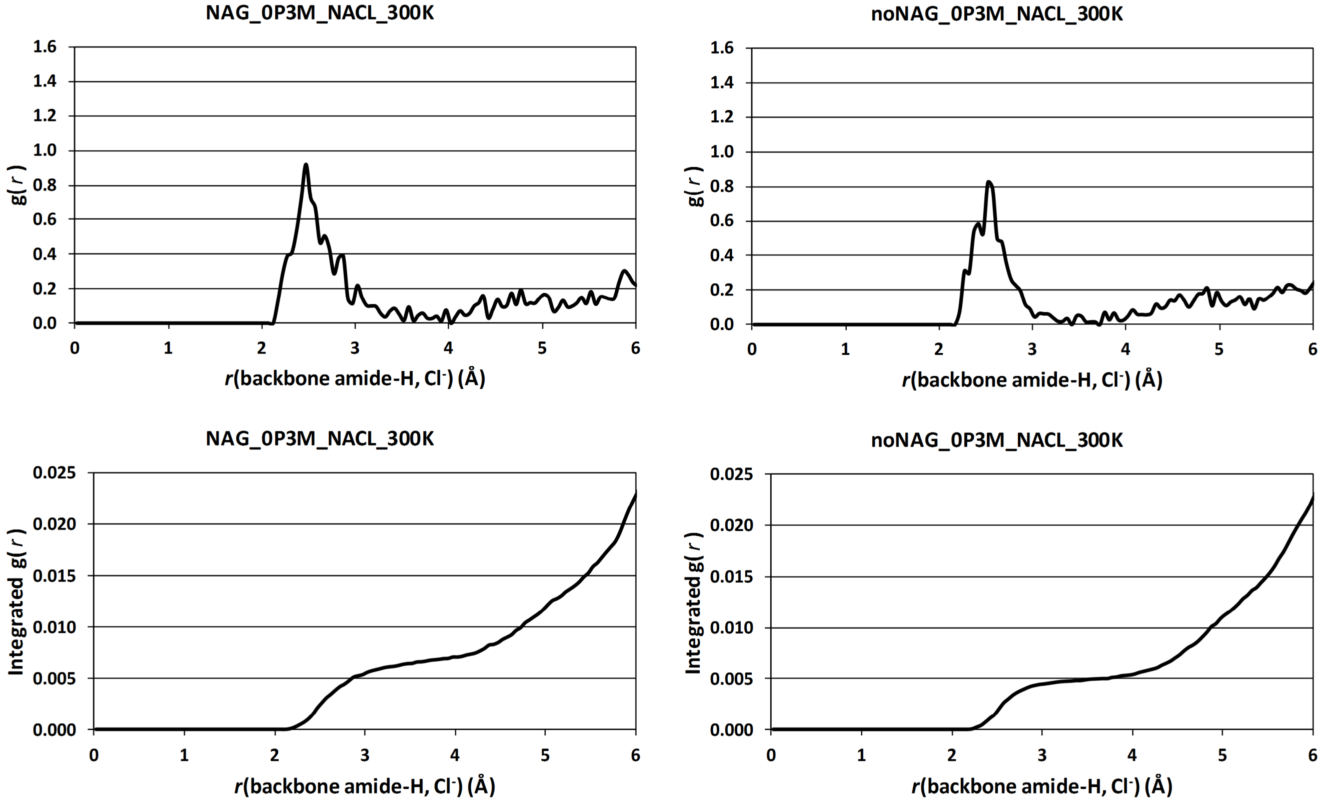

Figure S34. Top panel: Radial distribution function (RDF) for backbone amide-H and halide anion pairs. Bottom panel: Integrated RDF. Left and right panels correspond to simulations with and without glycosylation, respectively.

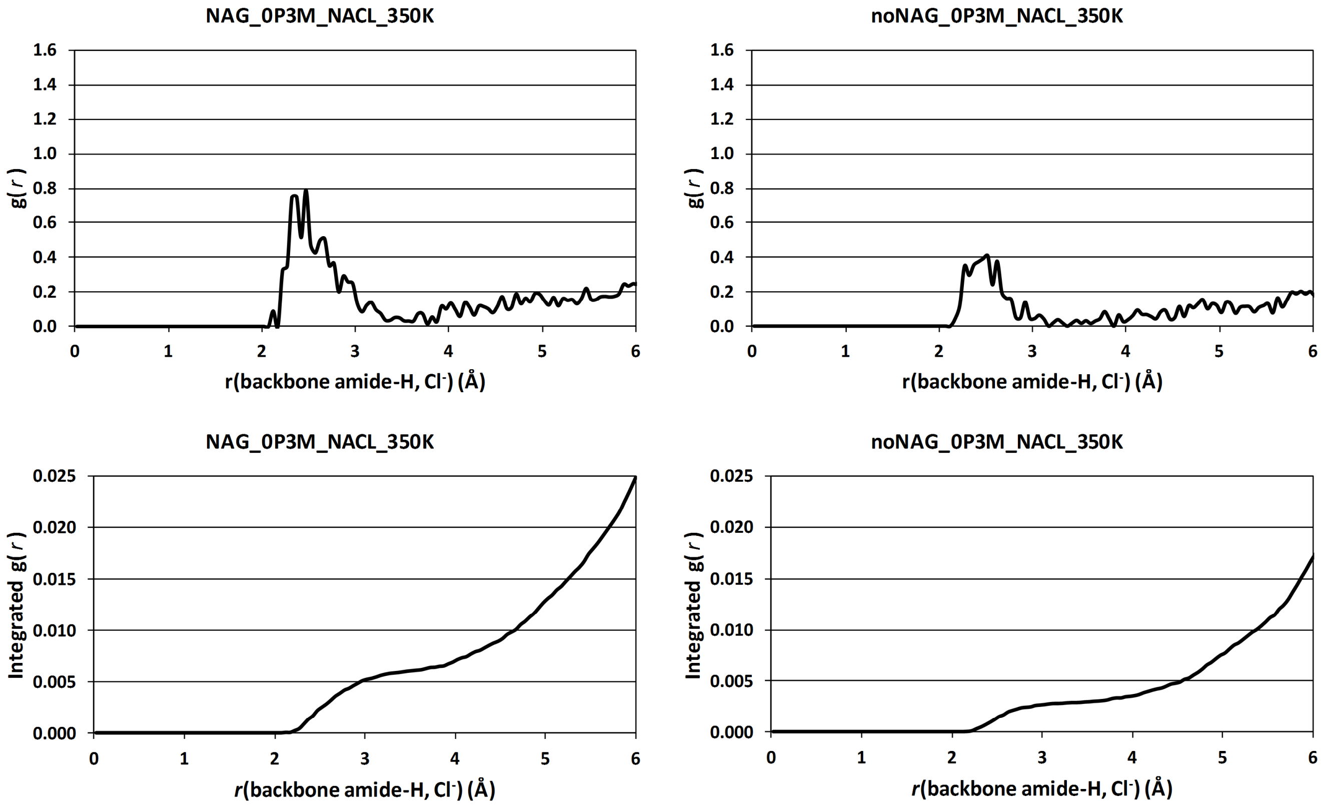

Figure S35. Top panel: Radial distribution function (RDF) for backbone amide-H and halide anion pairs. Bottom panel: Integrated RDF. Left and right panels correspond to simulations with and without glycosylation, respectively.

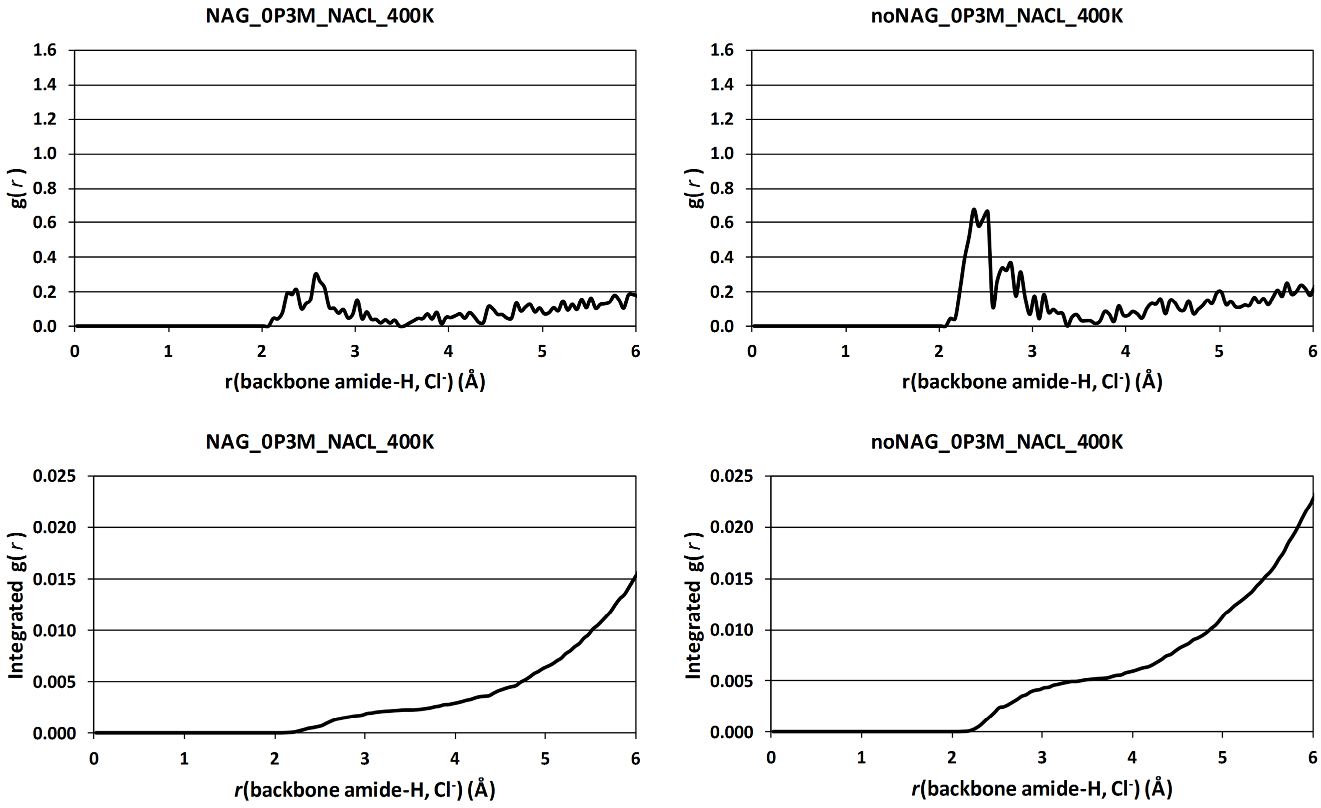

Figure S36. Top panel: Radial distribution function (RDF) for backbone amide-H and halide anion pairs. Bottom panel: Integrated RDF. Left and right panels correspond to simulations with and without glycosylation, respectively.

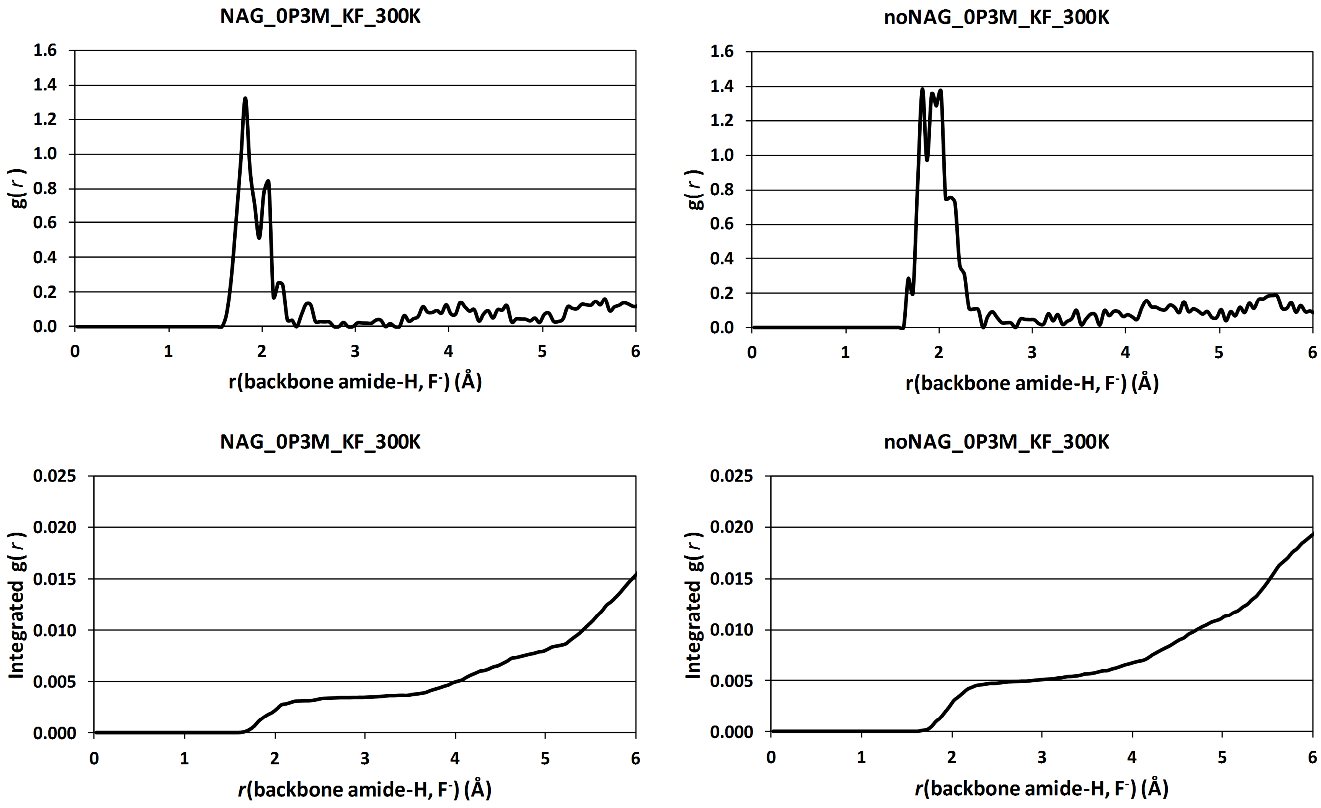

Figure S37. Top panel: Radial distribution function (RDF) for backbone amide-H and halide anion pairs. Bottom panel: Integrated RDF. Left and right panels correspond to simulations with and without glycosylation, respectively.

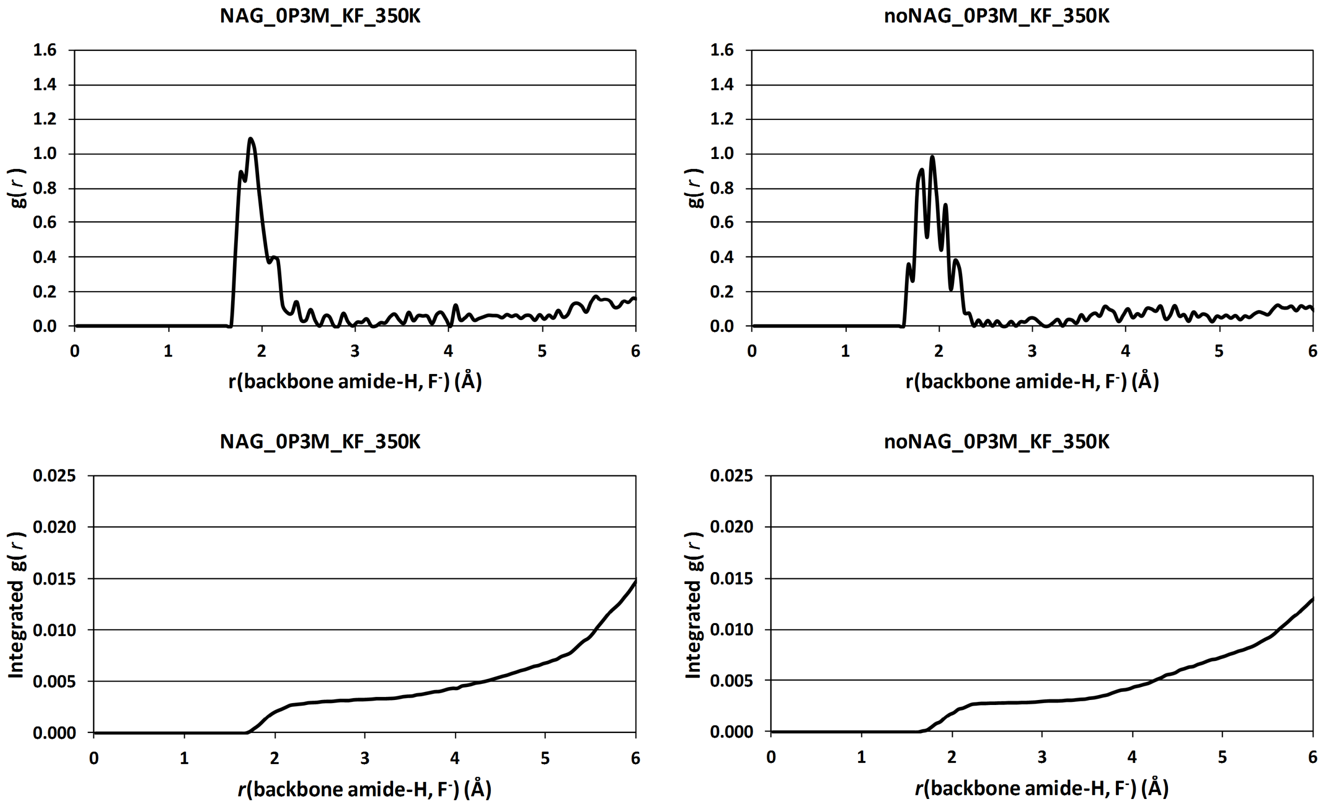

Figure S38. Top panel: Radial distribution function (RDF) for backbone amide-H and halide anion pairs. Bottom panel: Integrated RDF. Left and right panels correspond to simulations with and without glycosylation, respectively.

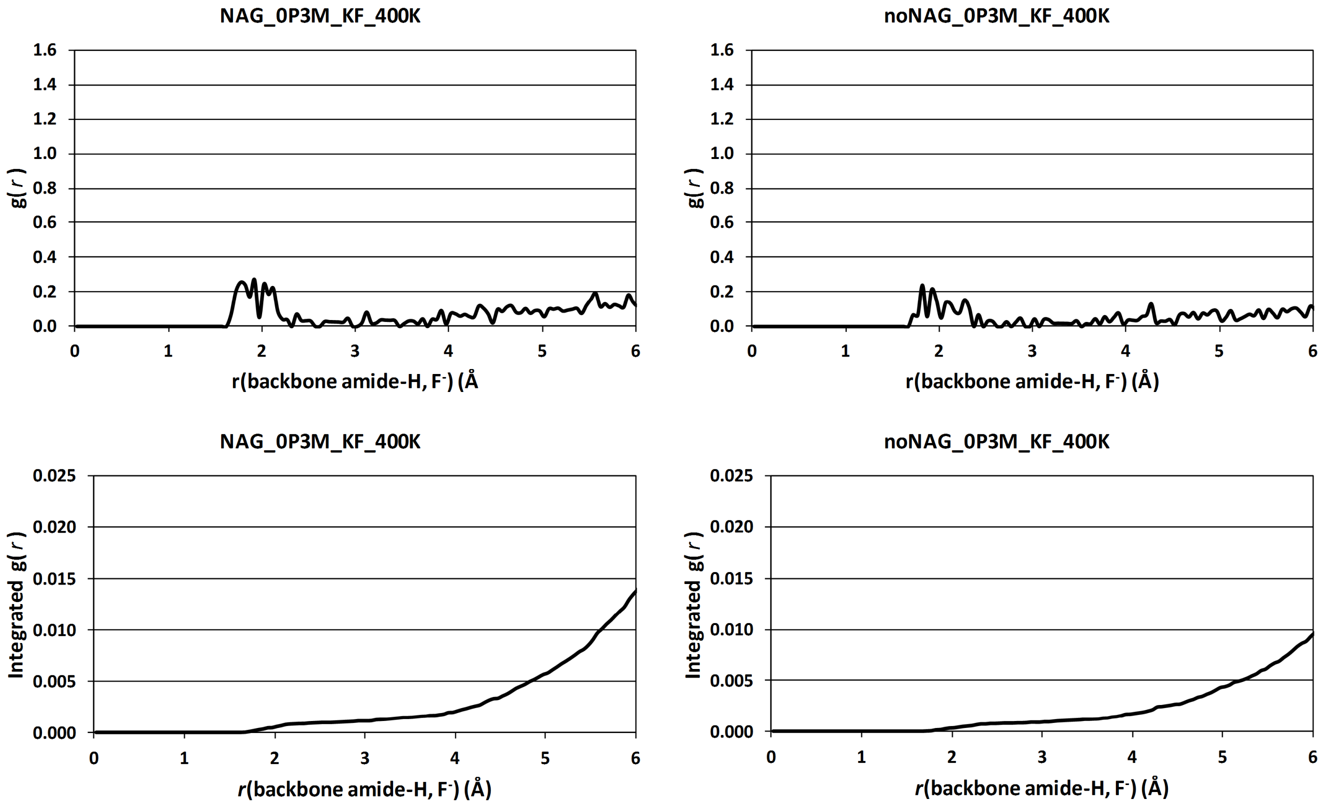

Figure S39. Top panel: Radial distribution function (RDF) for backbone amide-H and halide anion pairs. Bottom panel: Integrated RDF. Left and right panels correspond to simulations with and without glycosylation, respectively.

Persistence of Salt Bridges

Table S2. Glycosylated TvLαin NaCl background: Salt bridges found in 3 ns NVT simulations and their persistence (Cons. %) in percentage of 100 frames sampled from 2 last ns of simulations. The simulation average distance (r) in Å between the center of mass of interacting charged groups is also given. Color codes indicate stable salt bridges found in the majority of simulations.

	**0 M, 300 K**		**0 M, 350 K**		**0 M, 400 K**	
**Salt Bridge**	**Cons. (%)**	**r (Å)**	**Cons. (%)**	**r (Å)**	**Cons. (%)**	**r (Å)**
ASP128-LYS40	94.0	3.1	96.0	3.0	94.0	3.0
ASP138-ARG195			82.0	3.4	72.0	3.4
ASP214-ARG260	100.0	3.3	100.0	3.3	96.0	3.3
ASP224-ARG423	98.0	3.3	95.0	3.3	96.0	3.4
ASP42-LYS39	94.0	2.9	96.0	2.9		
ASP424-ARG243	100.0	3.3	98.0	3.3	95.0	3.3
ASP96-ARG43	98.0	3.3	90.0	3.3	90.0	3.3
GLU288-ARG176	72.0	3.3	87.0	3.3		
	**0.3 M NaCl, 300 K**		**0.3 M NaCl, 350 K**		**0.3 M NaCl, 400 K**	
	**Cons. (%)**	**r (Å)**	**Cons. (%)**	**r (Å)**	**Cons. (%)**	**r (Å)**
ASP118-ARG22	98.0	3.3			72.0	3.3
ASP128-LYS40	89.0	3.2	86.0	3.1	94.0	3.1
ASP138-ARG195					83.0	3.3
ASP140-ARG199			84.0	3.4	81.0	3.4
ASP214-ARG260	99.0	3.3	98.0	3.3	97.0	3.3
ASP224-ARG423	95.0	3.4	90.0	3.3	89.0	3.4
ASP42-LYS39	65.0	3.1				
ASP424-ARG243	100.0	3.3	99.0	3.3	96.0	3.3
ASP96-ARG43	78.0	3.3	81.0	3.3		
ASP486-LYS482					61.0	2.8
GLU288-ARG176					75.0	3.3
	**1.2 M NaCl, 300 K**		**1.2 M NaCl, 350 K**		**1.2 M NaCl, 400 K**	
	**Cons. (%)**	**r (Å)**	**Cons. (%)**	**r (Å)**	**Cons. (%)**	**r (Å)**
ASP118-ARG22			77.0	3.3	93.0	3.3
ASP128-LYS40	89.0	3.1	89.0	3.0	70.0	3.2
ASP131-ARG197			68.0	3.3		
ASP214-ARG260	100.0	3.3	98.0	3.3	97.0	3.3
ASP224-ARG423	98.0	3.4	89.0	3.4	91.0	3.4
ASP42-LYS39			98.0	2.9	69.0	2.9
ASP424-ARG243	99.0	3.3	100.0	3.3	98.0	3.3
ASP486-LYS482			87.0	2.8	51.0	2.8
ASP96-ARG43	95.0	3.3	91.0	3.3	86.0	3.3
GLU288-ARG176	83.0	3.3	67.0	3.3	62.0	3.3
GLU381-ARG440			67.0	3.3		
GLU460-ARG157					76.0	3.3
ASP138-ARG195					76.0	3.4

Table S3. Non-glycosylated TvLαin NaCl background: Salt bridges found in 3 ns NVT simulations and their persistence (Cons. %) in percentage of 100 frames sampled from 2 last ns of simulations. The simulation average distance (r) in Å between the center of mass of interacting charged groups is also given. Color codes indicate stable salt bridges found in the majority of simulations.

Salt Bridge	0 M, 300 K		0 M, 350 K		0 M, 400 K	
	Cons. (%)	r (Å)	Cons. (%)	r (Å)	Cons. (%)	r (Å)
ASP128-LYS40	68.0	3.2	76.0	3.1	83.0	3.1
ASP138-ARG195	80.0	3.4	71.0	3.4	78.0	3.3
ASP140-ARG199	88.0	3.4	92.0	3.4		
ASP214-ARG260	98.0	3.3	99.0	3.3	95.0	3.3
ASP224-ARG423	92.0	3.4	95.0	3.4	89.0	3.4
ASP42-LYS39	97.0	3.0	53.0	3.0	56.0	2.9
ASP424-ARG243	99.0	3.3	99.0	3.3	96.0	3.3
ASP486-LYS482	99.0	2.7				
ASP96-ARG43	89.0	3.3	89.0	3.3	88.0	3.3
GLU288-ARG176	64.0	3.3	76.0	3.3	60.0	3.3
GLU460-ARG157	57.0	3.4				
	**0.3 M NaCl, 300 K**		**0.3 M NaCl, 350 K**		**0.3 M NaCl, 400 K**	
	Cons. (%)	r (Å)	Cons. (%)	r (Å)	Cons. (%)	r (Å)
ASP128-LYS40	85.0	3.1	88.0	3.1	66.0	3.1
ASP138-ARG195						
ASP140-ARG199	66.0	3.4	83.0	3.4	53.0	3.4
ASP214-ARG260	100.0	3.3	99.0	3.3	98.0	3.3
ASP224-ARG423	92.0	3.4	94.0	3.4	94.0	3.4
ASP42-LYS39	95.0	2.9	65.0	3.0	84.0	3.3
ASP424-ARG243	99.0	3.3	96.0	3.3		
ASP486-LYS482					78.0	2.8
ASP96-ARG43	97.0	3.3	92.0	3.3	87.0	3.3
GLU288-ARG176			75.0	3.3	60.0	3.3
	**1.2 M NaCl, 300 K**		**1.2 M NaCl, 350 K**		**1.2 M NaCl, 400 K**	
	Cons. (%)	r (Å)	Cons. (%)	r (Å)	Cons. (%)	r (Å)
ASP128-LYS40	93.0	3.1	89.0	3.2	82.0	3.1
ASP138-ARG195						
ASP140-ARG199	88.0	3.3				
ASP214-ARG260	100.0	3.3	99.0	3.3	99.0	3.2
ASP224-ARG423	87.0	3.4	93.0	3.4	97.0	3.4
ASP42-LYS39						
ASP424-ARG243	100.0	3.3	85.0	3.3	93.0	3.3
ASP486-LYS482			54.0	2.8		
ASP96-ARG43	82.0	3.3	85.0	3.3	85.0	3.3
GLU288-ARG176	83.0	3.3	60.0	3.3	52.0	3.3

Table S4. Glycosylated TvLα in KF background: Salt bridges found in 3 ns NVT simulations and their persistence (Cons. %) in percentage of 100 frames sampled from 2 last ns of simulations. The simulation average distance (r) in Å between the center of mass of interacting charged groups is also given. Color codes indicate stable salt bridges found in the majority of simulations.

Salt Bridge	0.3 M KF, 300 K		0.3 M KF, 350 K		0.3 M KF, 400 K	
	Cons. (%)	r (Å)	Cons. (%)	r (Å)	Cons. (%)	r (Å)
ASP128-LYS130			97.0	3.0		
ASP128-LYS40	94.0	3.1	78.0	3.3	92.0	3.0
ASP138-ARG195			87.0	3.4	61.0	3.4
ASP214-ARG260	99.0	3.3	99.0	3.3	99.0	3.3
ASP224-ARG423	94.0	3.4	88.0	3.4	90.0	3.4
ASP424-ARG243	98.0	3.3	99.0	3.3	98.0	3.3
ASP486-LYS482	88.0	2.8	55.0	2.8		
ASP96-ARG43	85.0	3.3	94.0	3.3	85.0	3.3
GLU288-ARG176			65.0	3.3	68.0	3.3
ASP140-ARG199					90.0	3.4
	1.2 M KF, 300 K		1.2 M KF, 350 K		1.2 M KF, 400 K	
	Cons. (%)	r (Å)	Cons. (%)	r (Å)	Cons. (%)	r (Å)
ASP128-LYS40	51.4	3.0	99.0	2.9	90.0	3.0
ASP214-ARG260	100.0	3.3	98.0	3.3	99.0	3.3
ASP224-ARG423	94.4	3.4	99.0	3.3	90.0	3.4
ASP424-ARG243	98.6	3.3	98.0	3.3	98.0	3.3
ASP96-ARG43	90.3	3.3	94.0	3.3	85.0	3.3
GLU288-ARG176	77.8	3.3	74.0	3.3	56.0	3.3
GLU460-ARG161	54.2	3.3				
ASP486-LYS482	66.7	2.8				
ASP138-ARG195					64.0	3.4
ASP140-ARG199					69.0	3.4

Table S5. Non-glycosylated TvLα in KF background: Salt bridges found in 3 ns NVT simulations and their persistence (Cons. %) in percentage of 100 frames sampled from 2 last ns of simulations. The simulation average distance (r) in Å between the center of mass of interacting charged groups is also given. Color codes indicate stable salt bridges found in the majority of simulations.

**Salt Bridge**	**0.3 M KF, 300 K**		**0.3 M KF, 350 K**		**0.3 M KF, 400 K**	
	**Cons. (%)**	**r (Å)**	**Cons. (%)**	**r (Å)**	**Cons. (%)**	**r (Å)**
ASP128-LYS40	81.0	3.2	83.0	3.1	93.0	3.0
ASP138-ARG195	85.0	3.4	82.0	3.4	84.0	3.4
ASP214-ARG260	99.0	3.3	95.0	3.3	98.0	3.3
ASP224-ARG423	92.0	3.3	94.0	3.3	91.0	3.4
ASP42-LYS39	96.0	3.0	81.0	3.0	65.0	2.9
ASP424-ARG243	99.0	3.3	99.0	3.3	91.0	3.3
ASP96-ARG43	93.0	3.3	93.0	3.3		
GLU288-ARG176	89.0	3.3	71.0	3.3	71.0	3.3
ASP486-LYS482			91.0	2.8		
ASP140-ARG199					63.0	3.4
	**1.2 M KF, 300 K**		**1.2 M KF, 350 K**		**1.2 M KF, 400 K**	
	**Cons. (%)**	**r (Å)**	**Cons. (%)**	**r (Å)**	**Cons. (%)**	**r (Å)**
ASP128-LYS40	92.0	3.0	88.0	3.1	86.0	3.0
ASP140-ARG199	90.0	3.4	95.0	3.4	72.0	3.4
ASP214-ARG260	100.0	3.3	97.0	3.3	99.0	3.3
ASP224-ARG423	88.0	3.4	90.0	3.4	83.0	3.4
ASP424-ARG243	100.0	3.3	98.0	3.3	96.0	3.3
ASP486-LYS482	90.0	2.8	85.0	2.8	69.0	2.8
ASP96-ARG43	98.0	3.3	95.0	3.3	88.0	3.3
GLU288-ARG176	78.0	3.3	77.0	3.3	71.0	3.3
ASP42-LYS39			95.0	2.9		

B-factor Plots for 3 ns NVT MD Simulations

NAG_0P0M

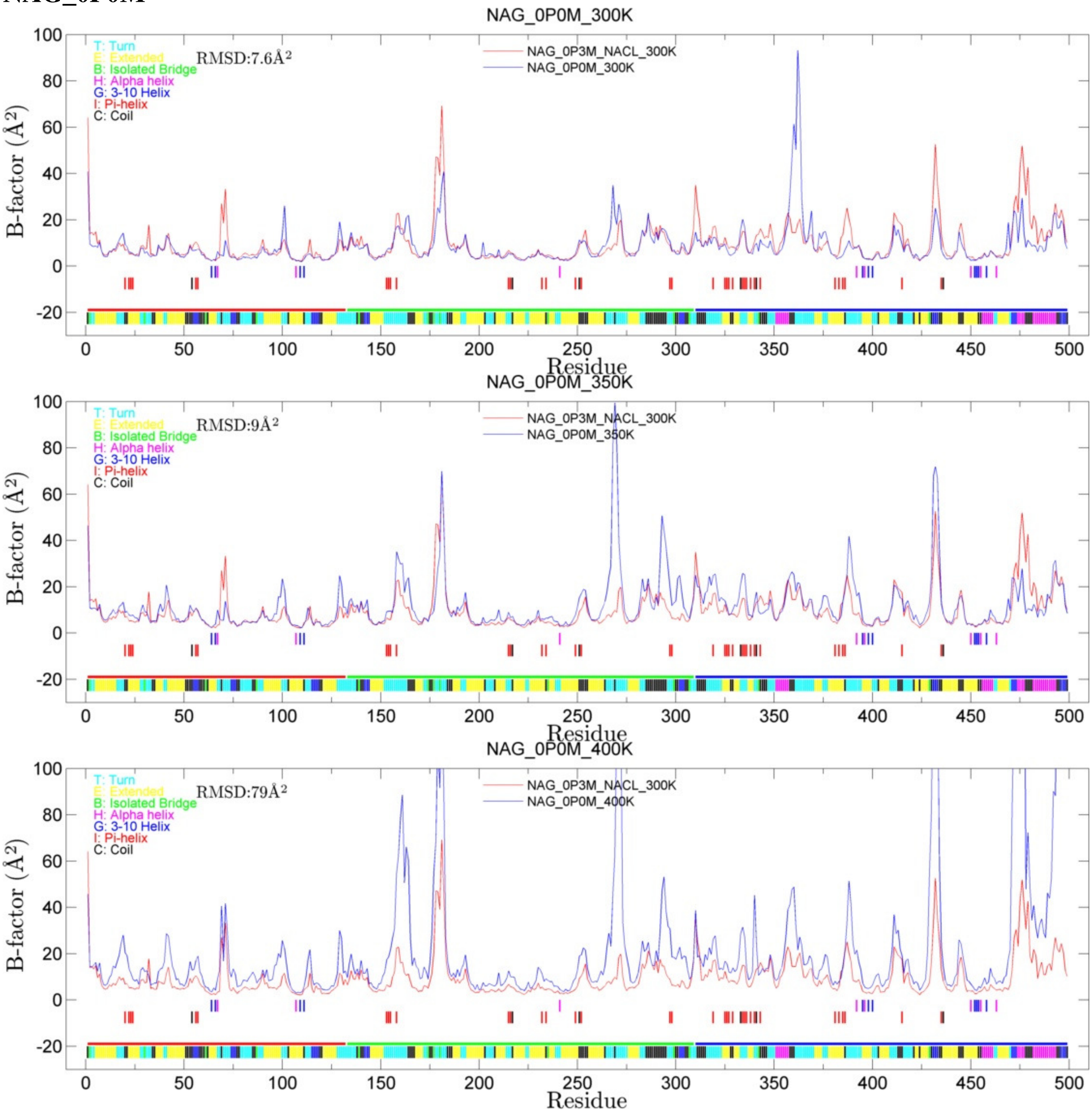

Figure S40. Overlay of reference simulation (red) and current simulation (blue) B-factors for TvLα. The bottom bar indicates secondary structure, with color codes in the upper-left legend. The horizontal three-colored line immediately above the secondary structure bar denotes the three laccase domains (D1: Red, D2: Green, D3: Blue). Above the domain line, short black vertical lines indicate NAG-positions, and red lines denote residues initially 4.5 Å from NAG. Blue and magenta lines indicate residues directly coordinating Cu and immediate structural neighbors of Cu-binding residues, respectively.

noNAG_0P0M

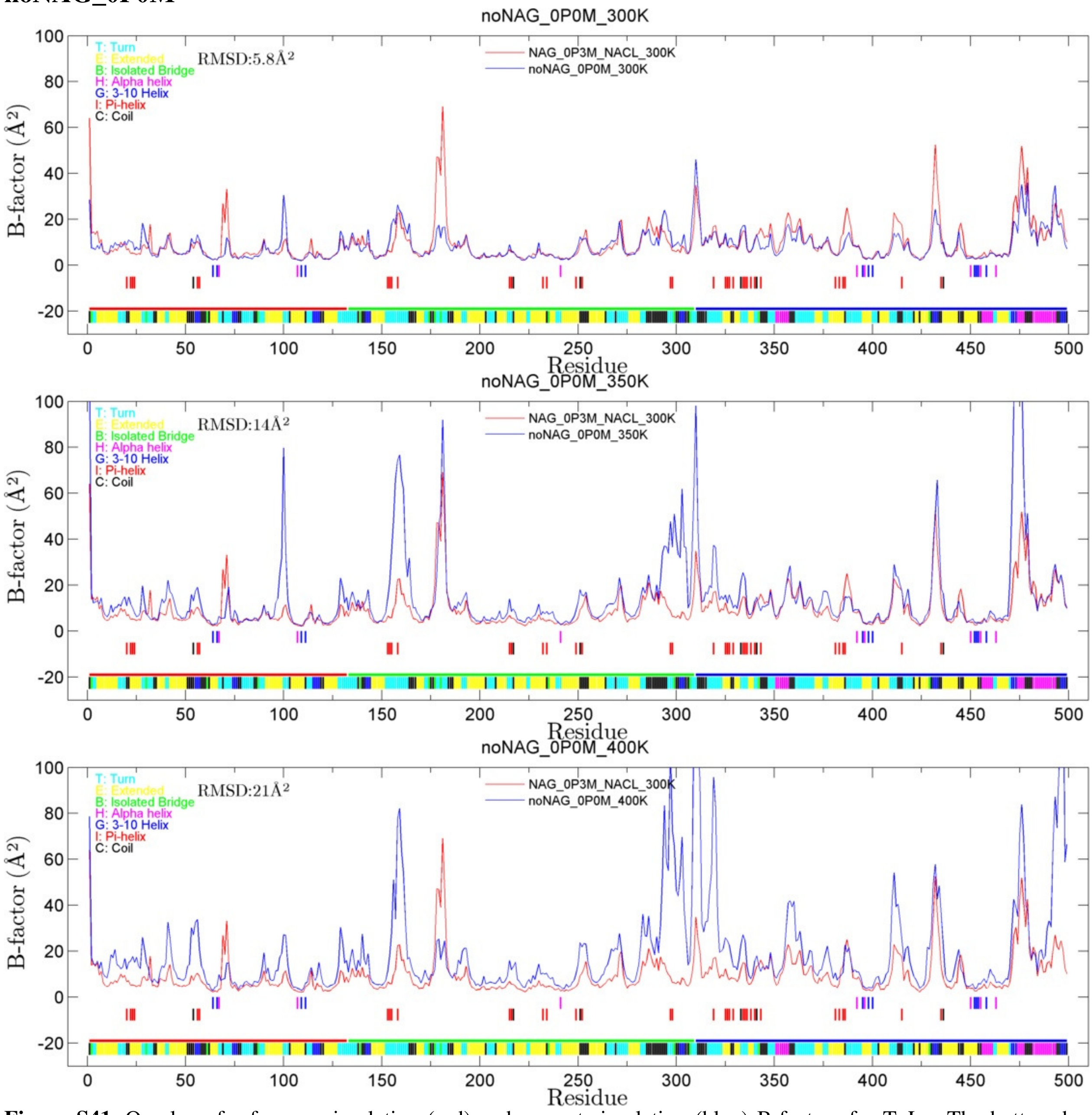

Figure S41. Overlay of reference simulation (red) and current simulation (blue) B-factors for TvLα. The bottom bar indicates secondary structure, with color codes in the upper-left legend. The horizontal three-colored line immediately above the secondary structure bar denotes the three laccase domains (D1: Red, D2: Green, D3: Blue). Above the domain line, short black vertical lines indicate NAG-positions, and red lines denote residues initially 4.5 Å from NAG. Blue and magenta lines indicate residues directly coordinating Cu and immediate structural neighbors of Cu-binding residues, respectively.

NAG_0P3M_NACL

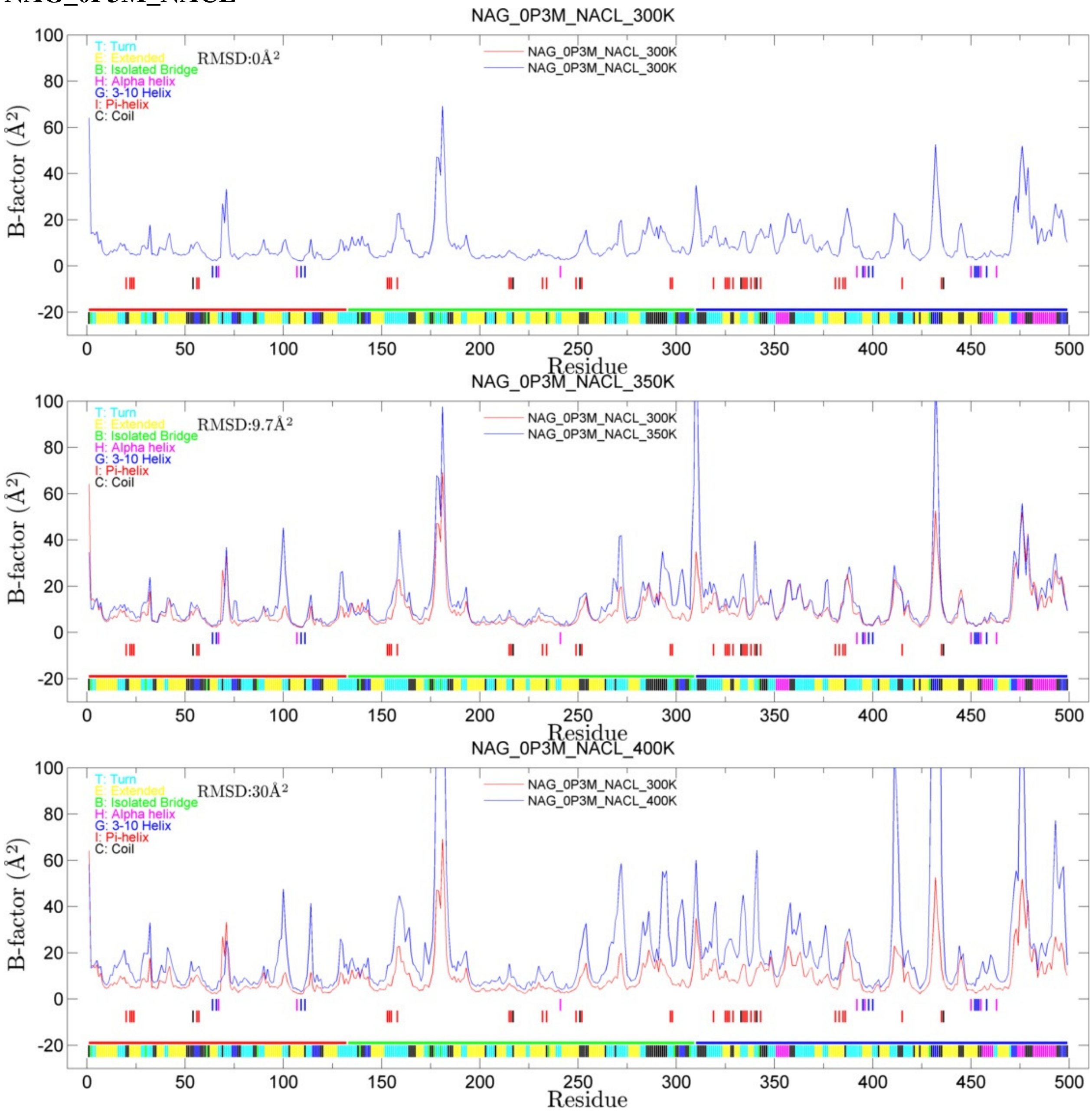

Figure S42. Overlay of reference simulation (red) and current simulation (blue) B-factors for TvLα. The bottom bar indicates secondary structure, with color codes in the upper-left legend. The horizontal three-colored line immediately above the secondary structure bar denotes the three laccase domains (D1: Red, D2: Green, D3: Blue). Above the domain line, short black vertical lines indicate NAG-positions, and red lines denote residues initially 4.5 Å from NAG. Blue and magenta lines indicate residues directly coordinating Cu and immediate structural neighbors of Cu-binding residues, respectively.

noNAG_0P3M_NACL

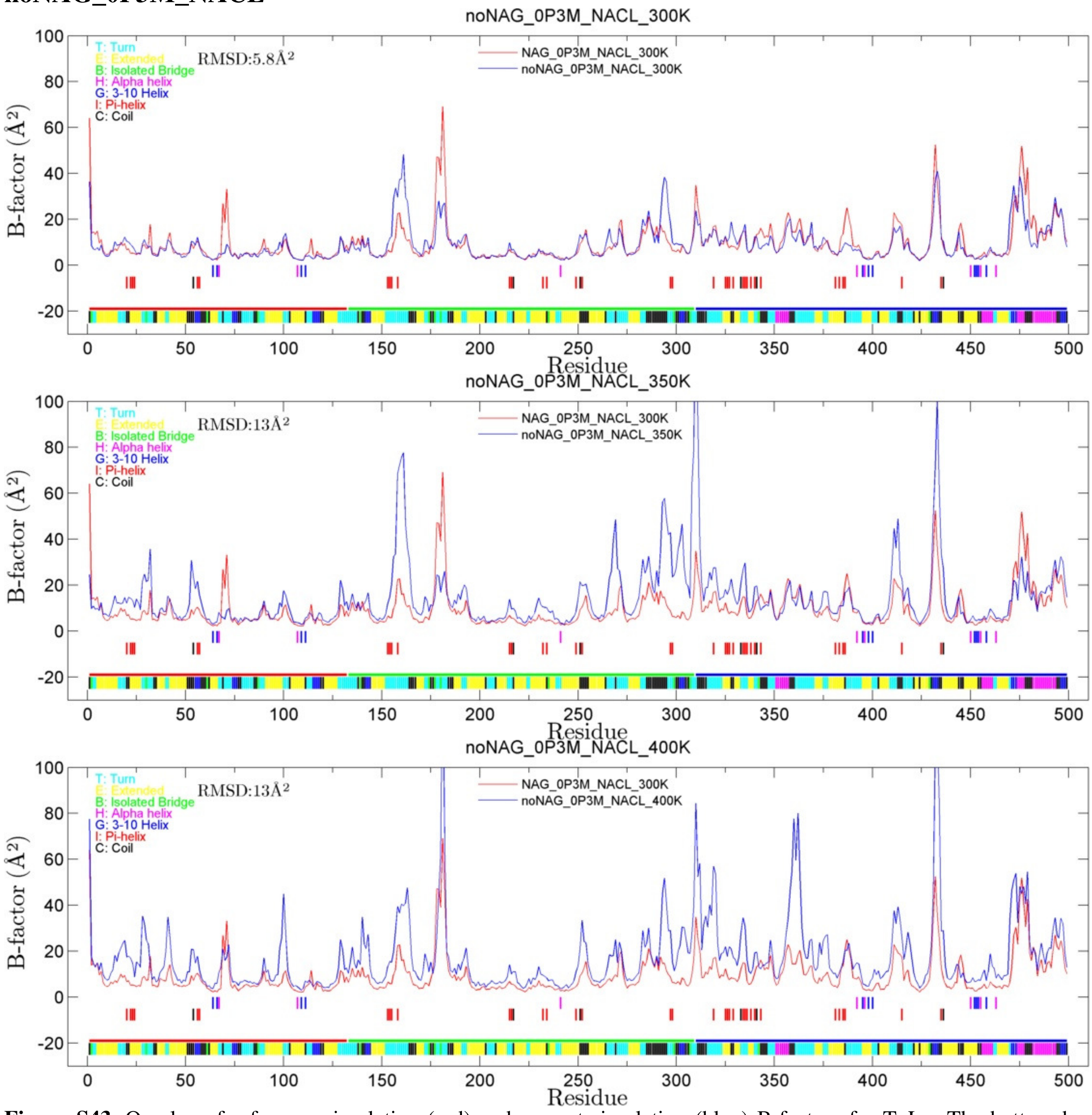

Figure S43. Overlay of reference simulation (red) and current simulation (blue) B-factors for TvLα. The bottom bar indicates secondary structure, with color codes in the upper-left legend. The horizontal three-colored line immediately above the secondary structure bar denotes the three laccase domains (D1: Red, D2: Green, D3: Blue). Above the domain line, short black vertical lines indicate NAG-positions, and red lines denote residues initially 4.5 Å from NAG. Blue and magenta lines indicate residues directly coordinating Cu and immediate structural neighbors of Cu-binding residues, respectively.

NAG_0P3M_KF

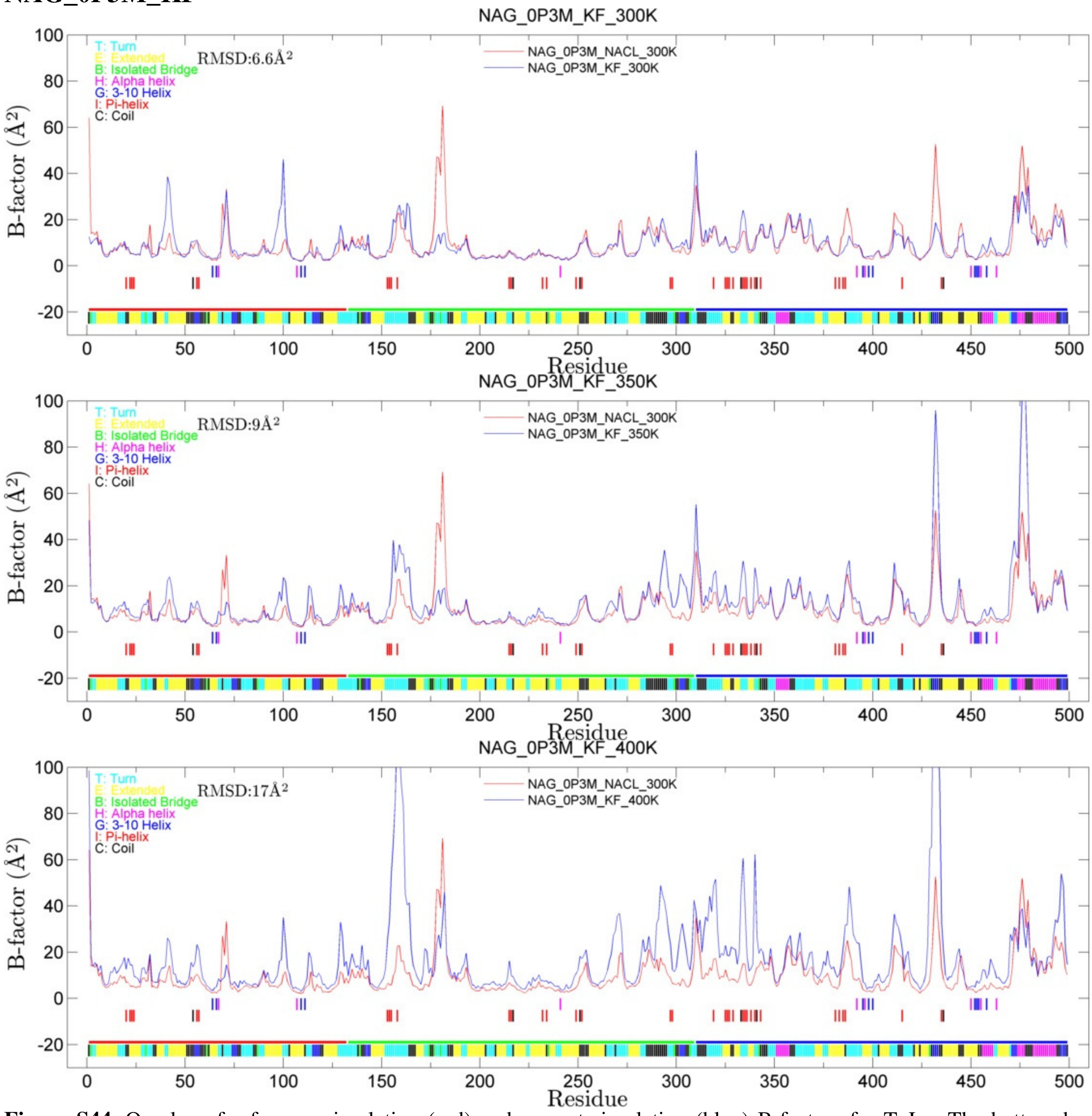

Figure S44. Overlay of reference simulation (red) and current simulation (blue) B-factors for TvLα. The bottom bar indicates secondary structure, with color codes in the upper-left legend. The horizontal three-colored line immediately above the secondary structure bar denotes the three laccase domains (D1: Red, D2: Green, D3: Blue). Above the domain line, short black vertical lines indicate NAG-positions, and red lines denote residues initially 4.5 Å from NAG. Blue and magenta lines indicate residues directly coordinating Cu and immediate structural neighbors of Cu-binding residues, respectively.

noNAG_0P3M_KF

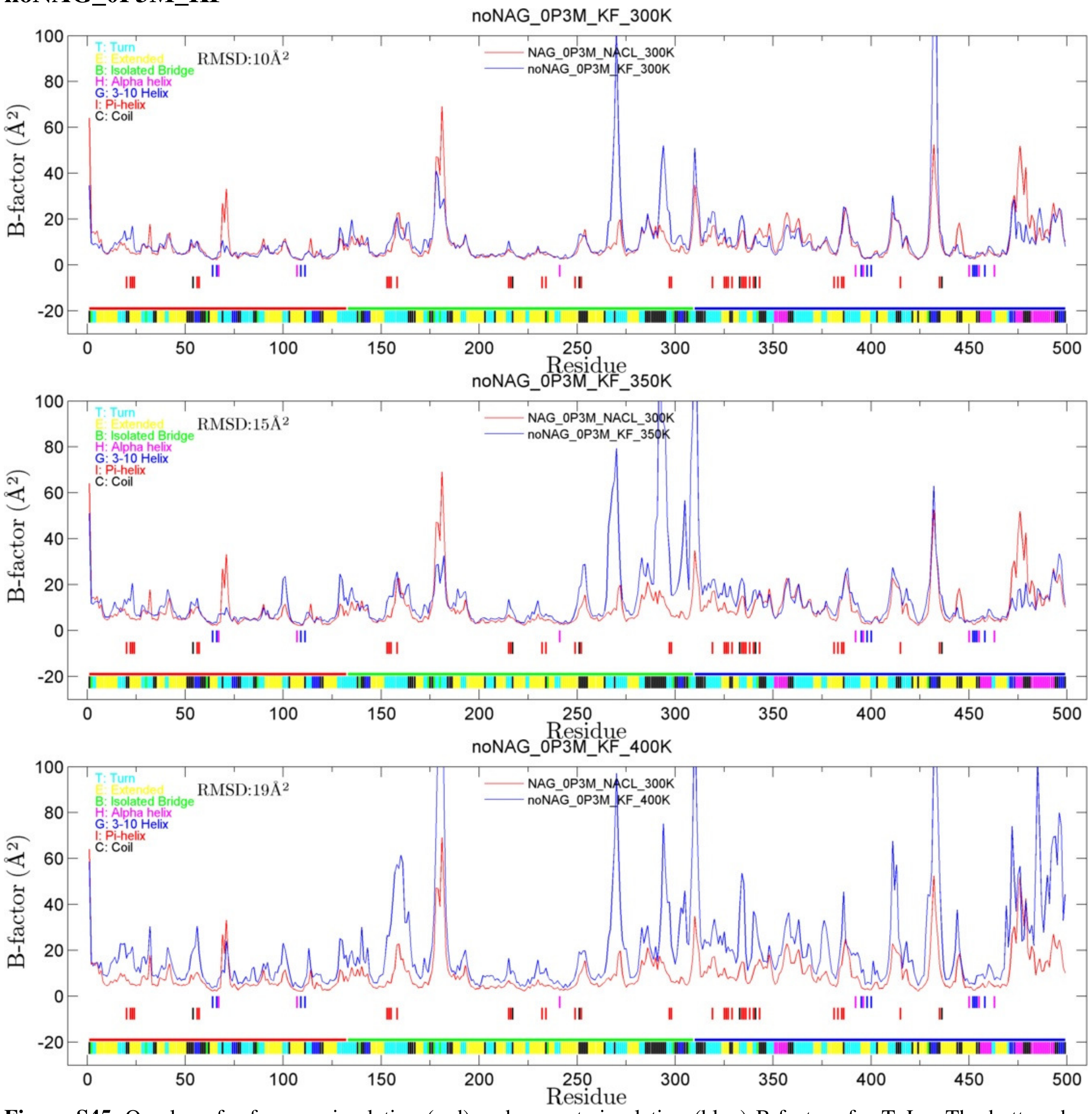

Figure S45. Overlay of reference simulation (red) and current simulation (blue) B-factors for TvLα. The bottom bar indicates secondary structure, with color codes in the upper-left legend. The horizontal three-colored line immediately above the secondary structure bar denotes the three laccase domains (D1: Red, D2: Green, D3: Blue). Above the domain line, short black vertical lines indicate NAG-positions, and red lines denote residues initially 4.5 Å from NAG. Blue and magenta lines indicate residues directly coordinating Cu and immediate structural neighbors of Cu-binding residues, respectively.

NAG_1P2M_NACL

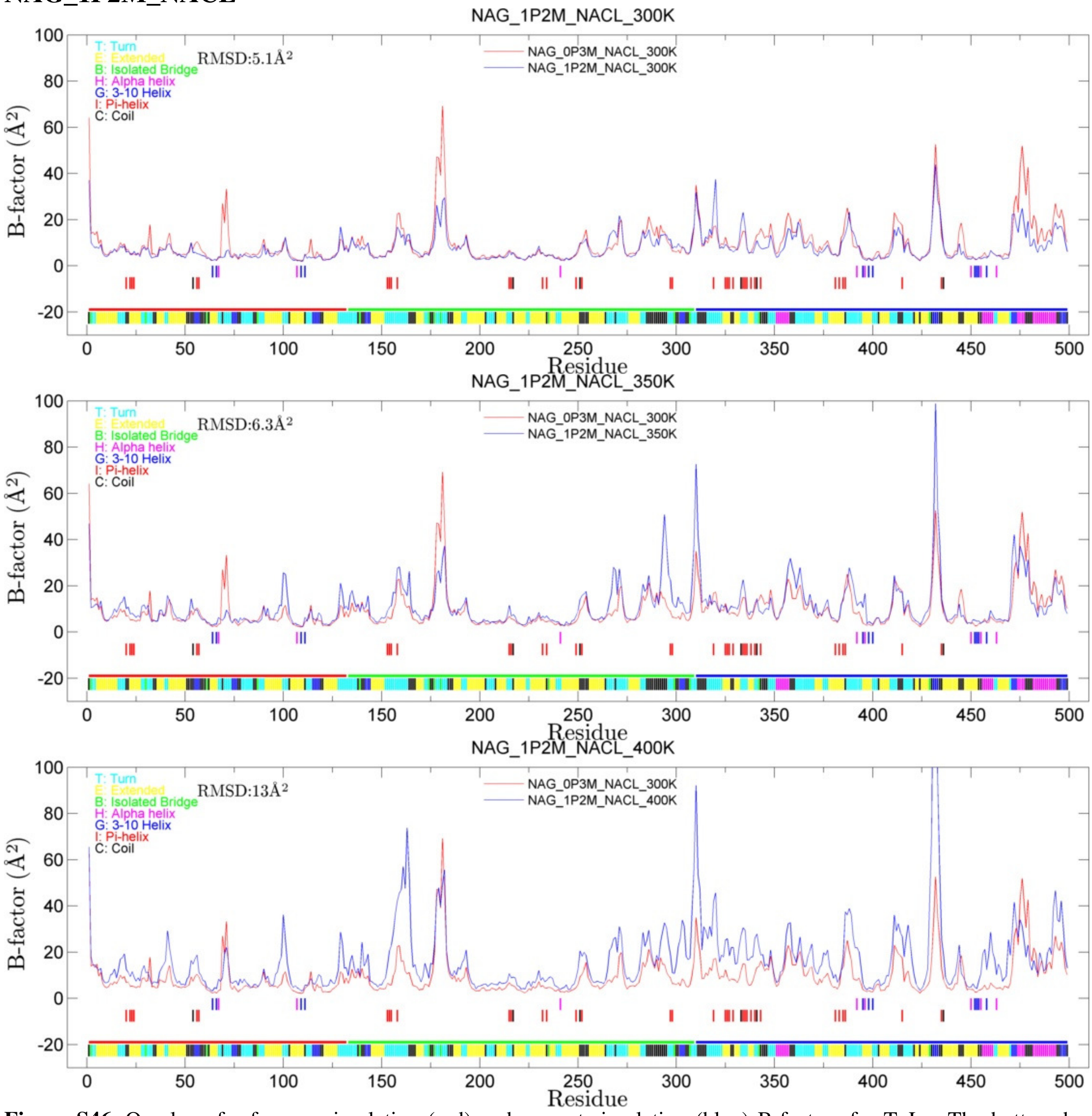

Figure S46. Overlay of reference simulation (red) and current simulation (blue) B-factors for TvLα. The bottom bar indicates secondary structure, with color codes in the upper-left legend. The horizontal three-colored line immediately above the secondary structure bar denotes the three laccase domains (D1: Red, D2: Green, D3: Blue). Above the domain line, short black vertical lines indicate NAG-positions, and red lines denote residues initially 4.5 Å from NAG. Blue and magenta lines indicate residues directly coordinating Cu and immediate structural neighbors of Cu-binding residues, respectively.

noNAG_1P2M_NACL

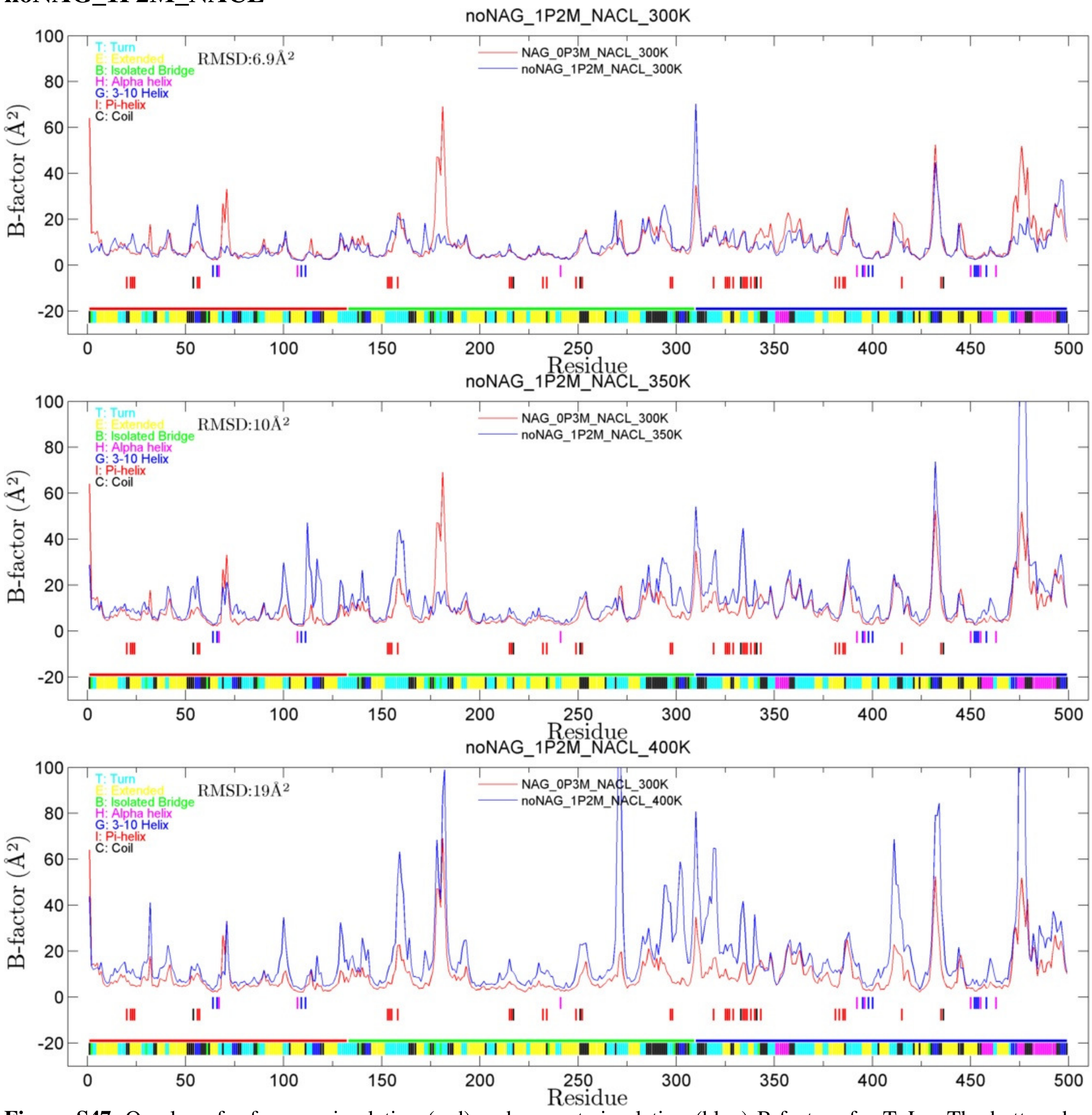

Figure S47. Overlay of reference simulation (red) and current simulation (blue) B-factors for TvLα. The bottom bar indicates secondary structure, with color codes in the upper-left legend. The horizontal three-colored line immediately above the secondary structure bar denotes the three laccase domains (D1: Red, D2: Green, D3: Blue). Above the domain line, short black vertical lines indicate NAG-positions, and red lines denote residues initially 4.5 Å from NAG. Blue and magenta lines indicate residues directly coordinating Cu and immediate structural neighbors of Cu-binding residues, respectively.

NAG_1P2M_KF

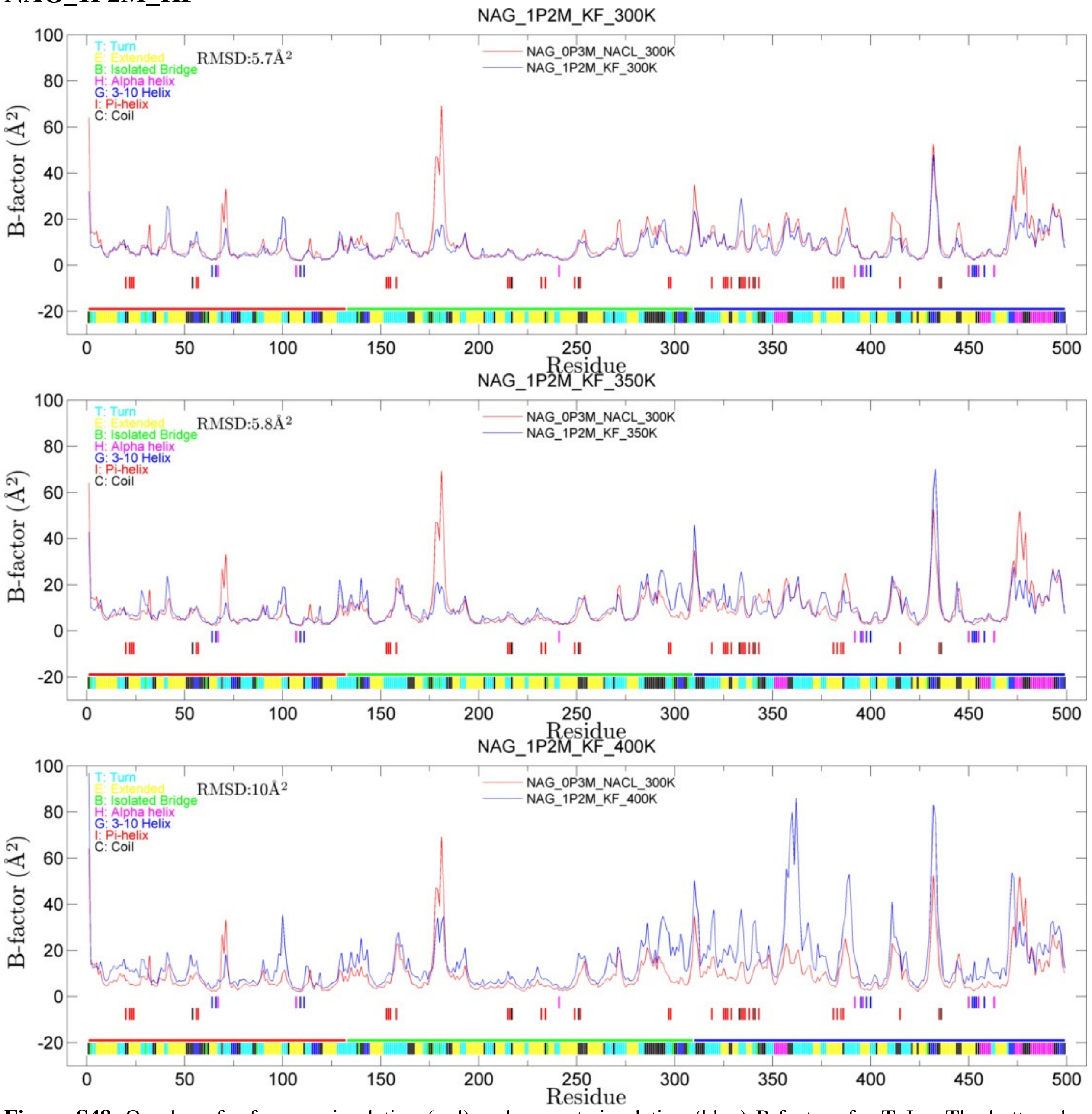

Figure S48. Overlay of reference simulation (red) and current simulation (blue) B-factors for TvLα. The bottom bar indicates secondary structure, with color codes in the upper-left legend. The horizontal three-colored line immediately above the secondary structure bar denotes the three laccase domains (D1: Red, D2: Green, D3: Blue). Above the domain line, short black vertical lines indicate NAG-positions, and red lines denote residues initially 4.5 Å from NAG. Blue and magenta lines indicate residues directly coordinating Cu and immediate structural neighbors of Cu-binding residues, respectively.

noNAG_1P2M_KF

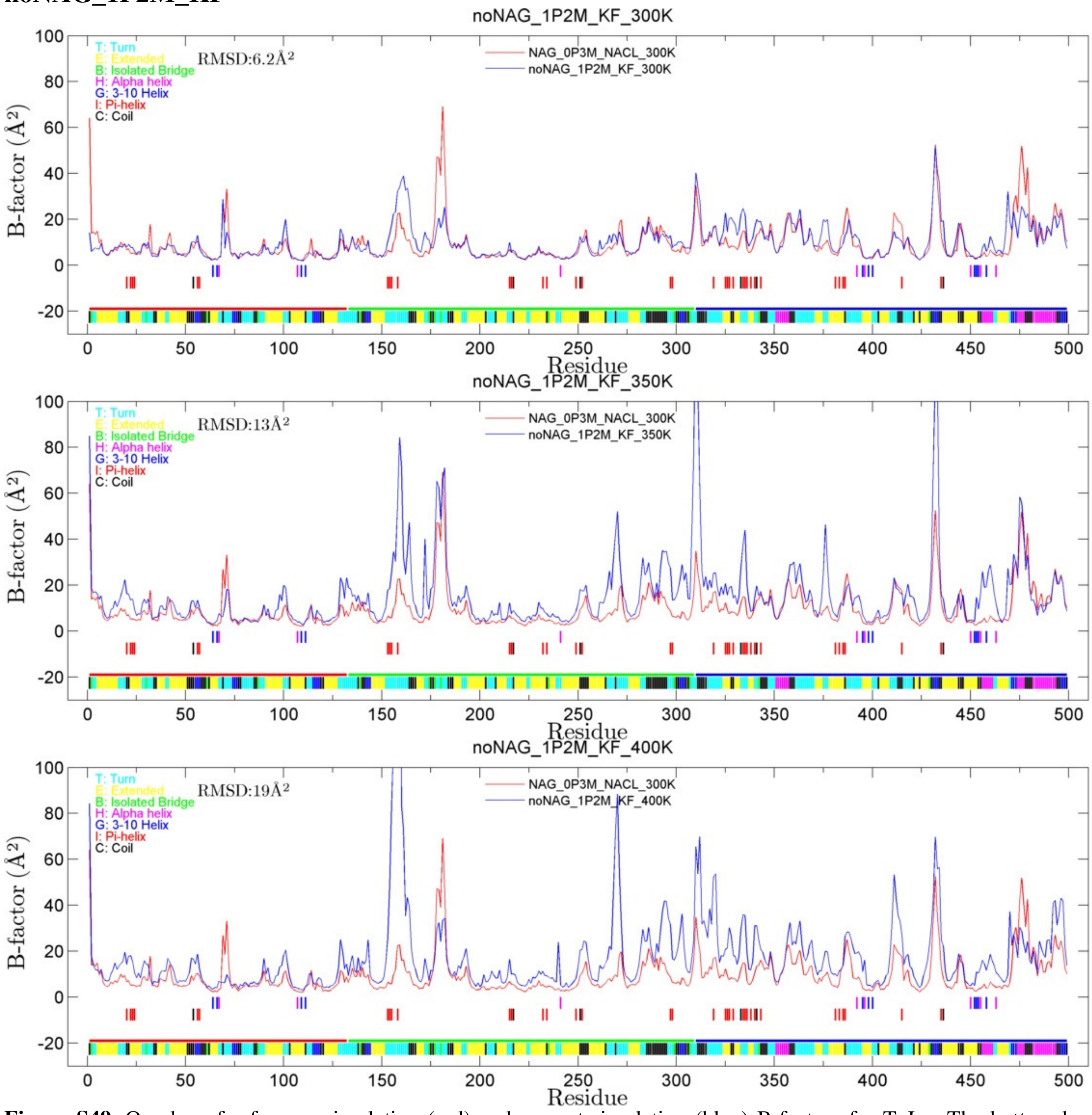

Figure S49. Overlay of reference simulation (red) and current simulation (blue) B-factors for TvLα. The bottom bar indicates secondary structure, with color codes in the upper-left legend. The horizontal three-colored line immediately above the secondary structure bar denotes the three laccase domains (D1: Red, D2: Green, D3: Blue). Above the domain line, short black vertical lines indicate NAG-positions, and red lines denote residues initially 4.5 Å from NAG. Blue and magenta lines indicate residues directly coordinating Cu and immediate structural neighbors of Cu-binding residues, respectively.

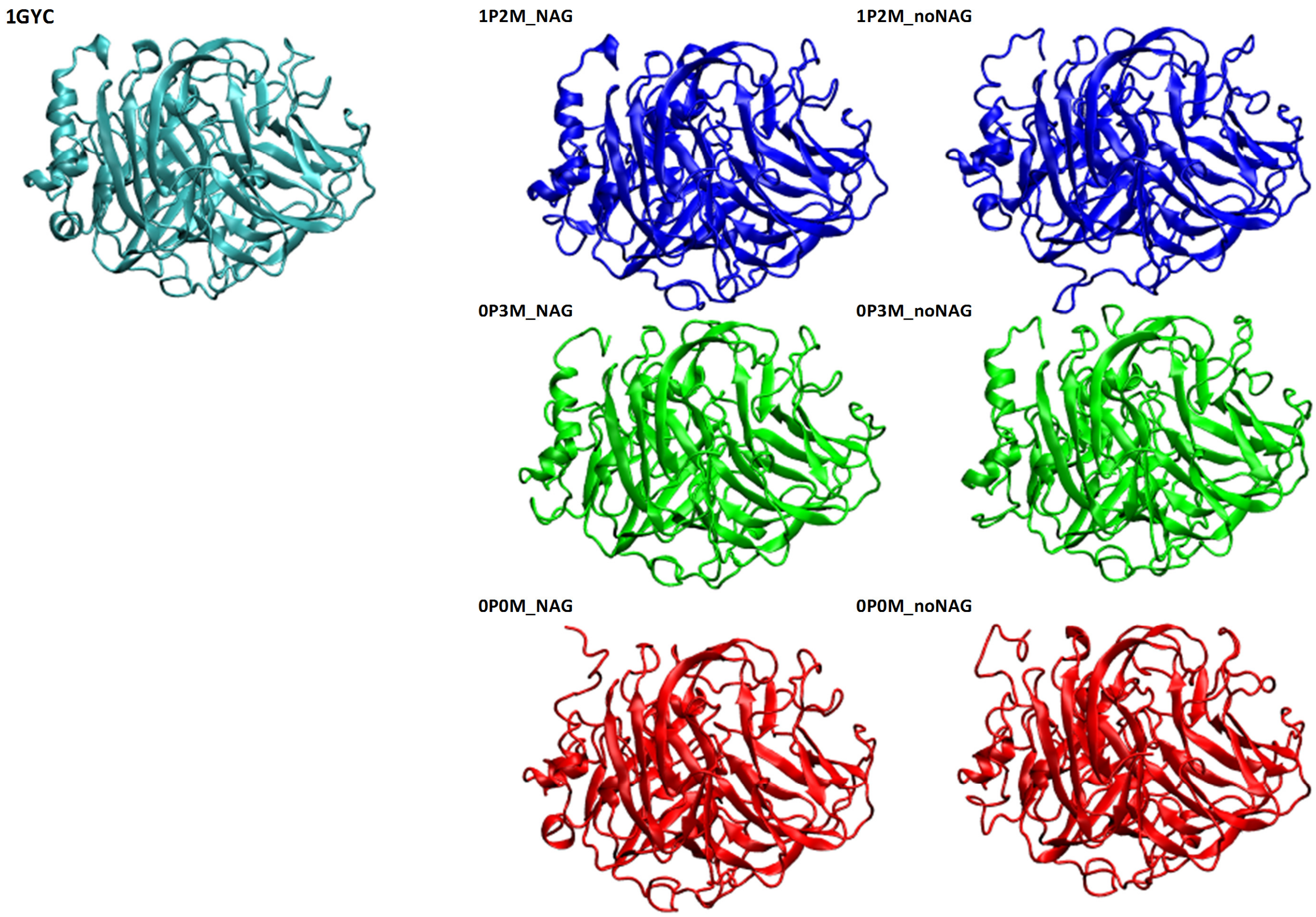

Figure S50. Last snapshots from extended (10 ns) NVT simulations at 400 K of TvLα with (left) and without (right) glycosylation in NaCl backgrounds of 0 M (red), 0.3 M (green) and 1.2 M (blue). For reference, the TvLα crystal structure (PDB ID: 1GYC) is included in the upper left corner.

Table S6. Loss of persistent hydrogen bonds for glycosylated TvL in 0.3 M NaCl due to the 300 K to 350 K temperature increase. Hydrogen bonds lost in both the 0.3 M and 1.2 M NaCl background are indicated in bold.

**acceptor**	**donor**	**location**
GLY41	VAL99	β-sheet end
**GLN45**	**PRO4**	**first β-sheet, exposed**
SER60	GLN499	C-terminal, tethering HB
ASP77	ASN74	α-helical segment, burried
SER110	LEU120	β-sheet end, burried
HIS111	SER62	β-strand start, end of loop
ASP118	GLN115	helical segment, burried
GLY119	TYR116	helical segment, burried
**ALA134**	**ASP131**	**loop, exposed**
VAL139	ARG195	β-sheet start, exposed
TRP151	ALA168	β-sheet turn, exposed
LEU158	ALA155	Loop, exposed
THR210	ASN262	β-sheet start/end, exposed
ILE274	PHE270	loop, exposed
**ASN304**	**ILE301**	**loop/α-helical segment, exposed**
LEU305	GLU302	loop/α-helical segment, exposed
**SER370**	**PRO367**	**turn, exposed**
**THR383**	**LEU326**	**β-sheet**
ASP456	CYS205	helix to loop HBond
**ASP492**	**CYS488**	**α-helix, C-terminal**

Table S7. Loss of persistent hydrogen bonds for glycosylated TvL in 1.2 M NaCl due to the 300 K - 350 K temperature increase. Hydrogen bonds lost in both the 0.3 M and 1.2 M NaCl background are indicated in bold.

**acceptor**	**donor**	**location**
VAL27	VAL30	loop, exposed
SER33	ARG121	β-sheet start
ASP42	LYS39	turn, exposed
**GLN45**	**PRO4**	**first β-sheet, exposed**
THR51	VAL10	β-sheet end, exposed
HIE66	TRP107	HIE66 involving T3
ASP118	THR114	helical segment, burried
**ALA134**	**ASP131**	**loop, exposed**
THR180	SER177	loop, exposed
ALA241	SER202	turn, burried pointing to T3 site
**ASN304**	**ILE301**	**loop/α-helical segment, exposed**
ASP364	THR361	loop, exposed
LEU365	THR361	loop, exposed
**SER370**	**PRO367**	**turn, exposed**
**THR383**	**LEU326**	**β-sheet**
TYR491	LEU487	α-helix, C-terminal
**ASP492**	**CYS488**	**α-helix, C-terminal**
LEU494	TYR491	C-terminal

Electrostatic Energy Analysis

Electrostatics of Persistent Hydrogen Bonds Lost from 300 K to 350 K in both 0.3 M and 1.2 M NaCl

Table S8. Simulation averaged electrostatic interaction energy between the residue pairs listed in bold in Table S6 and S7 and persistence of backbone hydrogen bonds for the same residues. The analysis was carried out on the last 2 ns of the 300 K, 350K, and 400 K simulations of glycosylated TvL in 0.3M NaCl. Hydrogen bonds in secondary structure and loop regions are indicated in red and green, respectively.

	**HB**		**300 K**			**350 K**			**400 K**		
**Location**	**Acceptor**	**Donor**	**HB persist. (%)**	**$E_{Coul.}$ (kcal/mol)**	**Stdev. $E_{Coul.}$**	**HB persist. (%)**	**$E_{Coul.}$ (kcal/mol)**	**Stdev. $E_{Coul.}$**	**HB persist. (%)**	**$E_{Coul.}$ (kcal/mol)**	**Stdev. $E_{Coul.}$**
α-helix, C-terminal	Asp492	Cys488	65	-1.98	1.55	42	-0.75	2.10	25	0.29	2.23
first β-sheet	Gln45	Pro4	92	-5.10	1.16	38	-1.67	1.42	37	-1.60	1.31
β-sheet	Thr383	Leu326	68	-2.04	2.02	41	-1.16	2.25	50	-1.95	3.06
loop	Ala134	Asp131	61	-5.61	1.38	39	-5.81	1.66	43	-5.71	1.77
loop	Asn304	Ile301	69	-3.84	1.03	44	-3.06	1.57	16	-0.84	1.64
turn	Ser370	Pro367	54	-5.11	1.82	50	-5.51	1.71	40	-4.48	1.92

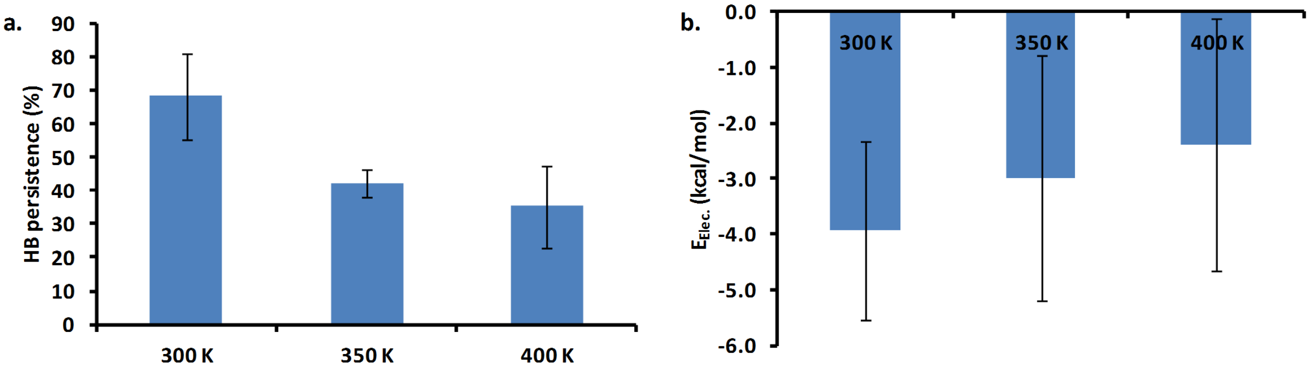

Figure S51. Backbone hydrogen bond persistence (**a**) and electrostatic interaction (**b**) between the involved residues, averaged across the labile hydrogen bond pairs marked in Table S6 and Table S7. Standard deviations are indicated with error-bars.

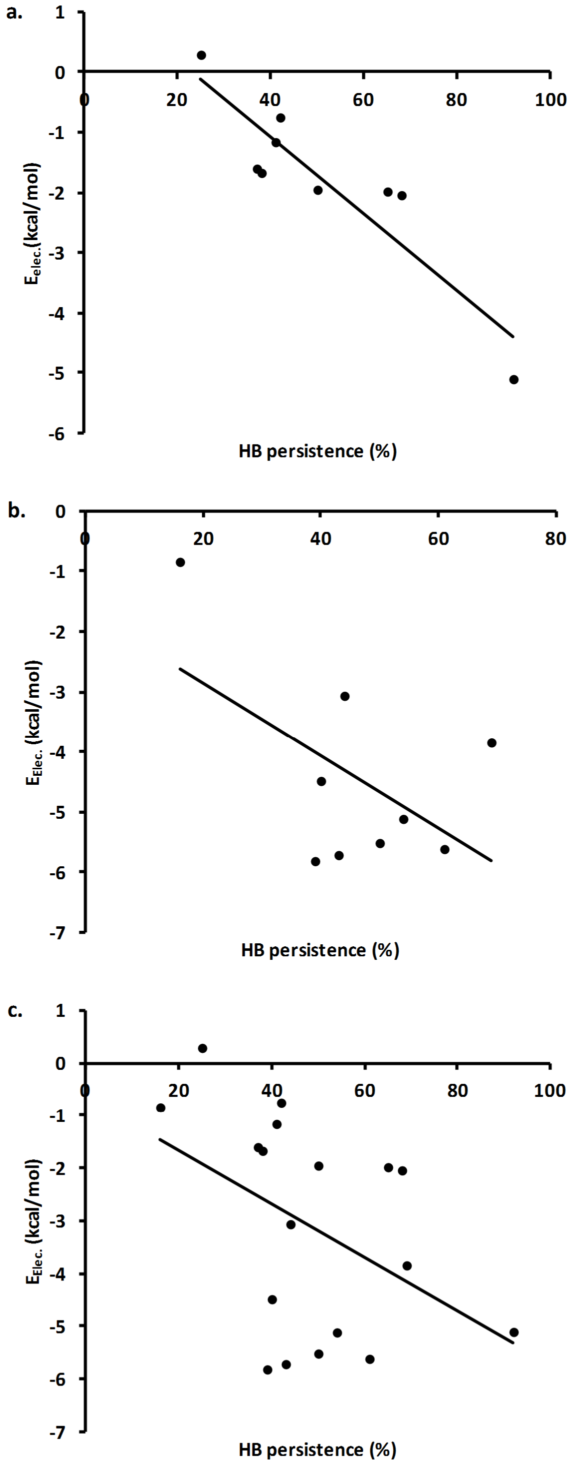

Figure S52. Correlation between backbone hydrogen bond persistence (%) and electrostatic energy (kcal/mol) evaluated between the entire residues for (**a**) residue pairs in structured parts of the protein (red in Table S8), (**b**) residue pairs in loosely structured parts of the protein (green in Table S8), and (**c**) residue pairs in both structured and unstructured parts of the protein.

Electrostatics of C-terminal Helix Disruption at Zero Ionic Strength and High Temperature

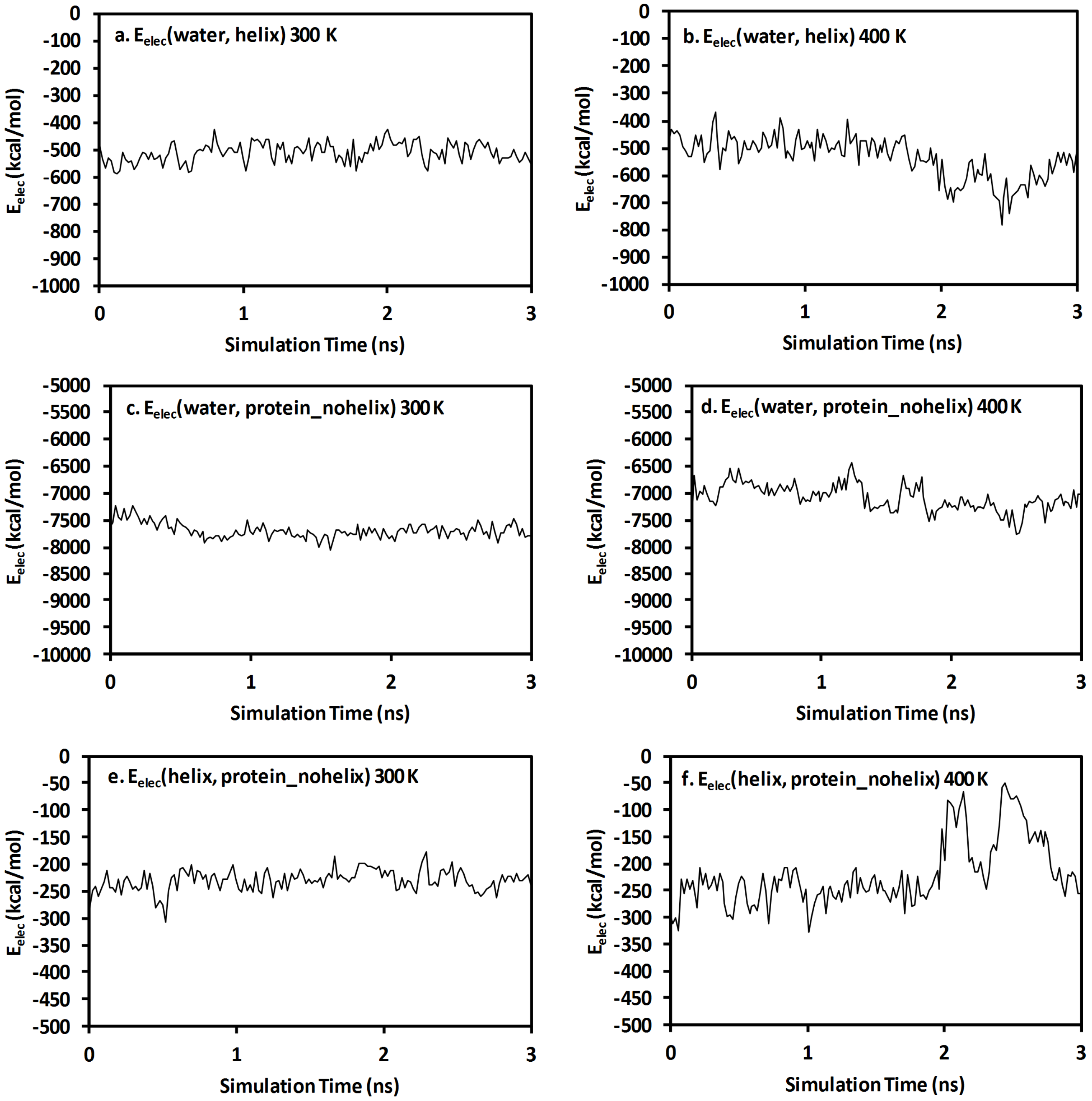

Figure S53. Electrostatic analysis of the C-terminal unfolding observed for glycosylated TvL in zero ionic background at 400 K (b, d, f), but not at 300 K (a, c, e). The time series show electrostatic interactions between three groups: "water" consisting of all TIP3P molecules, "helix" consisting of the last C-terminal residues 489 - 499, and "protein_nohelix" consisting of the remainder of the protein.

Persistence of Backbone Hydrogen Bonds in Extended Simulations

As discussed in the article and above (under Extended Reference Simulations), extended simulations were made in 0.3 M NaCl background for TvL at 300 K with glycosylation (NAG_0P3M_NACL_300K), and without glycosylation K (noNAG_0P3M_NACL_300K). The corresponding simulations at 400 K with and without glycosylation (NAG_0P3M_NACL_400K and noNAG_0P3M_NACL_400K) were also extended.

The number of persistent backbone hydrogen bonds (HB) was calculated for the 500 MD snapshots between t = 10 ns and t = 20 ns in each extended molecular dynamics trajectory (Table S9). With reference to the RMSD plot for the simulations (Figure 1 in the article main text and Figure S3 in Supporting Information), it is seen that well-behaved RMSD curves are associated with a larger number of persistent HB. Thus it is only reasonable to compare HB numbers from simulations with equally well-behaved RMSD curves.

The extended noNAG_0P3M_NACL_300K simulation yields 164 HB. However, in three out of four simulations with new seeds, the extended NAG_0P3M_NACL_300K simulations have more HB (165 - 167) than noNAG. This is in agreement with the numbers of persistent HB in the 3 ns NVT simulations for NAG (165) and noNAG (162).

The temperature effect is substantial also in the extended simulations: At 400 K, NAG and noNAG have 142 and 144 HB, respectively. In the original 3 ns NVT simulations NAG and noNAG had 148 and 147 HB, respectively. Thus the longer simulation time has decreased the number of persistent hydrogen bonds by ~5 in the high temperature case.

In conclusion, the analysis of hydrogen persistence for the longer simulations from new seeds agrees with the major conclusions from the analysis of 3 ns NVT simulations. In particular, the increase in temperature from 300 K to 400 K is associated with a marked reduction of persistence HB, whereas the presence or absence of NAG has a more subtle effect.

Table S9. Persistent backbone hydrogen bonds (HB) calculated for the 500 MD snapshots between t = 10 ns and t = 20 ns in extended NVT simulations.

**extended simulation**	**# persistent HB**
NAG_0P3M_NACL_300K_Seed1	155
NAG_0P3M_NACL_300K_Seed2	167
NAG_0P3M_NACL_300K_Seed3	170
NAG_0P3M_NACL_300K_Seed4	165
noNAG_0P3M_NACL_300K	164
NAG_0P3M_NACL_400K	142
noNAG_0P3M_NACL_400K	144
